# Efficient Small-Molecule Reversal Agents for Anticoagulant Fondaparinux

**DOI:** 10.1021/acsptsci.4c00747

**Published:** 2025-04-29

**Authors:** Daniel Carbajo, Yolanda Pérez, Gabriela F. Castelo, Eva Prats, Jordi Bujons, Ignacio Alfonso

**Affiliations:** † Department of Biological Chemistry, 203230 Institute for Advanced Chemistry of Catalonia, IQAC−CSIC, Jordi Girona 18-26, 08034 Barcelona, Spain; ‡ NMR Facility, Institute for Advanced Chemistry of Catalonia, IQAC−CSIC, Jordi Girona 18-26, 08034 Barcelona, Spain; § Animal Facility, 73039 Research and Development Center (CID-CSIC), Jordi Girona 18-26, 08034 Barcelona, Spain

**Keywords:** anticoagulant, fondaparinux, antidote, spermine, molecular recognition

## Abstract

Fondaparinux is a highly anionic synthetic heparinoid pentasaccharide used as an anticoagulant for specific clinical conditions and surgeries. As a non-natural small-molecule drug, it presents pharmacokinetic and pharmacodynamic advantages, as well as high stability and low immunogenicity, when compared with different forms of heparin. However, its broader usage is hampered by different factors like price, existence of alternative anticoagulants, or, specifically in this case, the lack of an effective antidote that is highly recommendable for avoiding uncontrolled bleeding. In this work, we describe two synthetic small molecules derived from spermine (3AC and 3FF) that efficiently revert the anticoagulant activity of fondaparinux. In an *in vitro* enzymatic assay related to blood coagulation, the spermine derivatives show potent activity as fondaparinux antidotes, with higher activity than ciraparantag (a small molecule in the clinical phase as an anticoagulant antidote) and much higher activity than protamine, the only approved antidote for unfractioned heparin but inefficient against fondaparinux. Remarkably, naked-eye *ex vivo* tests demonstrated their efficacy in freshly extracted mice blood. Mechanistic studies show that both small molecules strongly bind fondaparinux in buffered water, as detected by fluorescence and NMR spectroscopy and confirmed by molecular dynamics simulations. Thus, these spermine derivatives are promising reversal agents against heparinoid anticoagulants with a wide range of molecular weights, overcoming the drawbacks of those antidotes based on biomacromolecules.

## Graphical abstract

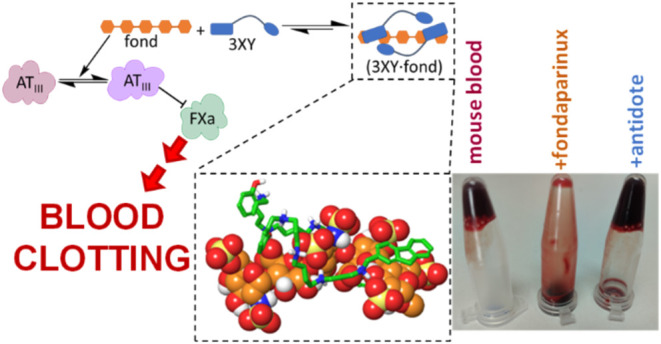

Fondaparinux^1–4^ (fond, Figure 1) is a synthetic pentasaccharide used as an anticoagulant to prevent deep vein thrombosis, which would lead to pulmonary embolism in patients after certain types of surgery, like hip or knee surgery or replacement, as well as abdominal surgery.^5–7^ Its chemical structure resembles the repeating unit of broadly used heparin,^8,9^ while its smaller molecular size presents some advantageous pharmacokinetics and pharmacodynamics,^10^ such as a faster pharmacological effect and more efficient clearance through the kidney into urine.^2^ Moreover, it shows highly homogeneous composition, improved stability, and lower immunogenicity, as compared with different forms of heparin.^5^ Thus, fond rarely produces thrombocytopenia, which is one of the most common complications of the use of heparin.^11,12^ The highly sulfated structure of fond is composed of five saccharide rings as the minimal expression of the binding epitope of heparinoids to antithrombin III (AT_III_),^11,13^ which makes fond an indirect inhibitor of coagulation factor Xa (FXa), a key serine protease within the blood coagulation cascade.^14^ However, this short epitope does not bind protamine (Figure 1), which is the only approved antidote to unfractioned heparin (UFH).^15^ A structurally related permethylated pentasaccharide anticoagulant idraparinux^16,17^ (Figure 1) was developed to increase efficacy and for prolonged treatments, although uncontrolled bleeding was observed in clinical studies. To solve this problem, idrabiotaparinux was designed for which the implemented biotin conjugation allows its elimination from blood circulation by administration of avidin.^18–20^ This idrabiotaparinux/avidin marriage illustrates the high interest in developing low-molecular-weight anticoagulants^21,22^ in parallel with their necessary antidotes.^10,23^ Whereas fond is a drug in current use (as injectable sodium salt solution, i.e., Arixtra or Forixtra),^6,11^ idraparinux and idrabiotaparinux did not reach the clinic despite their higher anticoagulation potential.^19,20^ As a drawback, the lack of approved antidote^24^ for fond has hampered its broader clinical usage, since efficient reversal agents are essential for a safer prescription of anticoagulants in order to prevent high-risk uncontrolled bleeding. Actually, a biotinylated fond-derivative has been recently reported, which also allows reversing its anticoagulation effects using avidin as an antidote in promising *in vitro* assays.^25^ To the best of our knowledge, there is only one small molecule derived from arginine (PER977 or ciraparantag^26–28^ (cir) in Figure 1) in phase II^29^ clinical trials as an universal antidote for different anticoagulants, which was also reported to revert the anticoagulation effect of fond.^28^ However, biophysical studies showed a relatively weak noncovalent cir–fond interaction,^30^ which suggests a different and still unclear mechanism of action. Sequestering of fond has also been carried out with larger molecules and (bio)­polymers like modified cyclodextrins,^31^ dendrigraft poly-l-lysine,^32^ cationic macromolecular nanoparticles,^33^ porous polymeric materials,^34^ diblock copolymers,^35^ or recombinant antithrombin mutants.^36^

**Figure 1:**
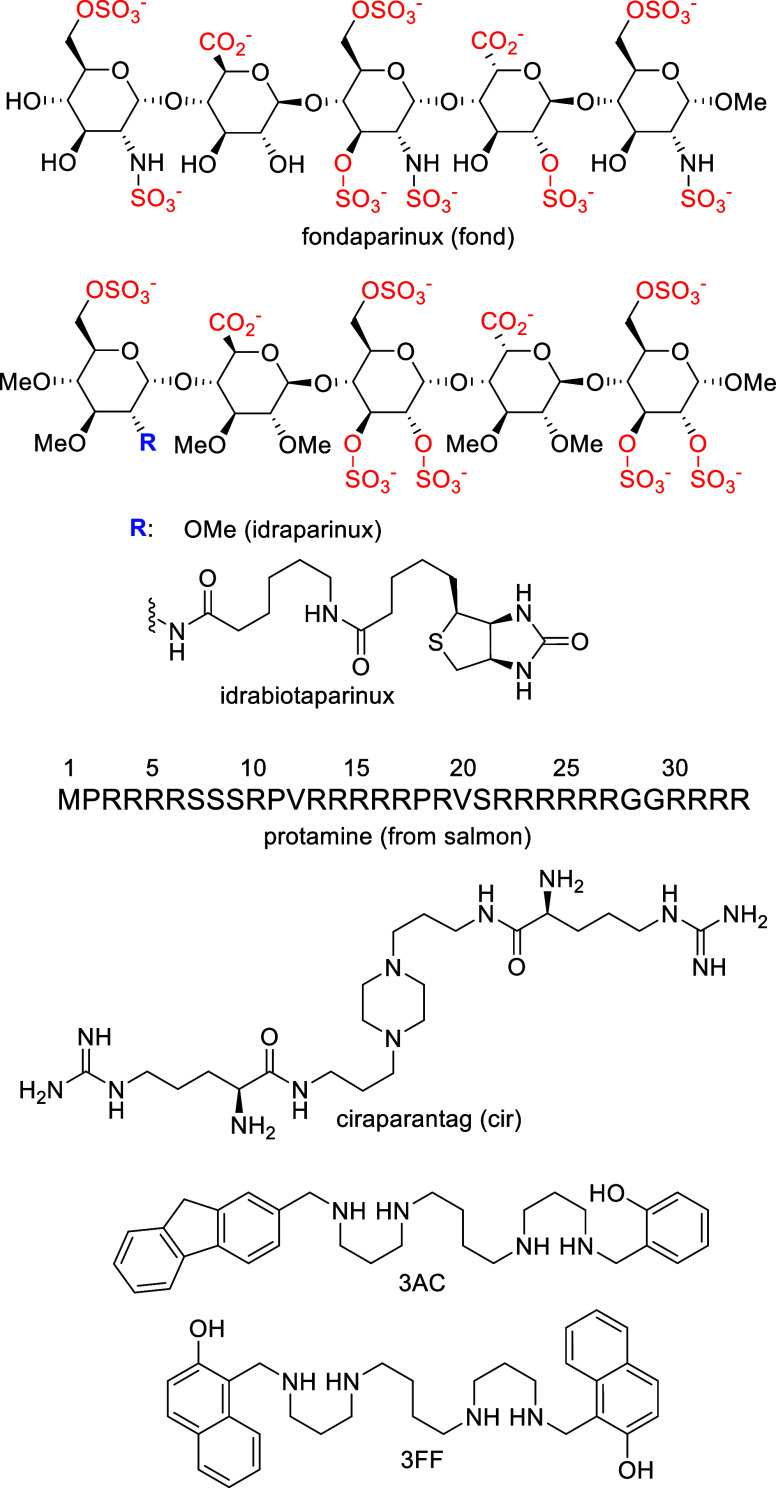
Chemical structures of anticoagulants, fondaparinux (fond), idraparinux, and idrabiotaparinux, as well as of reversal agents, protamine (from salmon, one-letter code peptide sequence), ciraparantag (cir), 3AC, and 3FF.

On the other hand, we have recently reported on the dynamic combinatorial optimization of new small molecules derived from spermine that are strong ligands to heparin.^37,38^ Two of these new molecules (3AC and 3FF, Figure 1) showed excellent *in vitro*, *ex vivo*, and *in vivo* activities as reversal agents of heparin anticoagulant.^38^ Biophysical and structural analyses of the mode of binding of these molecules to heparin suggested that their size and conformation could fit the geometrical restrictions imposed by the short length of fond, possibly forming noncovalent complexes in solution as a reversal mechanism against this synthetic anticoagulant. In the current contribution, we aim to expand the potential of these antidotes to the smallest possible heparinoid used in the clinic. In this way, we will demonstrate that the spermine derivatives 3AC and 3FF are efficient reversal agents for a broad family of heparin-derived anticoagulants, from UFH or LMWH to fond, for which no antidote is currently available.

## Results and Discussion

### Synthesis and Characterization of the Studied Molecules

Spermine derivatives used in this work were synthesized as previously described.^38^ Ciraparantag was prepared by solution-phase peptide synthesis from commercially available precursors.^30^ All of the compounds were characterized by NMR spectroscopy and high-resolution mass spectrometry and purified by reverse-phase chromatography, rendering >95% purity by analytical HPLC (Figure S1).

### In Vitro Activity Assays

The activity of two spermine derivatives, 3AC and 3FF, as fond antidotes was evaluated with an *in vitro* enzymatic assay related to blood coagulation, as previously described (Figure 2A).^37,38^ In brief, fond promotes a conformational change in AT_III_ toward an efficient inhibitor of FXa.^39^ Addition of a ligand of fond hinders the inhibitory action of AT_III_ restoring the FXa hydrolytic activity, which has been used as an indirect measurement of antifond behavior. Thus, in the absence of fond (black open circles in Figure 2B), FXa shows the maximal activity under the assayed experimental conditions. Addition of fond (15 μM) clearly reduces the hydrolytic activity (gray solid circles in Figure 2B). Both 3AC (blue and purple diamonds) and 3FF (orange and brown triangles) are able to restore the FXa protease activity in a dose–response manner (9 and 15 μM), 3AC being a slightly more potent antidote (Figure 2B).

**Figure 2:**
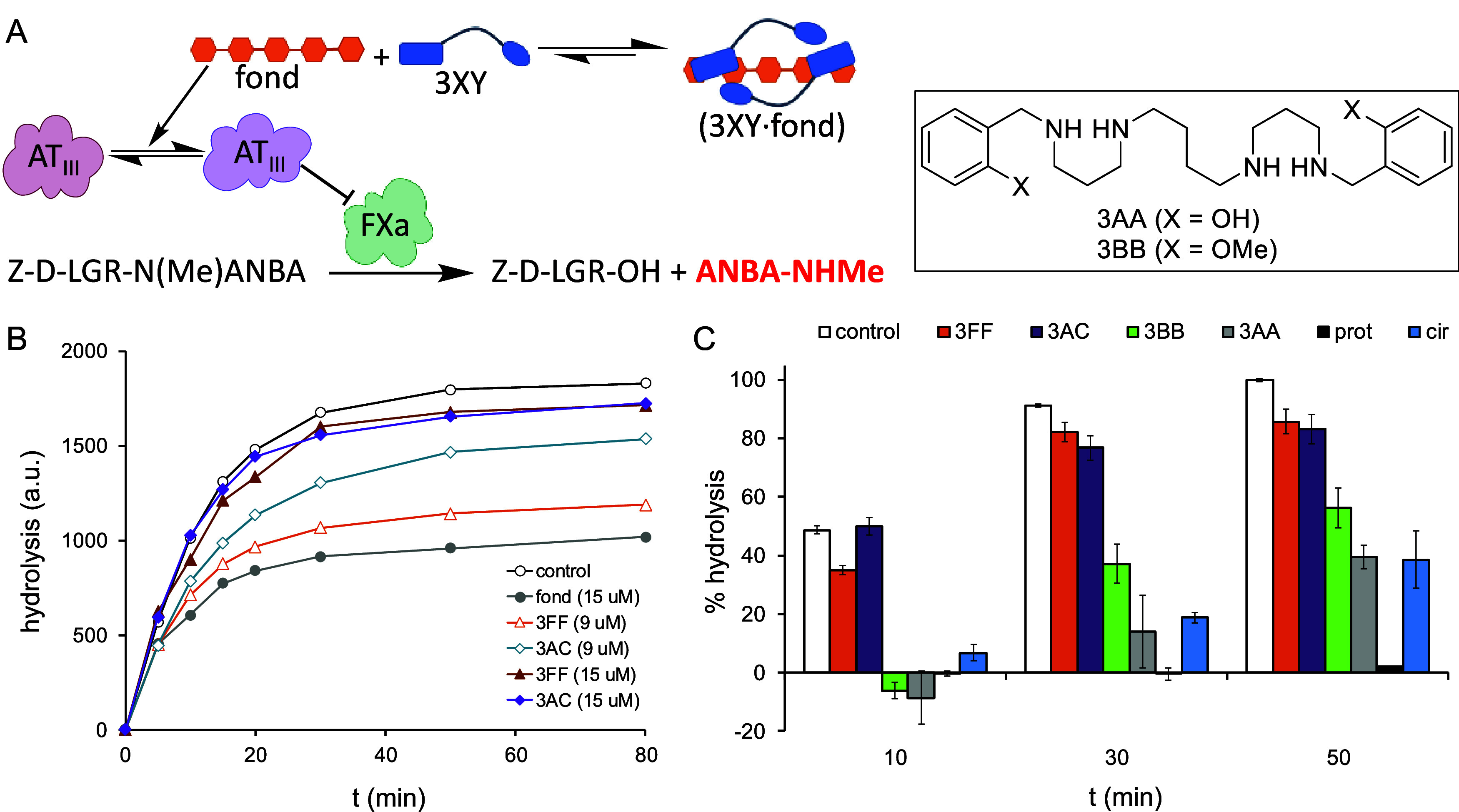
(A) Schematic representation of the *in vitro* enzymatic assay used to test the antifond activity of ligands. (B) Hydrolysis rate of the peptide substrate (measured by HPLC) for samples containing recombinant FXa/AT_III_ as the control (black hollow circles), and additional 15 μM fond (solid gray circles), 15 μM fond + 3FF (9 μM orange hollow triangles or 15 μM brown solid triangles), or 15 μM fond + 3AC (9 μM blue hollow diamonds or 15 μM purple solid diamonds). (C) Plot of percent recovery of peptide substrate hydrolysis ((hydrolysis recovery vs fond/max. hydrolysis recovery vs fond) x 100) from samples containing FXa/AT_III_ at different reaction times: control (white), fond + 3FF (orange), fond + 3AC (purple), fond + 3BB (green), fond + 3AA (gray), fond + prot (black), and fond + cir (blue). In all of the cases, [fond] = [antidote] = 15 μM. All experiments were carried out at least in triplicate.

In order to unravel the corresponding mechanism of action (see below), two structurally related spermine analogues with a much weaker binding to heparinoids (3AA and 3BB,^38,40^ inset in Figure 2) were tested. We also compared the *in vitro* activity with the only clinically approved antidote for UFH (arginine-rich protein protamine, prot) and a small molecule currently in clinical trials as a universal anticoagulant antidote, ciraparantag (cir). To that end, we plotted the percent recovery of peptide substrate hydrolysis at three different reaction times (Figure 2C). Both 3AC and 3FF showed potent activities, leading to almost complete fast recovery up to the control values at equimolecular concentrations (Figure 2C). The slightly faster action of 3AC can be due to the aggregation properties of 3FF that would produce an induction lapse to bind to fond. Besides, similar experiments performed at a lower antidote concentration suggested a higher potency of 3AC (Figure S2). The two ligands (3AA and 3BB, inset in Figure 2) with weaker binding to heparinoids^38^ display lower potency as antidotes. Remarkably, protamine (prot) shows practically no *in vitro* activity against fond, as already described in the literature.^24^ Gratifyingly, both 3AC and 3FF are faster and more potent antidotes than cir, which performs similarly to the spermine derivative ligand (3AA) that is less active in this enzymatic assay (Figure 2C). On the other hand, additional *in vitro* assays using a LMWH showed that 3AC and 3FF are more active than prot and similar to cir^26^ (Figure S3) with this medium-sized anticoagulant. Thus, these results in addition to our previous findings with UFH^38^ identify 3AC and 3FF as antidotes against any kind of heparin-derived anticoagulant with a wide molecular-weight range, more efficient than either the only currently approved drug (protamine) or a promising molecule currently in clinical trials (ciraparantag).

### Ex Vivo Coagulation Assays

Considering the results obtained in the *in vitro* assays, we aimed to test our molecules in a more relevant and challenging environment. To this aim, we performed naked-eye *ex vivo* coagulation assays (Figure 3). Freshly extracted mouse blood samples were treated with fond (100 μM final concentration) in the absence and in the presence of different antidotes (two molar equivalents). Blood coagulation was visually inspected by the simple inversion of the vials. For a fair comparison between different series, all of the experiments were done in parallel with the corresponding control samples of untreated mouse blood (labeled “0” in Figure 3). The anticoagulant effect of fond was readily observed by the free-flowing down of liquid blood (Figure 3A, fond). Both 3AC and 3FF were able to efficiently revert the drug effect, restoring the coagulation to a behavior similar to that of the control samples (Figure 3A). Ciraparantag was also a good antidote, though qualitatively less effective than 3AC/3FF, which is in good agreement with the observed *in vitro* activity. As expected and also observed *in vitro*, protamine fails to be an antidote of fond. The effect of antidotes alone on blood coagulation was also tested (Figure 3B), showing that neither of the three small molecules appreciably changed the coagulation properties. However, prot in the absence of fond acts as an anticoagulant itself, a fact that has been previously described and was attributed to protamine–platelet interactions.^41^ These observations demonstrate the potential of 3AC/3FF as fondaparinux antidotes and also underline some precautions on the use of prot in the clinic.^15^

**Figure 3:**
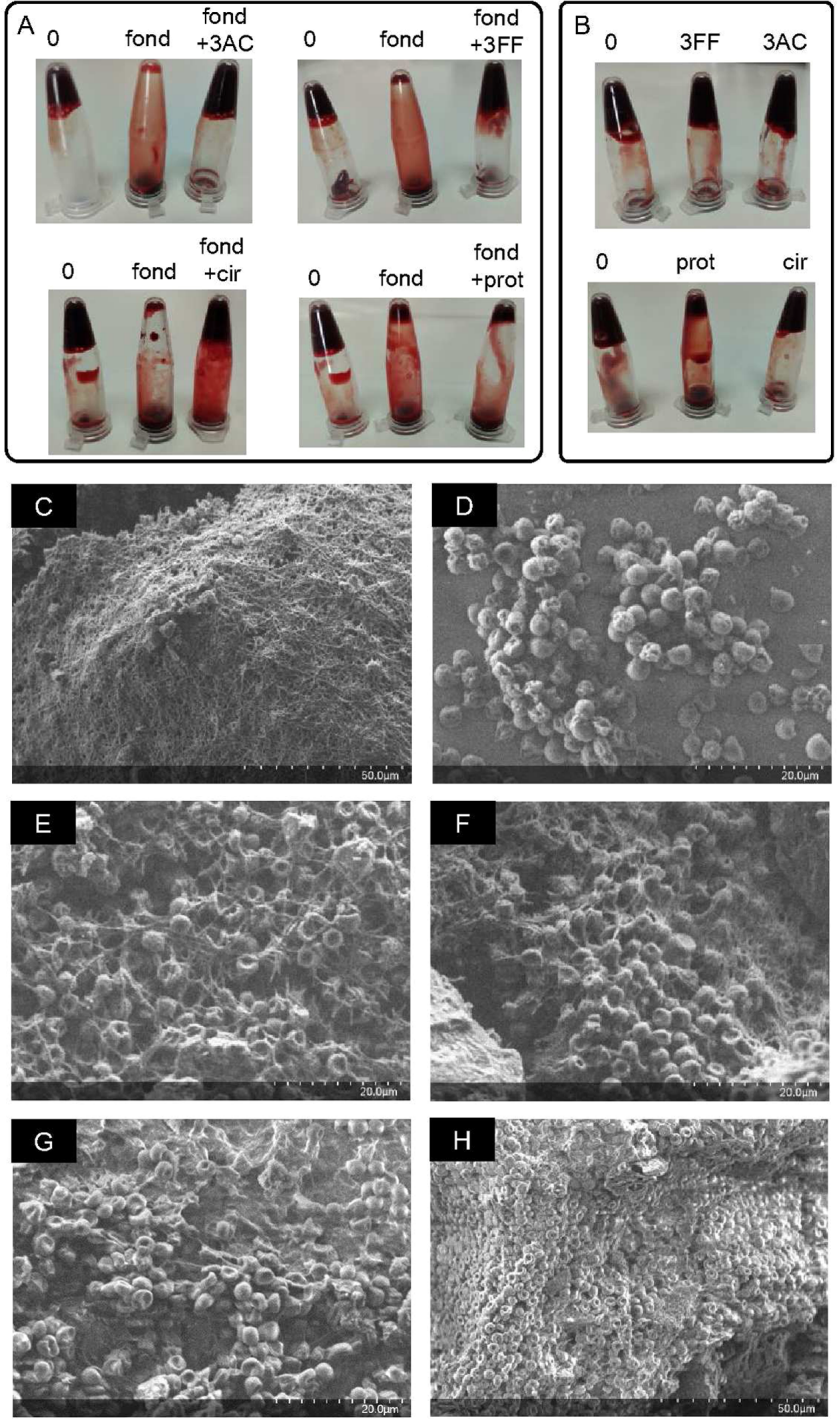
(A) *Ex vivo* coagulation assays with freshly extracted mouse blood (labeled 0 in each panel) in the presence of 100 μM fondaparinux (fond) and 100 μM fond + 200 μM 3AC, 3FF, cir, or prot. (B) Control experiments only containing the corresponding antidotes (200 μM). Scanning electron microscopy (SEM) micrographs with (C) blood clot of the untreated mouse blood sample, or blood treated with (D) fond, (E) fond + 3AC, (F) fond + 3FF, (G) only 3AC, or (H) only 3FF. Corresponding scales are shown in the right bottom corner of each picture.

We also performed scanning electron microscopy (SEM) images of the corresponding mouse blood samples (Figure 3C–H). The SEM image of the untreated blood clot (Figure 3C) clearly shows a dense network of micrometer-sized fibrils completely covering the blood cells. The fondaparinux-treated blood is clean of fibrils, allowing the neat observation of blood cells (Figure 3D). Samples treated with fond and either 3AC (Figure 3E) or 3FF (Figure 3F) show the presence of fibrils responsible for blood coagulation. A close inspection of the images also show that blood cells are intact and embedded within the fibril network. As control experiments, the SEM images of blood clots formed in the presence of only 3AC or 3FF (Figure 3G,H, respectively) show that the aggregated blood cells retained their expected morphology. These *ex vivo* studies additionally support the utility of the two spermine derivatives (3AC and 3FF) as fond antidotes in blood.

### Cell Toxicity and Hemolysis

In order to study the potential toxicity of compounds, we tested the effect of 3AC and 3FF on the viability of HeLa, A549, fibroblasts, and HMEC-1 cells (Figure 4A). Both compounds show low to acceptable cell toxicity at concentrations <100 μM. The slightly higher toxicity of 3FF (especially in fibroblasts and HMEC-1 cell lines) can be due to the nonspecific aggregation of this ligand at the tested concentrations at neutral pH. However, since we proposed to use these compounds as antidotes, their presence without the anticoagulant would mimic cases of overdose or erroneous administration. Accordingly, we also tested the most potent derivative 3AC in the presence of fondaparinux (100 μM) in two selected cell lines, closely related to the vascular system (Figure 4B). The marginal toxicity of 3AC is reduced in the presence of fondaparinux, reflecting a safer situation for the correct antidote usage. Also, the potential hemolytic activity of the two compounds was tested in freshly isolated mouse erythrocytes, showing both essentially nonhemolytic (<2% hemolysis, Figure 4C) and in good agreement with the results qualitatively obtained from the SEM images.

**Figure 4:**
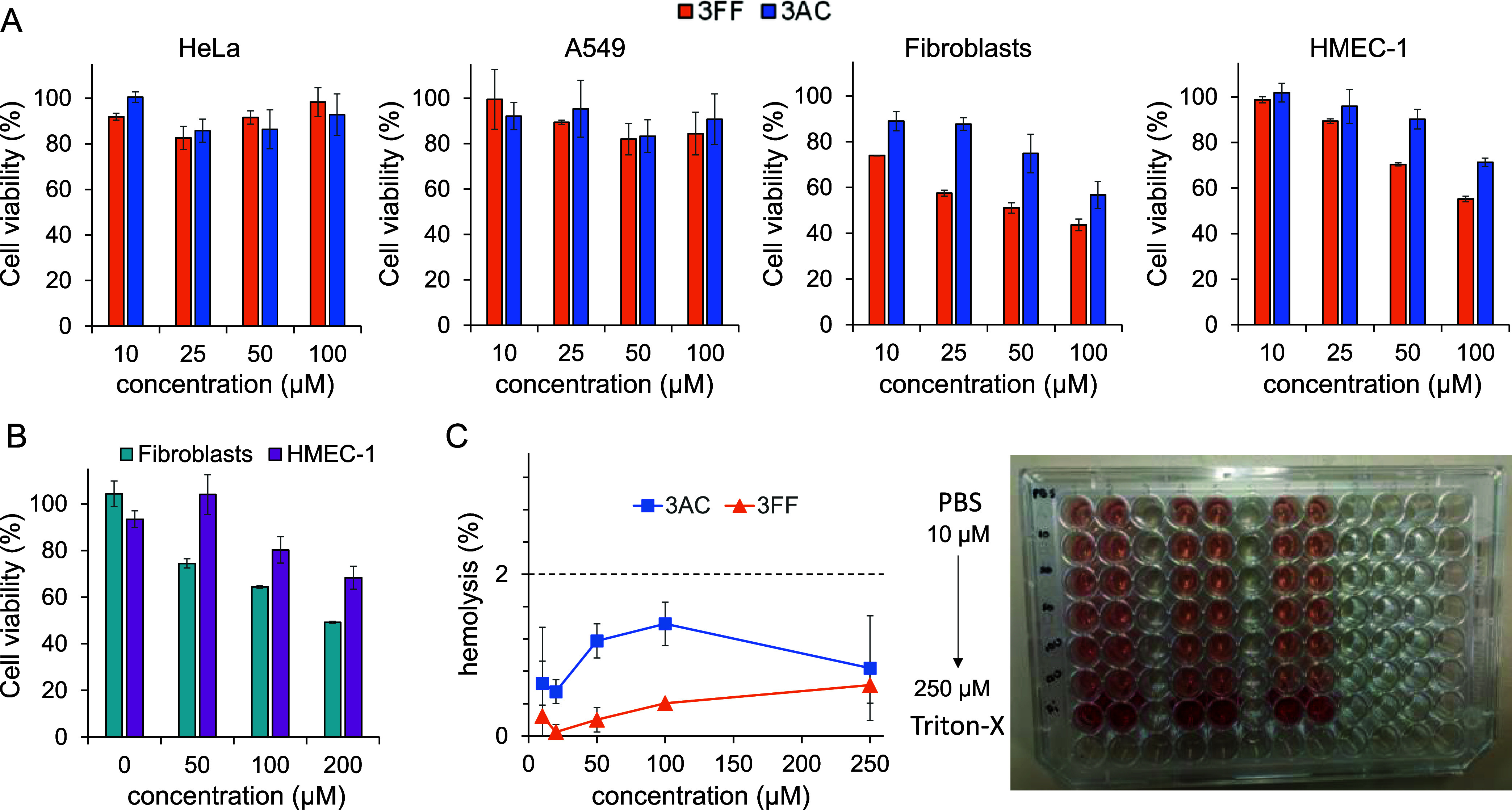
(A) Cell viability experiments for different concentrations of 3FF (orange) and 3AC (blue) in several cell lines. (B) Cell viability tests for different concentrations of 3AC in the presence of 100 μM fondaparinux in two selected cell lines: fibroblasts (light blue) and HMEC-1 (purple). (C) Plot of hemolytic activity of 3FF (orange) and 3AC (blue) performed with freshly isolated mouse erythrocytes with an illustrative picture of the measured samples. Error bars show the corresponding standard deviations within experimental triplicate (see the Experimental Section for details).

### Mechanism of Action

We propose that the antidote activity of the spermine derivatives is due to competitive binding to the fondaparinux drug, as already suggested by the *in vitro* enzymatic assays. In order to confirm this hypothesis, we performed experimental binding studies and theoretical molecular dynamics simulations, leading to the characterization of the corresponding supramolecular species.

### Fluorescence Titration Experiments

The fluorescence emission spectrum of 3AC upon excitation at 280 nm is a suitable indicator of the interaction with heparin-like GAGs.^38^ Thus, the titration of 3AC with fond in Bis-Tris buffer at pH 7.5 produced a perturbation of the corresponding intensity of the fluorene emission band (Figure 5A), presenting two clearly differentiated phases. At a low fond/3AC ratio, the fluorescence emission intensity decreases most likely due to the proximity of chromophores when several molecules of 3AC are closely bound to one molecule of fond. After reaching a fond/3AC molar ratio of ∼0.6, the emission band starts to recover as 3AC molecules are redistributed to equimolecular complexes with fond. Considering the disaccharide repeating unit of heparin as the main binding epitope of our ligands and the electrostatic charge complementarity, we can hypothesize the formation of fond/3AC supramolecular complexes with both 1:1 and 1:2 stoichiometries as the simplest binding model satisfying the two-phase behavior (Figure 5B). The emission band variation was successfully fitted to the 1:2 fond/3AC equilibria, rendering the formation constants (log β values) for the corresponding complexes (inset in Figure 5A). The stepwise binding constants for the process were found to be *K*_(1:1)_ = 2.05 ± 0.05 × 10^5^ M^–1^ and *K*_(1:2)_ = 1.95 ± 0.19 × 10^4^ M^–1^. This means an apparent overall affinity of 3AC for fond (*K*_D_^app^ ≈ BC_50_° = 4.50 ± 0.09 μM)^42^ lower than for heparin (*K*_D_^app^ ≈ 0.5 μM),^38^ which can be explained by the multivalency effect of the polysaccharide that is also reflected in the lower activity of fond as the AT_III_ activator. Despite the strong aggregation trends of 3FF in water at neutral pH, we managed to estimate a slightly weaker affinity (*K*_D_^app^ ≈ BC_50_° = 9.8 ± 0.3 μM) using similar fluorescence titration experiments with fond (Figure S4).

**Figure 5:**
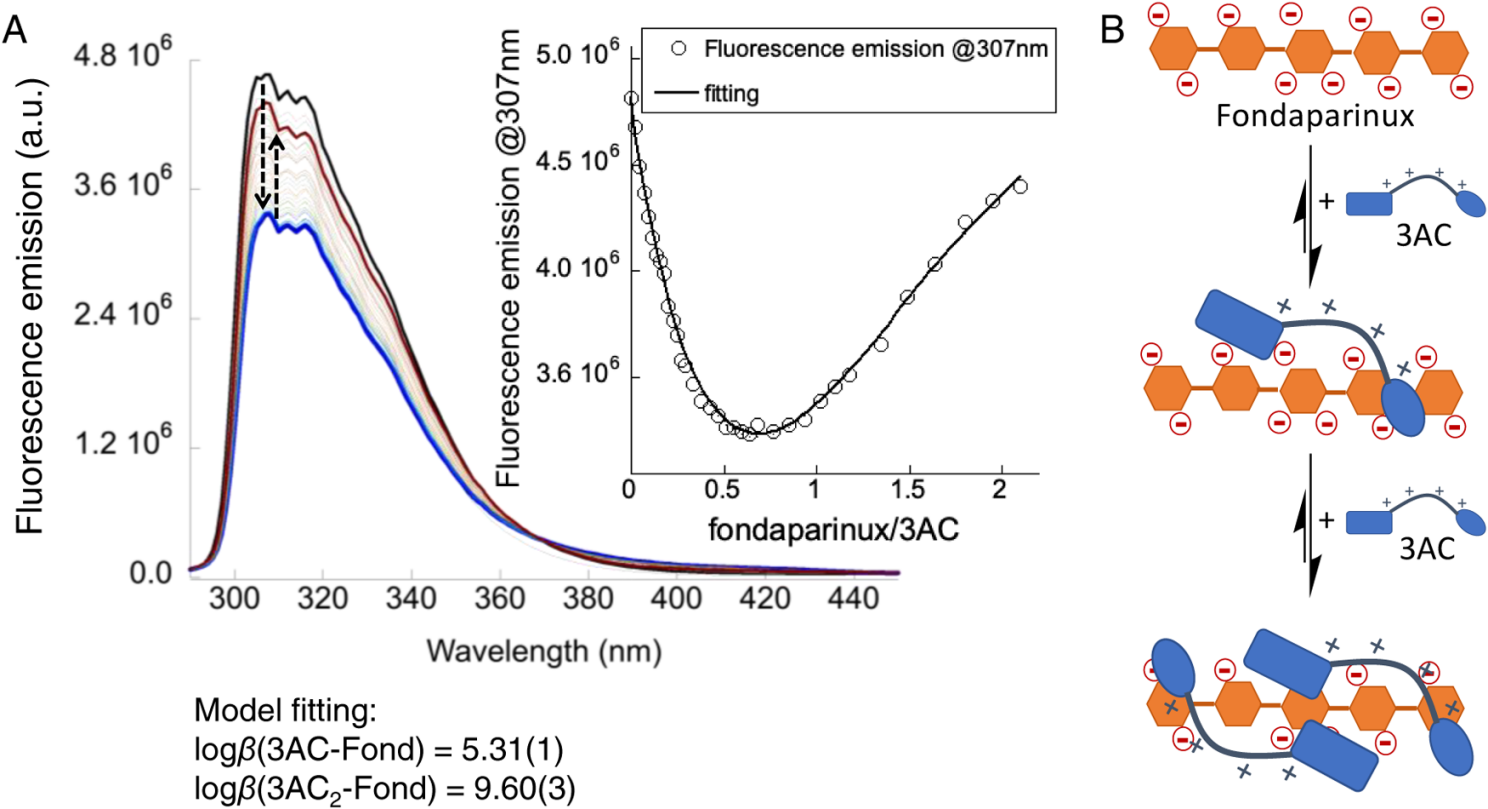
(A) Fluorescence emission spectra of 3AC (10 μM in 1 mM Bis-Tris buffer at pH 7.5, black trace) with increasing concentrations of fond (0–21 μM, colored spectra). Dashed arrows represent the titration sequence (black to blue to red traces). (B) Schematic representation of the proposed binding model. The fluorescence emission spectra were globally fitted (extracted isotherm at 307 nm, inset in Figure 5A) to the 1:2 fond/3AC equilibria, rendering the corresponding *K*_(1:1)_ and *K*_(1:2)_ binding constants (see text).

### Solution-State Structural Analysis by NMR

The [3AC–fond] binding was additionally studied by NMR. Titration of fond with 3AC modifies several ^1^H and ^13^C NMR signals that can be readily monitored by ^1^H and ^1^H–^13^C HSQC NMR experiments (Figures 6 and S5–S7). A close analysis of the corresponding chemical-shift perturbations (Figures 6C and S8) allows determination of the sugar rings to be more affected by the presence of the ligand (see the chemical structure with arbitrary labeling of the rings in Figure 6A). We hypothesized that [fond–3AC] binding occurs by polar interactions (through the ammonium and OH groups of 3AC with sulfate, carboxylate, and OH residues of fond) and nonpolar contacts (by CH–π interactions between aromatic rings of 3AC and pyranose CH bonds of fond). The N,O-disulfonated glucosamine (V) end is the most affected moiety of the molecule, suggesting a closer contact with the fluorene ring of 3AC. The corresponding ^1^H NMR spectra in 90% H_2_O/10% D_2_O at pH 8.2 and 288 K allow detection of the NH-sulfamate protons (Figures 6D and S9) and confirmed the stronger perturbation of ring V. The conformational behavior of fond in the absence and presence of 3AC was additionally studied by acquiring a series of 2D NOESY and 2D ROESY spectra (Figures S10–S13). As a general observation, the intersugar linkage conformation of fond is not appreciably affected by the interaction with 3AC. A key characteristic of a fond structure is the geometry of the 2O-sulfonated iduronic ring (IV). Contrary to the other four sugars that present the conventional ^4^C_1_ chair conformation, sulfated iduronic (IV) shows an equilibrium in solution between a relatively uncommon ^1^C_4_ chair conformation and a ^2^S_0_ skew geometry (Figure 6A).^43,44^ In the solid state, ring IV presents a ^1^C_4_ geometry in free fond crystals^45^ but ^2^S_0_ when bound to the AT_III_ protein.^46,47^ The transition from ^1^C_4_ to ^2^S_0_ approaches the H2 and H5 protons of the six-membered ring. Therefore, the observed NOE between these two protons (H2/H5, purple double-headed arrow in Figure 6A), internally normalized with the NOE between two protons that do not vary very much in their distance during the transition (H4/H5, orange double-headed arrow in Figure 6A), serves as a qualitative probe for the conformational process. In this case, the ratio NOE­(H2/H5)/NOE­(H4/H5) does not significantly change upon fond–3AC binding (Figure S10), suggesting that both conformers should similarly interact with the ligand. Additionally, the ROESY spectra of the fond–3AC complexes showed new intense cross-peaks that could be assigned to intermolecular contacts between the fluorene moiety of 3AC and H2 in N,O-disulfonated glucosamine (V) (Figures S12–S13). However, a relatively important signal overlapping precluded unambiguous discernibility of intra/intermolecular NOE and ROE cross-peaks, making this assignment tentative. In any case, the observation of more intense NOEs within the complex can be related to a less flexible structure with slower tumbling in solution.

**Figure 6:**
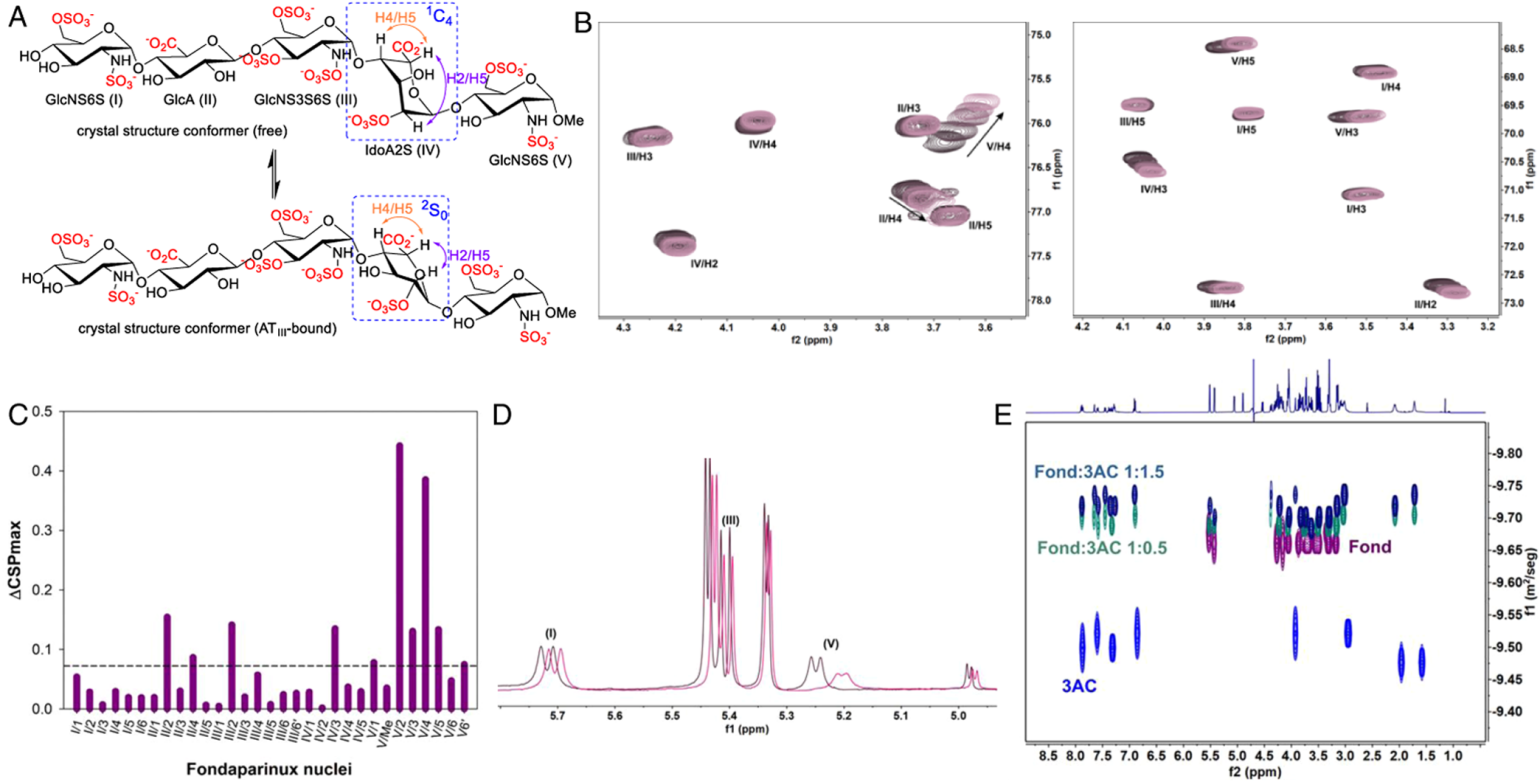
(A) Proposed conformational equilibrium of the fond IV-ring. Key NOE effects are depicted with colored double-headed arrows. (B) Selected regions of superposed ^1^H ^13^C HSQC NMR spectra of 1 mM fondaparinux (in 100% D_2_O, pD 7.0, 295 K) after addition of increasing amounts of 3AC (from 0 to 2.5 mM 3AC, direction indicated with the arrows). (C) Chemical-shift perturbation (CSP) of 1.0 mM fond in the ^1^H and ^13^C dimensions of HSQC spectra after the addition of 2.5 mM 3AC. (D) Detail of the sulfamate NMR (90% H_2_O/10% D_2_O at pH 8.2 and 288 K) proton signals of fond alone (dark pink) and with 1 mM 3AC (light pink). (E) Superposition of DOSY NMR spectra of fond (purple), 3AC (blue), and different proportions of fond/3AC mixtures (cyan or dark blue).

The formation of the fond–3AC complexes in solution was further confirmed by Diffusion Ordered SpectroscopY (DOSY). The DOSY spectra of different mixtures of fond and 3AC showed signals from both partners diffusing at the same rate as part of the same supramolecular entity (Figures 6E and S14). Moreover, the diffusion coefficients of these putative complexes (Table S1) are in all cases lower than those of both fond and especially 3AC alone, implying a larger apparent size in solution. Overall, the NMR data confirm the strong binding of 3AC to fond in water at neutral pH, in good agreement with fluorescence titration results.

Similar NMR experiments performed with 3FF were complicated by the higher tendency of this ligand to aggregate at the concentrations needed for NMR and at neutral pH.^38^ Despite that, both ^1^H and ^1^H–^13^C HSQC titrations of fond with 3FF confirmed the efficient interaction in solution (Figures S15 and S16). Qualitative comparison of the chemical-shift perturbations of fond induced by 3FF (Figure S17) and 3AC (Figures 6C and S8) suggests a slightly weaker binding of 3FF, in good agreement with the fluorescence titration results. In this case, despite DOSY experiments (Figures S18 and S19) being especially troublesome due to the unavoidable presence of 3FF aggregates, the fond–3FF complexes showed a similar diffusion coefficient to that of fond–3AC, in line with their expected comparable sizes in solution. Thus, although 3FF competitively aggregates in water, the NMR data clearly confirmed the formation of the corresponding fond–3FF supramolecular complexes.

### Molecular Dynamics Study of the Complexes

In order to assess the above observations, molecular dynamics simulations of fond, either alone or in complex with one or two molecules of 3AC or 3FF, were conducted. As noted, different fond structures exist, which primarily differ in the conformation of the sulfated iduronic (IV) ring, which can adopt either a ^1^C_4_ or ^2^S_O_ conformation. Accordingly, two representative fondaparinux structures, which will be denoted ^1^C_4_-fond and ^2^S_O_-fond from now on, were selected for this study (see Computational Methods).

On the other hand, various force fields have been employed in carbohydrate simulations.^48^ GLYCAM06 ^49^ and CHARMM36 ^50^ were recently used to explore the conformational space of fondaparinux, concluding that GLYCAM06 aligns more closely with experimental NMR results.^51^ However, in our simulations (250 ns at 300 K) with GLYCAM06, we observed that the ^2^S_O_ fondaparinux conformation was under-represented, regardless of whether ^1^C_4_-fond or ^2^S_O_-fond was used as the starting structure (Figure S22). Specifically, the simulation starting with ^1^C_4_-fond showed that the ^2^S_O_ conformation was only briefly sampled (208–213 ns), while the one beginning with ^2^S_O_-fond showed a fast transition (within the first 3 ns) of the IdoA ring from ^2^S_O_ to ^1^C_4_, revisiting the ^2^S_O_ conformation only once (227–237 ns). We then investigated whether the OPLS4 force field^52^ might better represent the ^2^S_O_ conformation of the IdoA ring and found that, in contrast to GLYCAM06, this force field appears to favor the ^2^S_O_ conformation, with some contribution from the ^2,5^B conformation, over the ^1^C_4_ alternative (Figure S22). Indeed, simulation with ^1^C_4_-fond showed that the initial ^1^C_4_ IdoA conformation transitioned to ^2^S_O_ within the initial 8 ns, only briefly reverting to ^1^C_4_ between 107 and 111 ns. Instead, starting with ^2^S_O_-fond revealed the IdoA ring remaining predominantly in the ^2^S_O_ form throughout.

With these findings in mind, we decided to conduct simulations (250 ns at 300 K) of the fond–ligand complexes using both force fields to adequately investigate both conformational forms of fondaparinux in the presence of our ligands. Specifically, we performed simulations starting with either ^1^C_4_-fond or ^2^S_O_-fond and one or two molecules of 3AC or 3FF. For comparison, we also conducted analogous simulations with ciraparantag (cir). Finally, to estimate the relative affinity of our most potent ligand, 3AC, compared to cir, we performed competition simulations with fond and two molecules of each ligand. The simulated systems are summarized in Table 1.

**Table 1:** Summary of Simulation Systems Studied Using Both OPLS4 and GLYCAM06 as Force Fields for the Carbohydrate

fond	fond–ligand			competition
^2^S_o_-fond	^2^S_O_-3AC	^2^S_O_-3FF	^2^S_O_-cir	^2^S_O_-3AC_2_-cir_2_
	^2^S_O_-3AC_2_	^2^S_O_-3FF_2_	^2^S_O_-cir_2_	
^1^C_4_-fond	^1^C_4_-3AC	^1^C_4_-3FF	^1^C_4_- cir	^1^C_4_-3AC_2_-cir_2_
	^1^C_4_-3AC_2_	^1^C_4_-3FF_2_	^1^C_4_- cir_2_	

Regarding the influence of the ligands on the conformational state of fondaparinux, the results indicate that the presence of one or two molecules of each ligand does not significantly alter the conformational behavior observed for free fondaparinux (Figures S23–S34). This suggests that the interactions between fondaparinux and the ligands do not substantially shift the equilibrium between the ^1^C_4_ and ^2^S_O_ conformations, in agreement with the NMR experimental results.

The radius of gyration (RoG) measures the distribution of atoms in a molecular structure relative to its center of mass, providing an estimate of the structure’s compactness. In the simulations of fondaparinux alone, the RoG remained consistently at around 7.9 Å (Figure S35). We also calculated the RoG for simulations of fondaparinux in the presence of ligands, considering either the fond molecule alone or in combination with one or two ligand molecules for the calculation. The expectation was that if the ligand molecules were tightly bound (i.e., making numerous contacts with the fond molecule), the fond–ligand RoG trace would closely match that of fond alone. Conversely, if the ligands were loosely bound or completely unbound, both RoG traces would diverge significantly. For the simulations with a single molecule of 3AC or 3FF, the results (Figure S35) indicate that both ligands bind tightly to the fondaparinux molecule. However, in simulations with two molecules of each ligand, some divergence between the RoG traces is observed, particularly in simulations using the OPLS4 force field (Figure S35), which led to a slight increase in the average RoG value of the complex relative to that of fond alone, aligning with the increase in the hydrodynamic radius observed in the DOSY experiments. These effects were even more pronounced in simulations with one or two ciraparantag molecules. The increased RoG values for this complex suggest that fitting two cir molecules tightly bound to a single fondaparinux molecule is particularly challenging, likely due to the steric hindrance or competition for binding sites, aggravated by the larger size of the ciraparantag ligand.

Inspection of simulation snapshots for fondaparinux in the presence of 3AC or 3FF reveals that binding of these ligands to fond is primarily driven by hydrogen bonding, ionic interactions between charged groups, and CH−π sugar–aromatic interactions (Figure 7A,B,D,E). These interactions are consistent with those previously observed for the same ligands binding to a heparin model,^38^ and they occur irrespective of the force field (OPLS4 or GLYCAM06) or the fondaparinux model (^1^C_4_-fond or ^2^S_O_-fond) used. The corresponding CH−π sugar–aromatic interactions with the N,O-disulfonated glucosamine (V) ring are especially noteworthy (see representative snapshots in Figure 7A,B,D,E), which are consistent with the chemical-shift perturbations observed experimentally (Figure 6C). In contrast, fond–cir complexes are stabilized exclusively by hydrogen bonding and ionic interactions (Figure 7C,F). Consequently, these results suggest that the predominance of one or another conformation, i.e., ^1^C_4_ or ^2^S_O_, for the fond IdoA ring does not hinder the binding of any of the studied ligands to the carbohydrate, as experimentally observed by NMR. This is likely attributable to the high flexibility and extensive functionalization of both fondaparinux and the ligands, enabling them to adopt conformations that maximize stabilizing interactions across different molecular regions (Figures S25, S26, S29, S30, S33, and S34).

**Figure 7:**
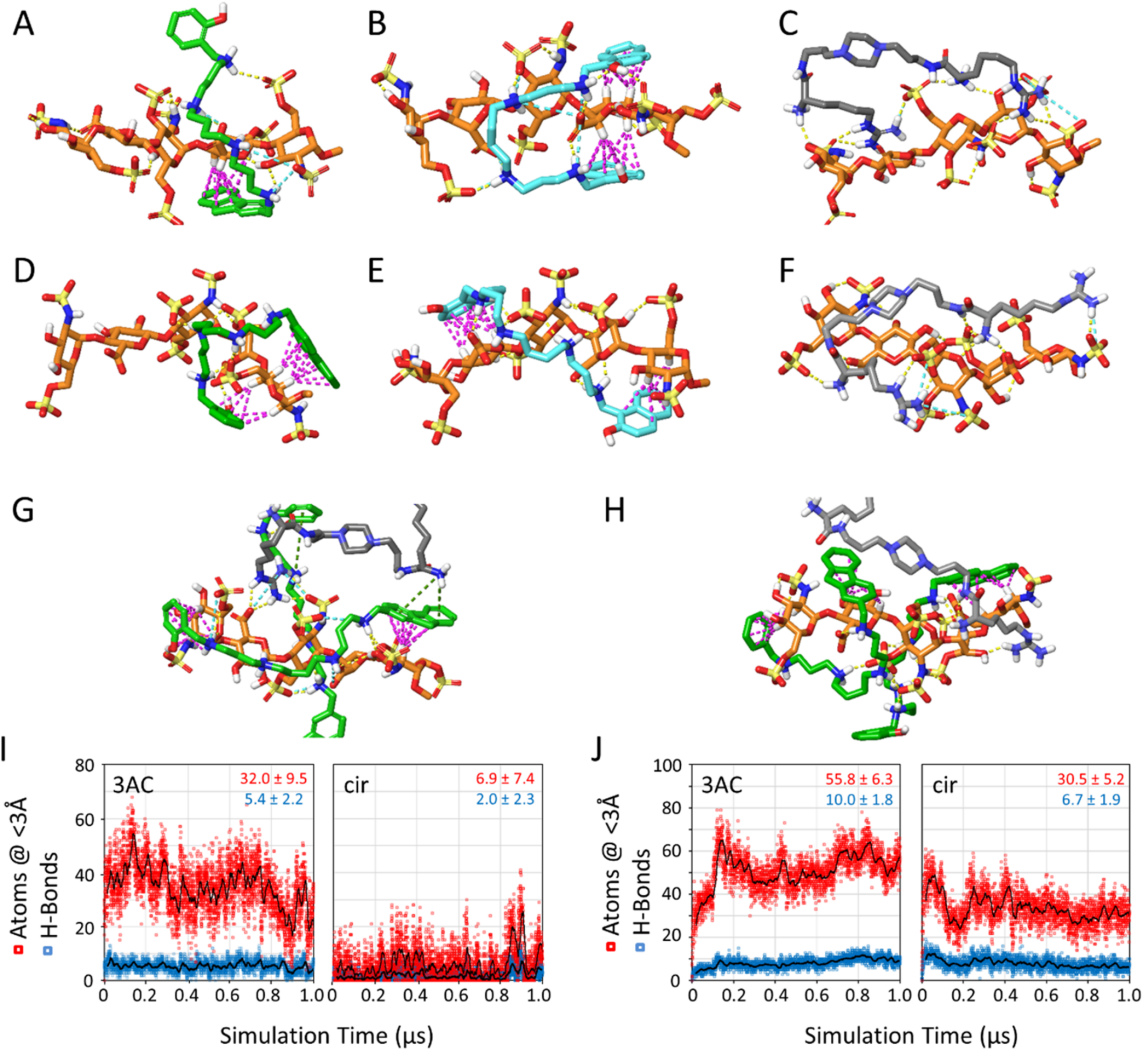
Representative snapshots of the molecular dynamics simulations performed using either OPLS4 or GLYCAM06 as the carbohydrate force field: (A) ^2^S_O_-3AC (OPLS4), (B) ^2^S_O_-3FF (OPLS4), (C) ^2^S_O_-cir (OPLS4), (D) ^1^C_4_-3AC (GLYCAM06), (E) ^1^C_4_-3FF (GLYCAM06), (F) ^1^C_4_- cir (GLYCAM06), (G) ^2^S_O_-3AC_2_-cir_2_ (OPLS4), and (H) ^1^C_4_-3AC_2_-cir_2_ (GLYCAM06). Fond is represented as sticks with orange C-atoms, while 3AC, 3FF, and cir are represented as sticks with green, cyan, or gray C-atoms, respectively. Interactions between molecules are shown as yellow (H-bonding), cyan (salt bridges), violet (CH–π), or green (π-cation) dashed lines. (I and J) Graphs derived from the competition simulations with (I) ^2^S_O_-3AC_2_-cir_2_ (OPLS4) and (J) ^1^C_4_-3AC_2_-cir_2_ (GLYCAM06), representing the total number of hydrogen bonds between ligands and fond (blue squares), and the total number of ligand atoms within a distance of 3 Å from fond (red squares) vs simulation time. The black lines represent smoothed trend lines. Average values for the last 500 ns are shown with respective colors for each graph.

Concerning the competition simulations between 3AC and cir (1 μs at 300 K, repeated twice to improve sampling), the results reveal a larger number of hydrogen bonds and close contacts between 3AC and fond compared with cir and fond (Figures 7I,J and S36). Analysis of simulation snapshots further indicates that during significant portions of the simulations, two 3AC molecules, but only one cir molecule, are simultaneously bound to the fond molecule (Figures 7G,H, S37 and S38), suggesting a stronger binding for 3AC than for cir, in line with our *in vitro* and *ex vivo* experimental results. It must be pointed out that both cir and 3AC bore the same charged states during the competition simulations (Figure S39). Accordingly, a similar electrostatic contribution to the binding is expected for both 3AC and cir, although the smaller size of 3AC would lead to a slightly higher charge density. However, close inspection of the representative snapshots (Figure 7G,H) revealed the importance of the interactions between the CH groups of the pyranose rings and the aromatic moieties of 3AC (violet CH−π contacts in Figure 7). This binding motive is also present in nature for the sugar recognition^53^ and represents a nice validation of our chemical design.

## Conclusions

Herein, we report two spermine derivatives as efficient reversal agents of the synthetic anticoagulant fondaparinux. They show higher *in vitro* enzymatic activity than other heparinoid antidotes like protamine (which is inefficient against fondaparinux) or ciraparantag (a small molecule currently in clinical trials as a universal anticoagulant antidote). Their efficiency and higher potency were further demonstrated in a more relevant environment like freshly extracted mouse blood. Binding studies using experimental techniques (fluorescence and NMR) in addition to molecular modeling show the formation of strong noncovalent complexes of these molecules with fondaparinux as the mechanism of action. Besides, the smaller size of both anticoagulant and antidotes allowed a detailed characterization of the supramolecular structures, leading to a better understanding of the key structural parameters for the binding and concomitant activity. Taking into account their promising activity against heparinoids of broad range of molecular weights,^38^ we envision their high potential as unique universal antidotes for all types of heparin-like anticoagulants, with no molecular-weight restrictions from large UFH to the smallest derivative fondaparinux. Provided that further preclinical studies are done, being small molecules with a relatively simple preparation and chemical structure, they could be further developed as an alternative to the current heparin antidote, protamine, which has some safety drawbacks, as well as low efficacy with LMWH and, specifically, fondaparinux.

## Experimental Section

### Materials and Methods

Fondaparinux was purchased from Sigma-Aldrich. LMWH was purchased from Iduron. Compounds 3AC, 3FF, 3AA, and 3BB were synthesized as previously described,^37,38^ while ciraparantag was prepared by conventional solution-phase peptide synthesis from commercially available precursors.^30^ All compounds were >95% pure, as determined by HPLC analysis. All procedures were approved by the Animal Care and Use Committee of CID-CSIC (OH code: 1032/2020) and conducted in accordance with the institutional guidelines under a license from the local government (agreement number: 11120).

### Blood Coagulation Factor In Vitro Enzymatic Assays

Recombinant antithrombin III and coagulation factor X_a_ were obtained from two different kits, Berichrom Heparin test (SIEMENS) and Kinetichrome Anti-X_a_ Heparin kit (Iduron). Methods for both kits are similar, but not identical. Differences will be spotted by method A (SIEMENS) and method B (Iduron). Following the manufacturer’s instructions, stock solutions were prepared as follows: human antithrombin III (1 IU/mL) and factor X_a_ reagent (0.4 μg/mL, human plasma fraction with additives Tris, sodium chloride, and EDTA). Chromogenic substrate specific for factor X_a_ was dissolved at 4 mM concentration (A: Z-d-Leu-Gly-Arg-ANBA-methyl amide, and B: Z-d-Arg-Gly-Arg-pNA). Additionally, either 4-nitroaniline (A) or *N*-Boc-4-nitroaniline (B) was added to the substrate solution at a concentration of 1.6 mM and used as an internal standard to quantify the hydrolysis of the chromogenic substrate. All compounds were dissolved in Milli-Q water at the desired stock concentrations prior to start of the assays and kept at 4 °C. Various concentrations of the ligand, fondaparinux (up to 15 μM) or LMWH (0.1 to 10 μM), human antithrombin III solution (2 μL), and factor Xa reagent solution (35 μL) were consecutively added to an Eppendorf vial and brought to a total volume of 145 μL. Then, 20 μL of reagent substrate solution was added to start the experiment, and the mixture was vigorously shaken at 25 °C. 20 μL aliquots were taken at 5/10/15/20/30/50/80/120 minutes, diluted with 40 μL of acetic acid (20% v/v), and injected into the analytical HPLC system. The gradient ranged from 5% of ACN (0.1% formic acid) in water (0.1% formic acid) to 100% of ACN in 24 min using a 15 × 4.6 mm KROMAPHASE C_18_ 5.0 μm column. In A: the retention time of cleaved chromophore was 9.1 min, retention time of chromogenic substrate was 13.9 min, and retention time of 4-nitroaniline was 15.4 min. In B: the retention time of chromogenic substrate was 11.0 min, retention time of cleaved chromophore was 15.2 min, and retention time of *N*-Boc-4-nitroaniline was 21.7 min. FX_a_/AT_III_ activity was represented as the percent of hydrolysis, which was calculated from the normalized area of the cleaved peptide at 405 nm at each corresponding time point. The experiment carried out in the absence of heparinoid was considered as the maximum of activity, while the experiment with heparinoid and no ligand was considered as negative control (maximum inhibition of FX_a_ by heparinoid). The concentration of heparinoid was selected for the measurement to render significant inhibition within experimental time while allowing the reaction to proceed.

### *Ex Vivo* Blood Coagulation Assays

This naked-eye coagulation test is an adaptation of the whole blood clotting time assay.^26,54^ Freshly collected mouse blood was directly used without further treatment. 300 μL aliquots were dispensed in Eppendorf tubes, to which fondaparinux (100 μM), and/or potential antidotes (3AC, 3FF, ciraparantag, or protamine, 200 μM) were added, and a picture was taken after 5 min. Clot formation was verified by inverting the Eppendorf vials.

### Scanning Electron Microscopy

Fresh blood from mice was collected in 300 μL aliquots, to which fondaparinux (100 μM), fondaparinux and 3AC (200 μM), 3FF (200 μM), or 3AC/3FF antidotes alone were added. After gently shaking the blood samples for 10 min, 30 μL aliquot of each was spread onto a glass coverslip. Samples were then fixed by adding 2.5% glutaraldehyde to 100 mM sodium cacodylate buffer at pH 7.0 (overnight at 4 °C), and sequentially washed with (1) sodium cacodylate buffer (1 × 2 min), (2) Milli-Q water (2 × 2 min), (3) 25% EtOH in H_2_O (1 × 5 min), (4) 50% EtOH in H_2_O (1 × 5 min), (5) 75% EtOH in H_2_O (1 × 5 min), (6) 95% EtOH in H_2_O (3 × 5 min), and (7) 100% EtOH (3 × 10 min). Finally, the samples were dried and kept at 4 °C. NOTE: It is handier to place the coverslips in 12-well plates as operations become easier to manage. The samples were mounted on double-coated carbon conductive tape, and pictures were taken with a Hitachi TM-4000Plus II SEM microscope (1000 or 2000 magnifications). Pictures are shown with no further postprocessing treatment.

### Cell Growth

A549 and HeLa cells were maintained in DMEM (4500 mg/mL glucose, Sigma) containing 10% fetal calf serum (FCS), 2 mM glutamine, 50 U/mL penicillin, and 0.05 g/mL streptomycin at 37 °C under a 5% CO_2_ atmosphere. HMEC-1 and fibroblast cells were maintained in MCDB 131 medium (GIBCO) containing 10% fetal calf serum (FCS), 2 mM glutamine, 50 U/mL penicillin, and 0.05 g/mL streptomycin at 37 °C under a 5% CO_2_ atmosphere.

### MTT Assay

The viability of A549 and HeLa cells was tested using the 3-(4,5-dimethylthiazol-2-yl)-2,5-diphenyltetrazolium bromide (MTT) assay. In it, exponentially growing cells were detached from the culture flasks using a trypsin–0.25% EDTA solution, and the cell suspension was seeded onto a 96-well plate (Nunclon) at a concentration of 9k cells/well. The toxicity of 3AC and 3FF was tested as follows: 24 h after seeding, culture medium was discarded and replaced by compound solution in PBS that was diluted with cell culture medium to the final concentration: [3××] = 10 to 100 μM. MTT (0.5 mg/mL) was added. After 2 h of incubation with MTT, the medium was discarded by aspiration, DMSO was added to dissolve formazan, and dark-blue colored crystals were observed in the wells. Absorbance was measured at 570 nm using a spectrophotometric BioTek Synergy 2 Microplate Reader (Agilent), 30 min after the addition of DMSO. Cell viability is expressed as an absorbance percent ratio of cells treated with compound to untreated cells, which were used as a control. The results are the average from three independent experiments.

### Resazurin Assay

The viability of HMEC-1 and fibroblast cells was tested using the resazurin assay. In it, exponentially growing cells were detached from the culture flasks using a trypsin–0.25% EDTA solution, and the cell suspension was seeded onto a 96-well plate (Nunclon) at a concentration of 9k cells/well. Toxicity of 3AC and 3FF was tested as follows: 72 h after seeding, culture medium was discarded and replaced by compound solution in PBS that was diluted with cell culture medium to the final concentration: [3××] = 10 to 100 μM. Resazurin (0.1 mg/mL) was added. After 2 h of incubation, fluorescence was measured (λ_ex_ = 560 nm // λ_em_ = 590 nm) using a spectrophotometric BioTek Synergy 2 Microplate Reader (Agilent). Cell viability is expressed as an emission percent ratio of cells treated with the compound to untreated cells, which were used as a control. The results are the average from three independent experiments. In experiments with fondaparinux, the cells were incubated for 2 h with [fondaparinux] = 100 μM and [3××] = 50/100/200 μM following the same procedure for the rest of the protocol.

### Hemolysis Assays

Mouse blood (4 mL) was collected in a sodium citrate tube and immediately centrifuged at 1700*g* for 5 min. The supernatant was removed, and the erythrocytes were washed by adding 2 mL of PBS. It was centrifuged again at 1700*g* for 5 min. The washing step was repeated twice more. The supernatant was finally removed, and the erythrocytes were diluted in PBS until obtaining a roughly 4–5% suspension. 50 μL of erythrocyte solution was mixed with 50 μL of test compound (3×× at the desired concentration). As a negative control, 50 μL of PBS was used, while 10% Triton X-100 was used as a positive control. The samples were incubated at 37 °C for 60 min. After that, they were centrifuged (1700*g* for 5 min), and the supernatant was transferred to a 96-well plate. Absorbance was measured at 405 nm. The experiment contained three technical replicates. The results are the average from three independent experiments.

### Fluorescence Titration Experiments

Fluorescence emission and excitation spectra were collected on a Photon Technology International Instrument, Fluorescence Master Systems, using Felix32 software and cuvettes with 10 mm path length. Stock solutions of 3AC (10 μM) and fondaparinux (0.23 mM, also containing 10 μM 3AC) were prepared in 1 mM Bis-Tris buffer at pH 7.5. Then, 2 mL of binder solution was placed on a quartz cell, and the emission fluorescence spectrum was measured upon excitation at 280 nm. Then, small volumes of fond stock solution were added to the cell, and the fluorescence spectra were acquired after each addition. The titration experiments were fitted using HyperSpec 2008 software,^55,56^ which allows a nonlinear global fitting of the full emission spectra to a binding mode as defined by the user.

### Nuclear Magnetic Resonance Experiments

One- and two-dimensional (1D and 2D) NMR experiments were performed at 298 K on a 500 MHz Bruker AVANCE III HD instrument equipped with a z-gradient (65.7 G cm^–1^) inverse TCI-cryoprobe. Samples were dissolved in 5 mM Tris-d11 buffer with 50 mM NaCl (in D_2_O, pH 7.5, uncorrected pH meter reading). Bruker TopSpin 3.5pl6 standard pulse sequences were used for 1D and 2D experiments. For DOSY experiments, the stebpgp1s19 pulse sequence with WATERGATE 3919 for water suppression and one spoil gradient were used.

### Computational Methods

Most modeling and visualization tasks were carried out with the package Schrödinger Suite 2024,^57^ through its graphical interface Maestro.^58^ The fondaparinux coordinates were extracted from CCDC 1553209 ^45^ and PDB 3EVJ.^59^ These structures differ in the conformation of the iduronic acid (IdoA) ring: in CCDC 1553209, the ring adopts a ^1^C_4_ conformation, while in PDB entry 3EVJ, it adopts a ^2^S_o_ conformation. Accordingly, the first structure is termed ^1^C_4_-fond and the second as ^2^S_o_-fond. The fondaparinux molecules were modeled with all sulfate and carboxylate groups ionized (total charge: −10). Ligands 3AC, 3FF, and ciraparantag were built within Maestro, in an extended conformation and with four protonated amino groups, as predicted by Epik^60,61^ at pH 7.4 (Figure S39). ^1^C_4_-fond and ^2^S_o_-fond were simulated both alone or together with one or two molecules of each ligand, randomly placed, and with all of its atoms at least 5 Å away from the fond molecule. Additionally, competition simulations were performed with one fondaparinux molecule and two molecules each of 3AC and cir.

### Simulations with OPLS4

Each simulation system was submitted to molecular dynamics with the program Desmond 7.8,^62,63^ as included in the Schrödinger Suite 2024,^57^ using the OPLS4 force field^52^ to parametrize the ligands and the fondaparinux molecule. Each system was set up using the System Builder of the Maestro-Desmond interface.^64^ Solutes were placed in the center of a truncated octahedral periodic box extended 10 Å away from any solute atom, Na^+^ or Cl^–^ ions were added as necessary to achieve neutrality, and systems were solvated with TIP3P water molecules. The systems were subjected to the default relaxation protocol consisting of 100 ps Brownian dynamics at 10 K in the NVT ensemble, with 50 kcal mol^–1^ Å^–2^ restraints on solute heavy atoms and small timesteps (1 fs); 12 ps molecular dynamics at 10 K (Langevin thermostat) in the NVT ensemble, keeping the restraints and small timesteps as before; 12 ps MD at 10 K and 1.0 atm (Langevin thermostat and barostat) in the NPT ensemble, keeping the restraints except with 2 fs timesteps; 12 ps MD keeping the previous conditions except for the temperature of 300 K; 24 ps MD keeping the previous conditions but without restraints. Production MD simulations for the systems composed of fond alone or with one or two molecules of each ligand were run for 250 ns, while the competition simulations were run for 1 μs twice, starting in each case with different locations for the ligand molecules. All simulations were performed under the same conditions (PBC, NPT ensemble, 300 K and 1.0 bar, 2 fs time step) using the Nosé–Hoover thermostat method^65^ with a relaxation time of 1.0 ps and the Martyna–Tobias–Klein barostat method^66^ with isotropic coupling and a relaxation time of 2 ps. Integration was carried out with the RESPA integrator^67^ using timesteps of 2.0, 2.0, and 6.0 fs for the bonded, short-range, and long-range interactions, respectively. A cutoff of 9.0 Å was applied to van der Waals and short-range electrostatic interactions, while long-range electrostatic interactions were computed using the smooth particle mesh Ewald method with a 10^–9^ Ewald tolerance.^68,69^ Bond lengths to hydrogen atoms were constrained using the SHAKE algorithm.^70^ Coordinates were saved every 50 ps. The Desmond-Maestro interface was used to analyze the simulation results. The variation in the Cremer–Pople parameters for the fondaparinux rings during the simulation was assessed by saving 1000 equally spaced frames from each simulation and analyzing them using custom scripts specifically designed for this purpose.^71^

### Simulations with GLYCAM06

The simulation systems were also submitted to molecular dynamics with AMBER 2022 ^72^ with GPU acceleration.^73–75^ The GLYCAM_06j-1 force field^49^ with additional parameters for glycosaminoglycans^76^ was used to parametrize the fond molecule, adjusting the partial charges on the sulfur-bound O- and N-atoms according to the GLYCAM procedure for charge development. GAFF2 parameters^77^ and atomic RESP charges^78^ were used for the ligands. To that end, ligands 3AC, 3FF, and cir were optimized, and their electrostatic potential (ESP) was calculated at the HF/6-31G* level with Gaussian09.^79^ Partial atomic charges were then calculated by RESP fitting using Antechamber.^80^ tLEAP was used to assign parameters to the structures, as well as to solvate them with a truncated octahedral TIP3P box that was extended 12 Å away from any solute atom, adding enough chloride or sodium ions to reach neutrality. The solvated systems were minimized with 10,000 steps of steepest descent plus 10,000 steps of conjugate gradient. The systems were then thermalized and equilibrated in three successive MD steps: (1) 50,000 MD steps from 0 to 300 K, 2 fs per step, periodic boundary conditions (PBC) on the NVT ensemble, Lagevin dynamics for temperature control and a thermostat collision frequency of 1.0 ps^–1^, a cutoff of 10 Å for short-range interactions, and particle mesh Ewald (PME) method for long-range interactions, constraining bonds to hydrogens with SHAKE; (2) 50,000 MD steps at 300 K, with rest of the conditions as before; (3) 10^7^ MD steps (i.e., 20 ns), with conditions as before except for PBC on the NPT ensemble, at 1 atm with isotropic position scaling and Monte Carlo barostat control. Production MD simulations (250 ns or 1 μs, see above) were run using the same parameters as the previous equilibration step, saving snapshots and energy information every 50 ps. Trajectory analysis and calculation of Cremer–Pople parameters were performed with CPPTRAJ.^81^ Visualization was performed with VMD-1.9.4a57.^82^

## Supplementary Material



## Notes

HPLC traces of synthesized compounds; additional *in vitro* experiments; fluorescence titration of 3FF; additional NMR spectra and data; and complementary molecular modeling results (PDF)
