# Supplementary material for: Efficient Small-Molecule Reversal Agents for Anticoagulant Fondaparinux
Source: ACS Pharmacol Transl Sci. 2025 Apr 29;8(5):1333–46. doi: 10.1021/acsptsci.4c00747 (PMC12070314; doi:10.1021/acsptsci.4c00747)
Supplement: Supplementary file 1 [file pt4c00747_si_001.pdf]

Efficient Small-Molecule Reversal Agents for Anticoagulant Fondaparinux

Daniel Carbajo,[a] Yolanda Pérez,[b] Gabriela F. Castelo,[a] Eva Prats,[c] Jordi Bujons,[a] and Ignacio Alfonso[a,]*

[a]*Department of Biological Chemistry, Institute for Advanced Chemistry of Catalonia, IQAC-CSIC, Jordi Girona 18-26, 08034, Barcelona, Spain.*

[b]*NMR Facility, Institute for Advanced Chemistry of Catalonia, IQAC-CSIC, Jordi Girona 18-26, 08034, Barcelona, Spain.*

[c]*Animal Facility, Research and Development Center (CID-CSIC), Jordi Girona 18-26, 08034, Barcelona, Spain.*

Corresponding author e-mail: ignacio.alfonso@iqac.csic.es

Table of contents:

HPLC traces of the studied molecules S-1

In vitro activity assays (Figures S2-S3) S-2

Fluorescence titration of 3FF with fondaparinux (Figure S4) S-5

NMR studies with 3AC (Figures S5-S14, Table S1) S-6

NMR studies with 3FF (Figures S15-S21) S-17

Molecular modelling results (Figures S22-S39) S-24

3AC

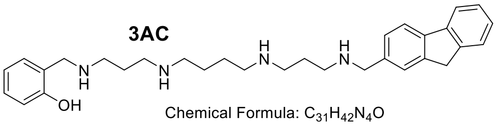

Chemical Formula: $C_{31}H_{42}N_4O$
Molecular Weight: 486.70

HPLC: t_r = 8.7 min, purity at 220 nm >99%

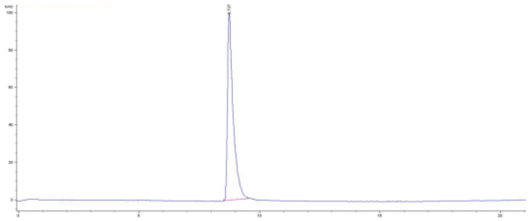

3AA

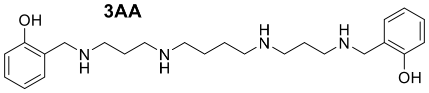

Chemical Formula: $C_{24}H_{38}N_4O_2$
Exact Mass: 414,30
Molecular Weight: 414,59

HPLC: t_r = 9.6 min, purity at 220 nm = 97.3%

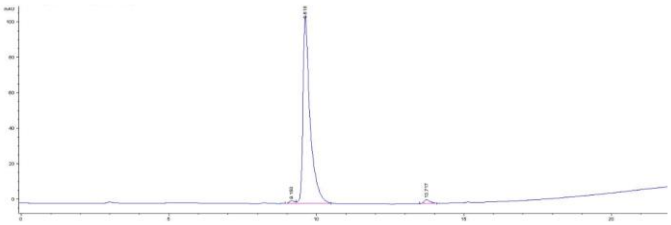

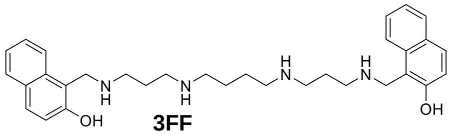

3FF

Chemical Formula: $C_{32}H_{42}N_4O_2$
Exact Mass: 514,33
Molecular Weight: 514,71

HPLC: t_r = 9.4 min, purity at 220 nm = 99%

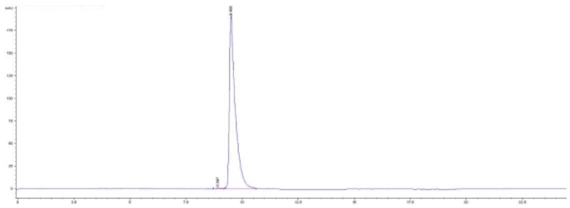

3BB

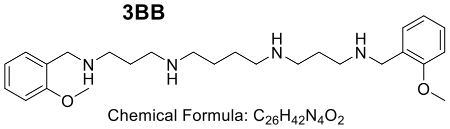

Chemical Formula: $C_{26}H_{42}N_4O_2$
Exact Mass: 442.3308
Molecular Weight: 442.6480

HPLC: t_r = 8.6 min, purity at 220 nm >99%

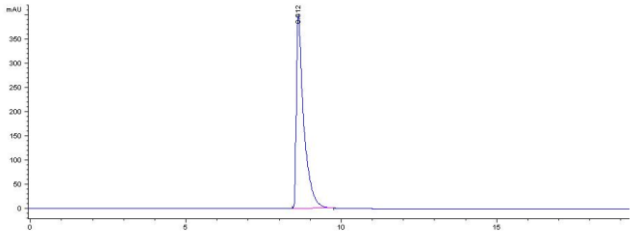

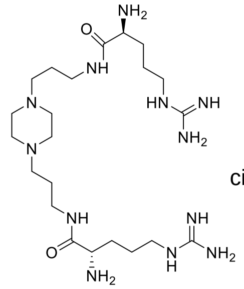

ciraparantag

Chemical Formula: $C_{23}H_{49}N_{11}O_2$
Exact Mass: 511.41

HPLC: t_r = 3.05 min, purity at 220 nm = 96.2%

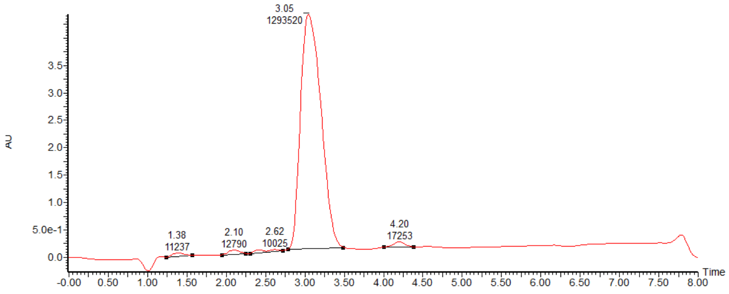

Figure S1. HPLC traces for all the molecules studied in this work.

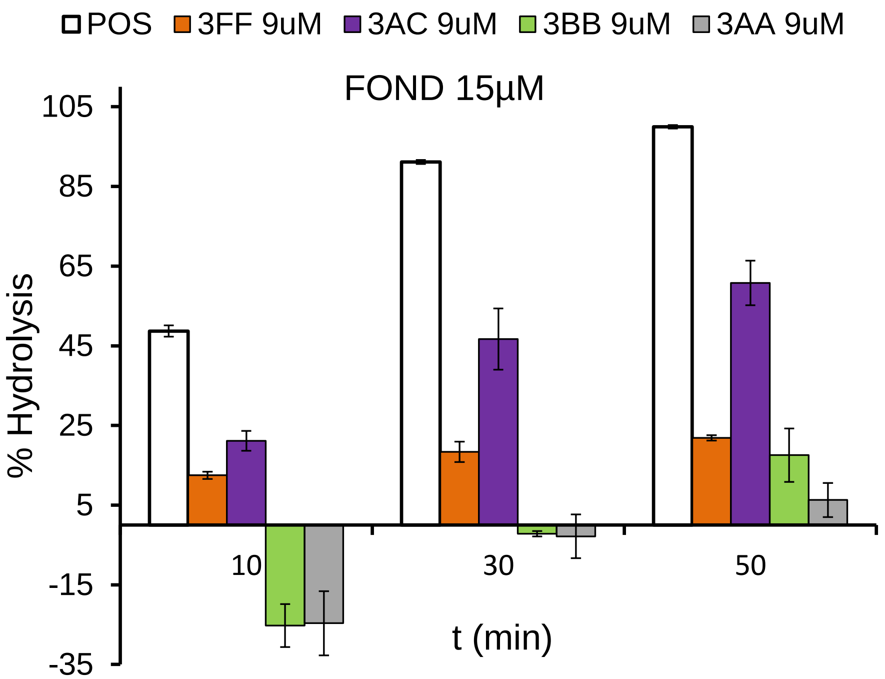

Figure S2. Plot of the percent recovery of peptide substrate hydrolysis ((hydrolysis recovery vs. fond / max. hydrolysis recovery vs. fond) x 100) from samples containing FXa/AT_{III} at different reaction times: positive control with maximal FXa activity (white), fond+3FF (orange), fond+3AC (purple), fond+3BB (green), fond+3AA (grey). In all the cases [fond] = 15 μM and [antidote] = 9 μM. All experiments were carried out at least in triplicate.

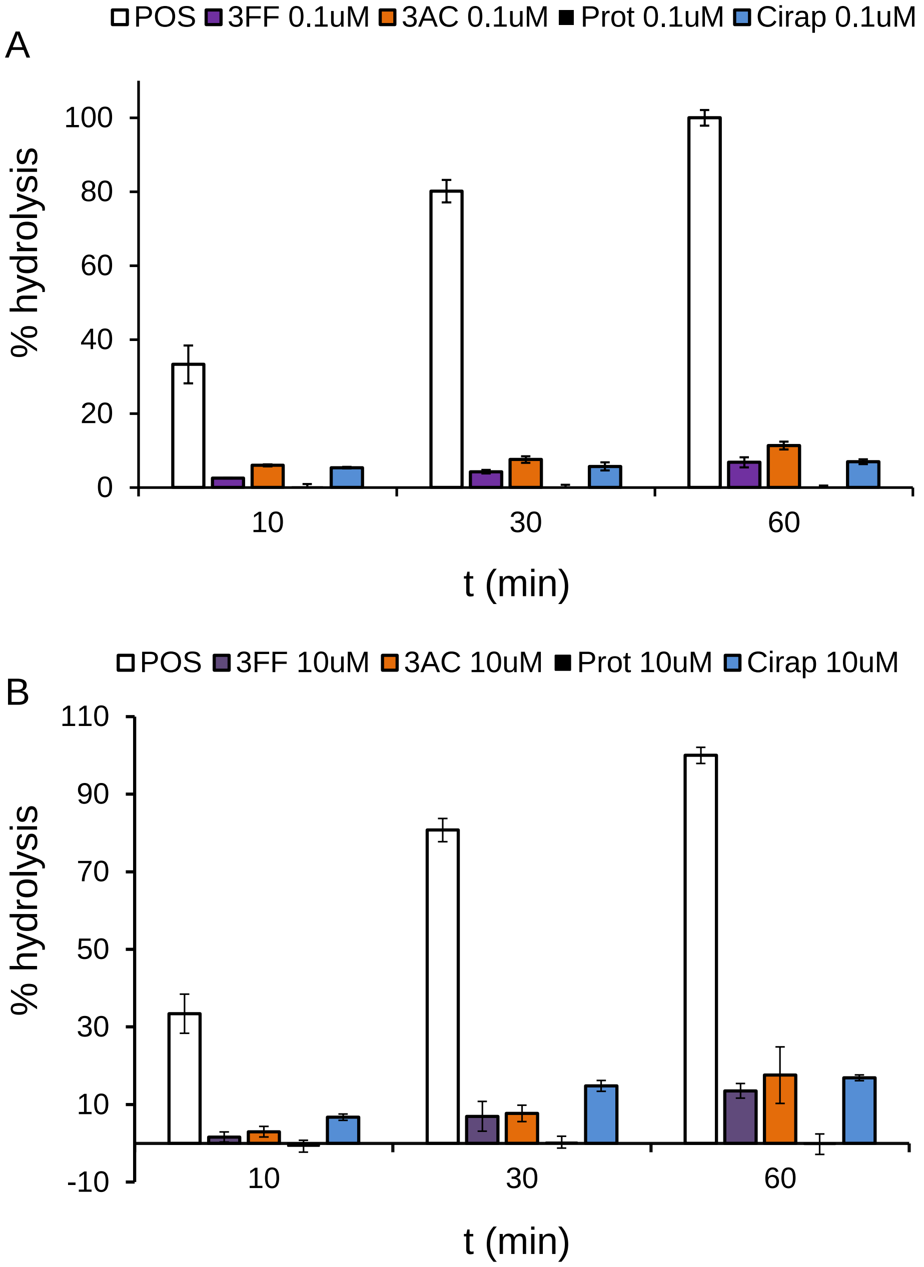

Figure S3. Plot of the percent recovery of peptide substrate hydrolysis ((hydrolysis recovery vs. LMWH / max. hydrolysis recovery vs. LMWH) x 100) from samples containing FXa/AT_{III} at different reaction times: positive control with maximal FXa activity (white), LMWH+3FF (purple), fond+3AC (orange), LMWH+prot (black) and LMWH+cir (blue). (A) [LMWH] = [antidote] = 0.1 μM; (B) [LMWH] = [antidote] = 10 μM. All experiments were carried out at least in triplicate.

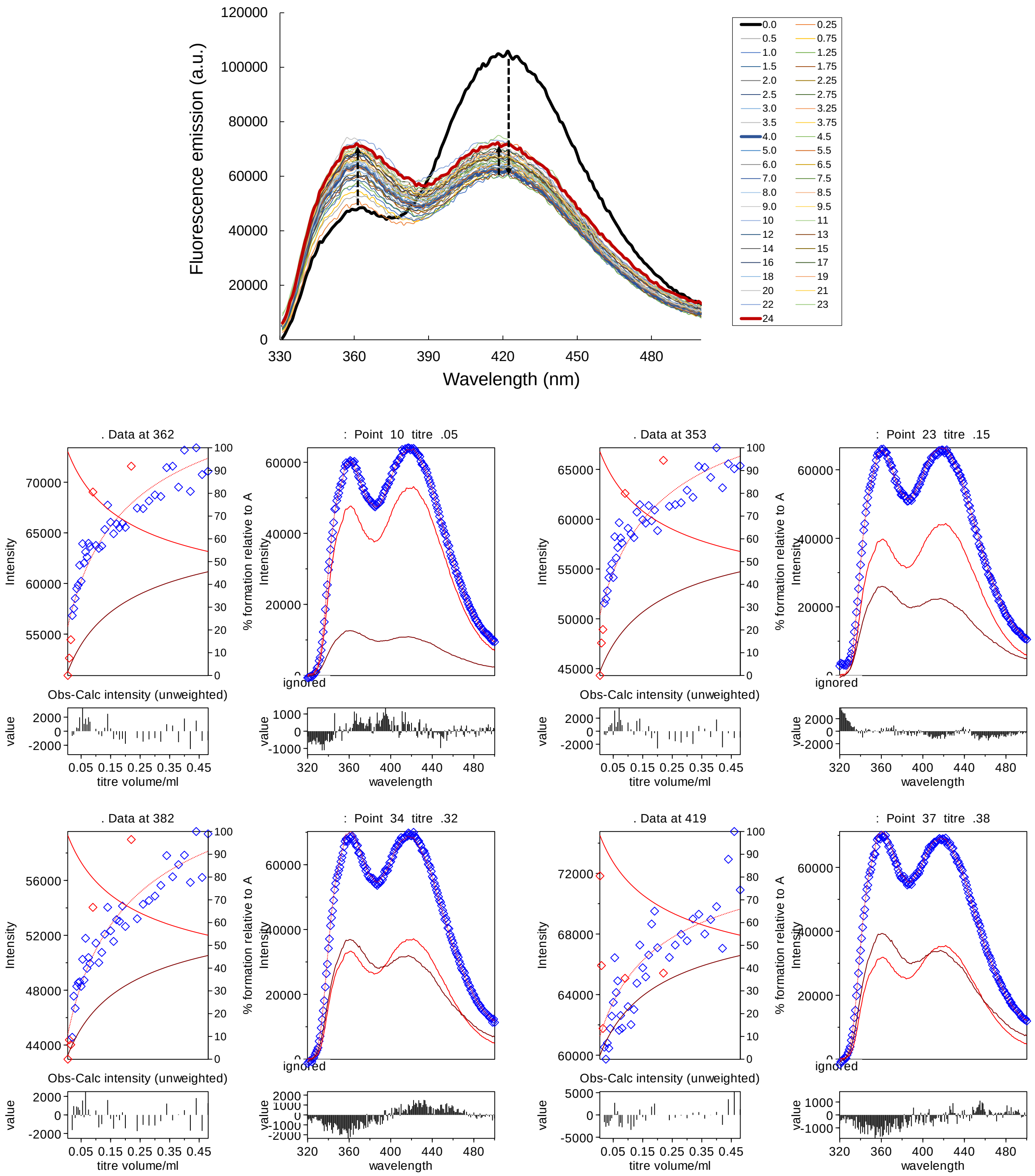

Figure S4. Fluorescence emission spectra of 3FF (4 µM, 1 mM Bis-Tris buffer at pH 7.5) alone (black trace) and upon addition of increasing concentrations of fond (µM in legend). The spectra containing one (blue) and six (red) equivalents of fond are represented with thicker lines and the dashed arrows show the titration sequence. Merged titrations rendering 42 spectra. Selected points for the fitting of the titration are shown (HypSpec2008).

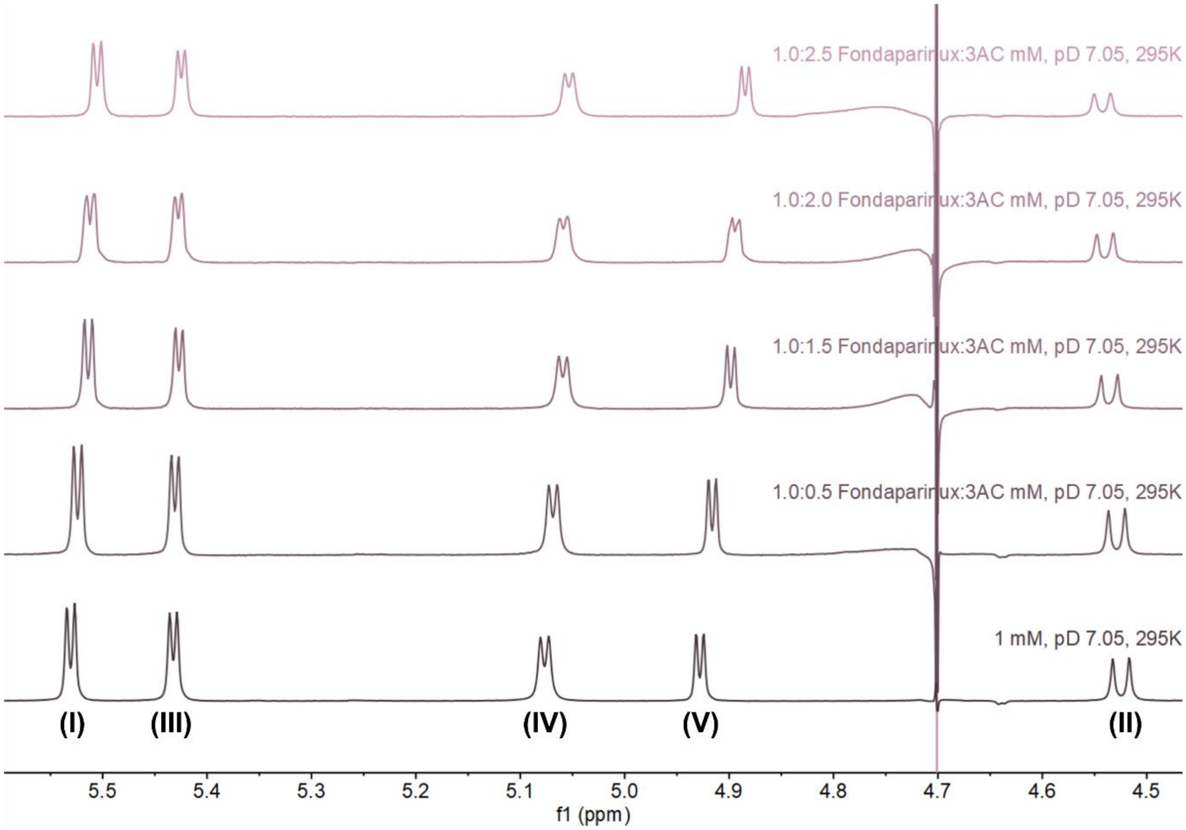

Figure S5. Anomeric region of the ^1H 1D NMR spectra of 1 mM fond (in 100% D_2O, 5 mM Tris-d11 buffer with 50 mM NaCl at pD 7.0, 295K) after addition of increasing amounts of 3AC. From bottom to top, 0 to 2.5 mM 3AC.

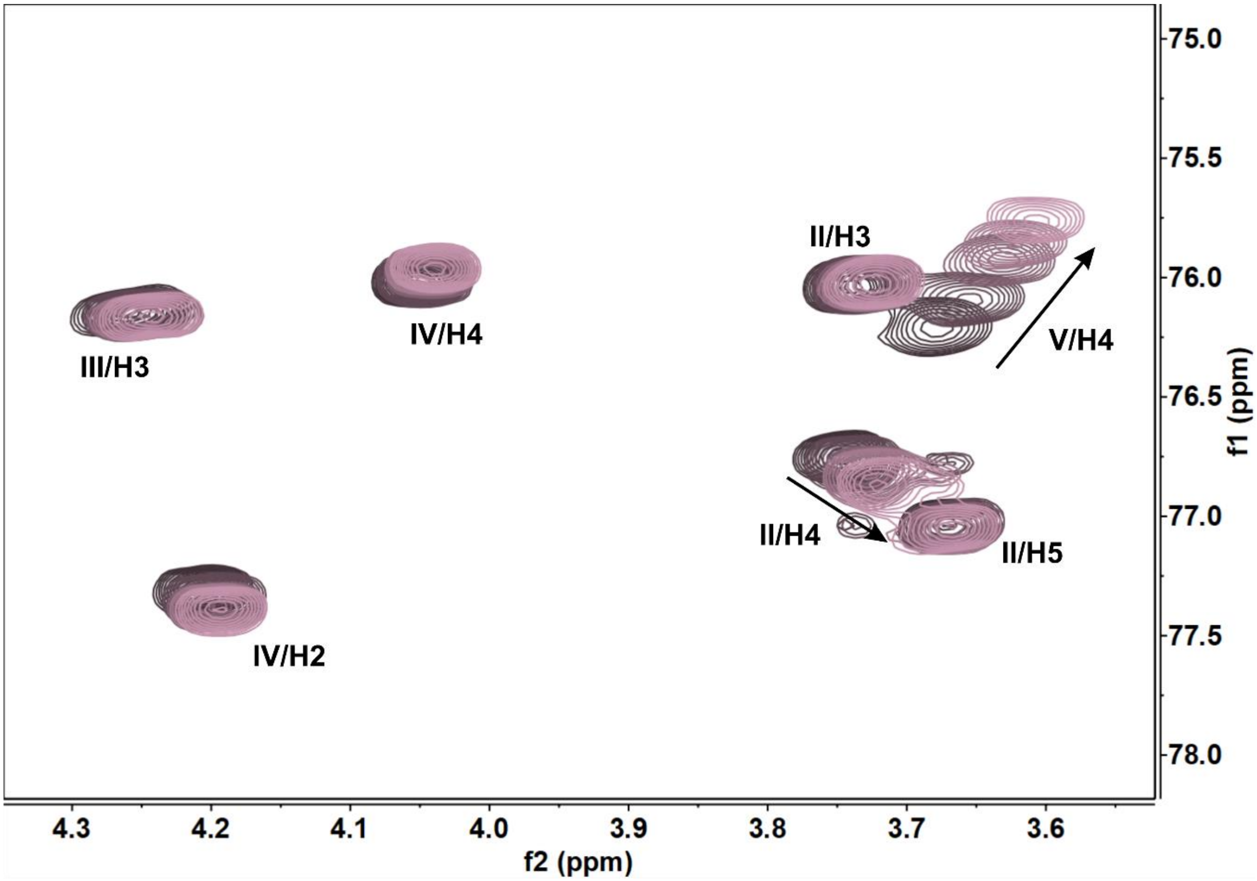

Figure S6. 2D ^1H-^{13}C HSQC NMR spectra of 1 mM fond (in 100% D_2O, 5 mM Tris-d11 buffer with 50 mM NaCl at pD 7.0, 295K) after addition of increasing amounts of 3AC (from 0 to 2.5 mM 3AC, direction indicated by the arrows).

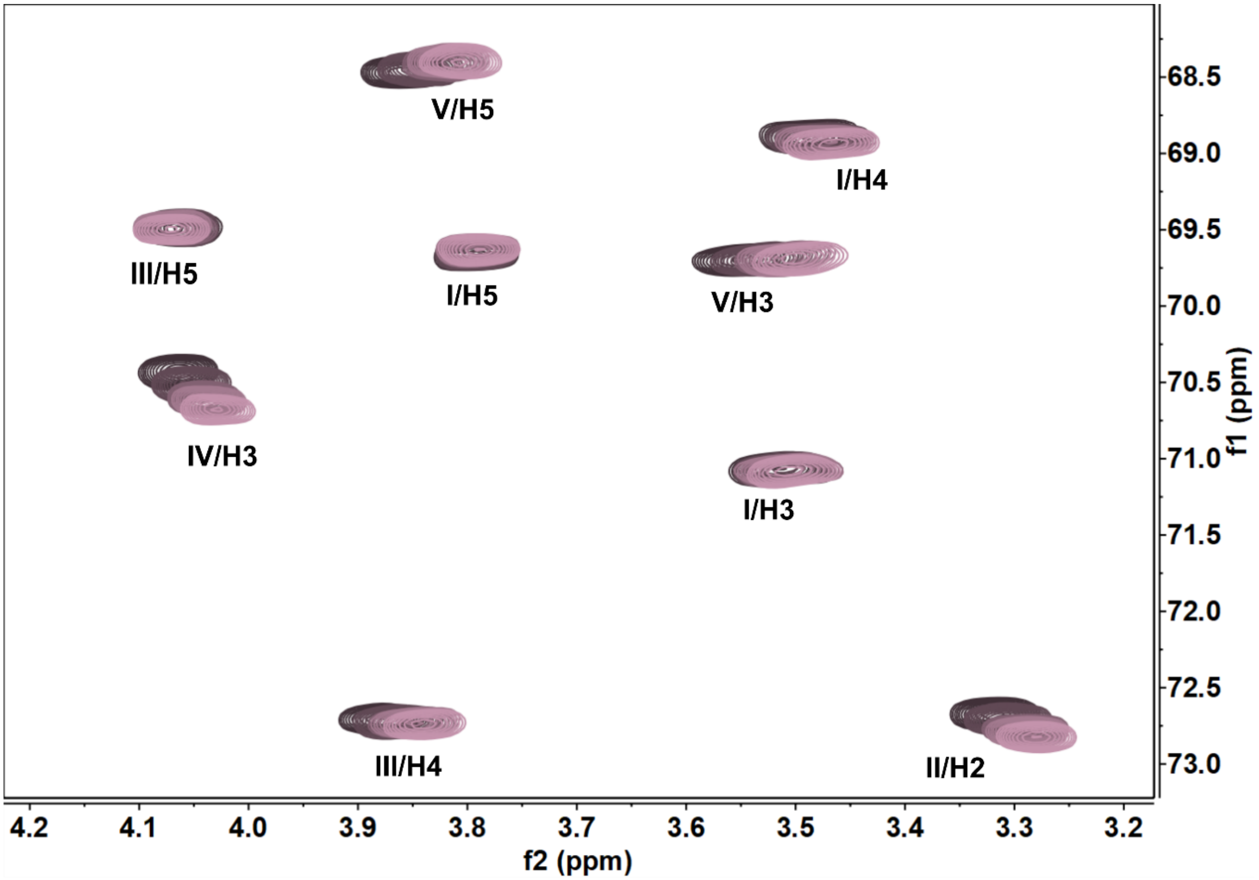

Figure S7. 2D ^{1}H-^{13}C HSQC NMR spectra of 1 mM Fondaparinux (in 100% D_2O, 5 mM Tris-d11 buffer with 50 mM NaCl at pD 7.0, 295K) after addition of increasing amounts of 3AC (from 0 to 2.5 mM 3AC).

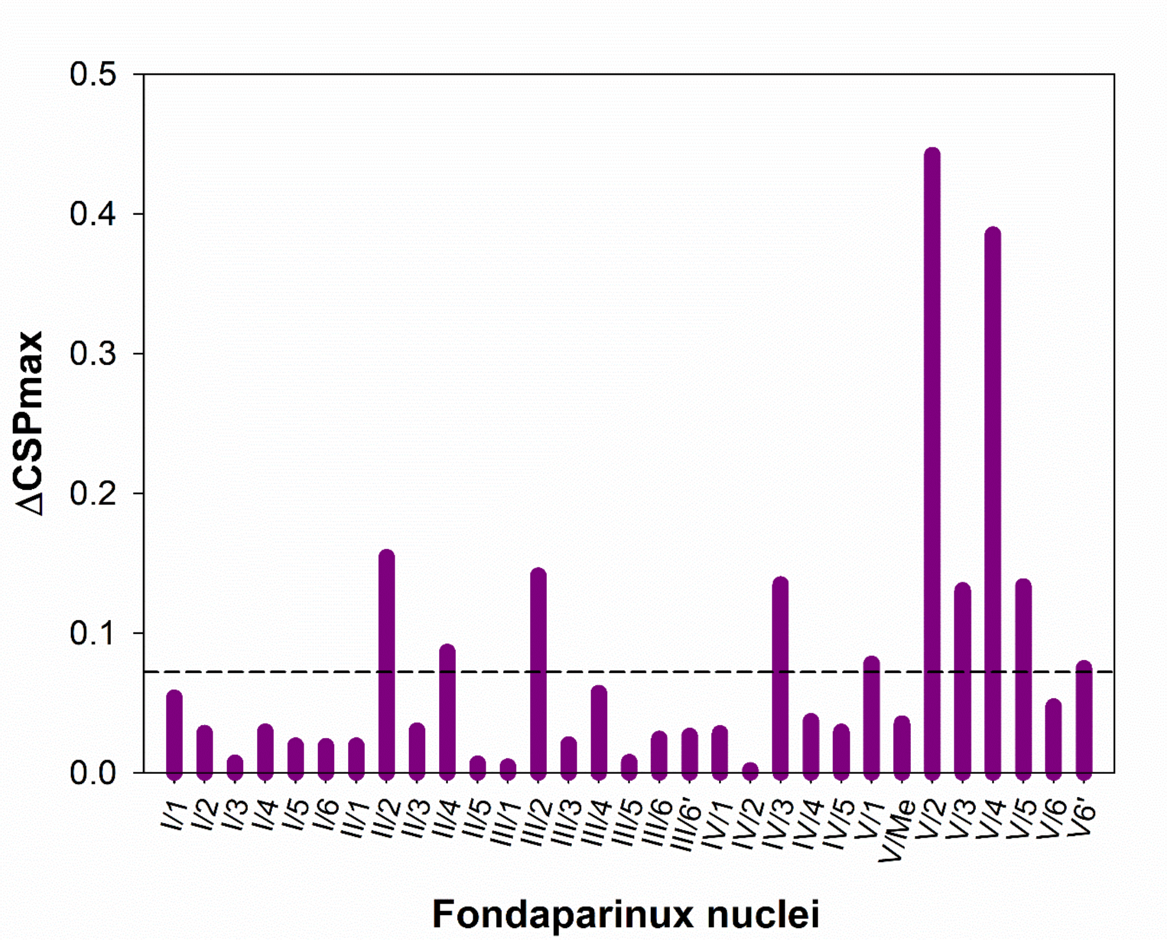

Figure S8. Chemical shift perturbation (CSP) of 1.0 mM fond in the ^{1}H and ^{13}C dimensions of HSQC spectra after addition of 2.5 mM 3AC. The CSPs were calculated using the module Binding of MNova (MestreLab).

Peng, C.; Namanja, A. T.; Munoz, E.; Wu, H.; Frederick, T. E.; Maestre-Martinez, M.; Iglesias Fernandez, I.; Sun, Q.; Cobas, C.; Sun, C.; Petros, A. M. Efficiently Driving Protein-Based Fragment Screening and Lead Discovery Using Two-Dimensional NMR. *J. Biomol. NMR* **2023**, *77* (1–2), 39–53. https://doi.org/10.1007/s10858-022-00410-3.

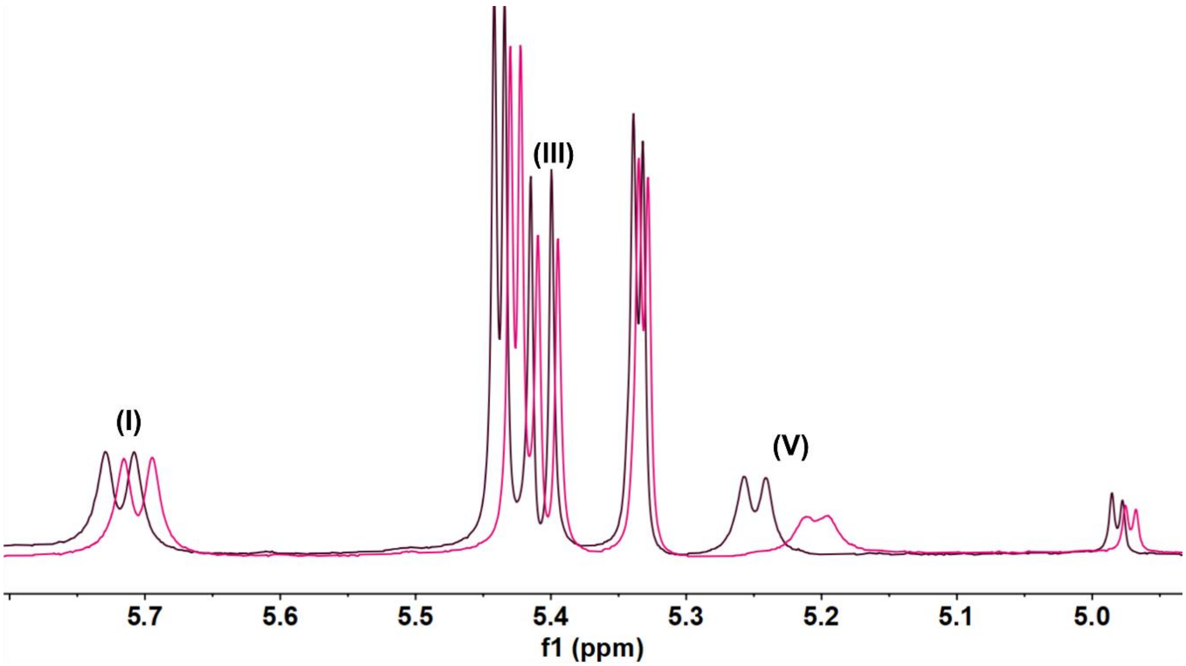

Figure S9. ^{1}H NMR spectra of 1 mM fond(in 90% H_20/10% D_2O, 5 mM Tris-d11 buffer with 50 mM NaCl at pH 8.2, 288K) acquired using the zggpw5 Bruker library pulse program, alone (dark pink) and with 1 mM 3AC (light pink). Fondaparinux sulfamate resonances of the GlcNS residues are labeled with (I), (III) and (V).

Langeslay, D. J.; Beni, S.; Larive, C. K. Detection of the 1H and 15N NMR Resonances of Sulfamate Groups in Aqueous Solution: A New Tool for Heparin and Heparan Sulfate Characterization. *Anal Chem* **2011**, *83* (20), 8006–8010. https://doi.org/10.1021/ac202144m.

Langeslay, D. J.; Young, R. P.; Beni, S.; Beecher, C. N.; Mueller, L. J.; Larive, C. K. Sulfamate Proton Solvent Exchange in Heparin Oligosaccharides: Evidence for a Persistent Hydrogen Bond in the Antithrombin-Binding Pentasaccharide Arixtra. *Glycobiology* **2012**, *22* (9), 1173–1182. https://doi.org/10.1093/glycob/cws085.

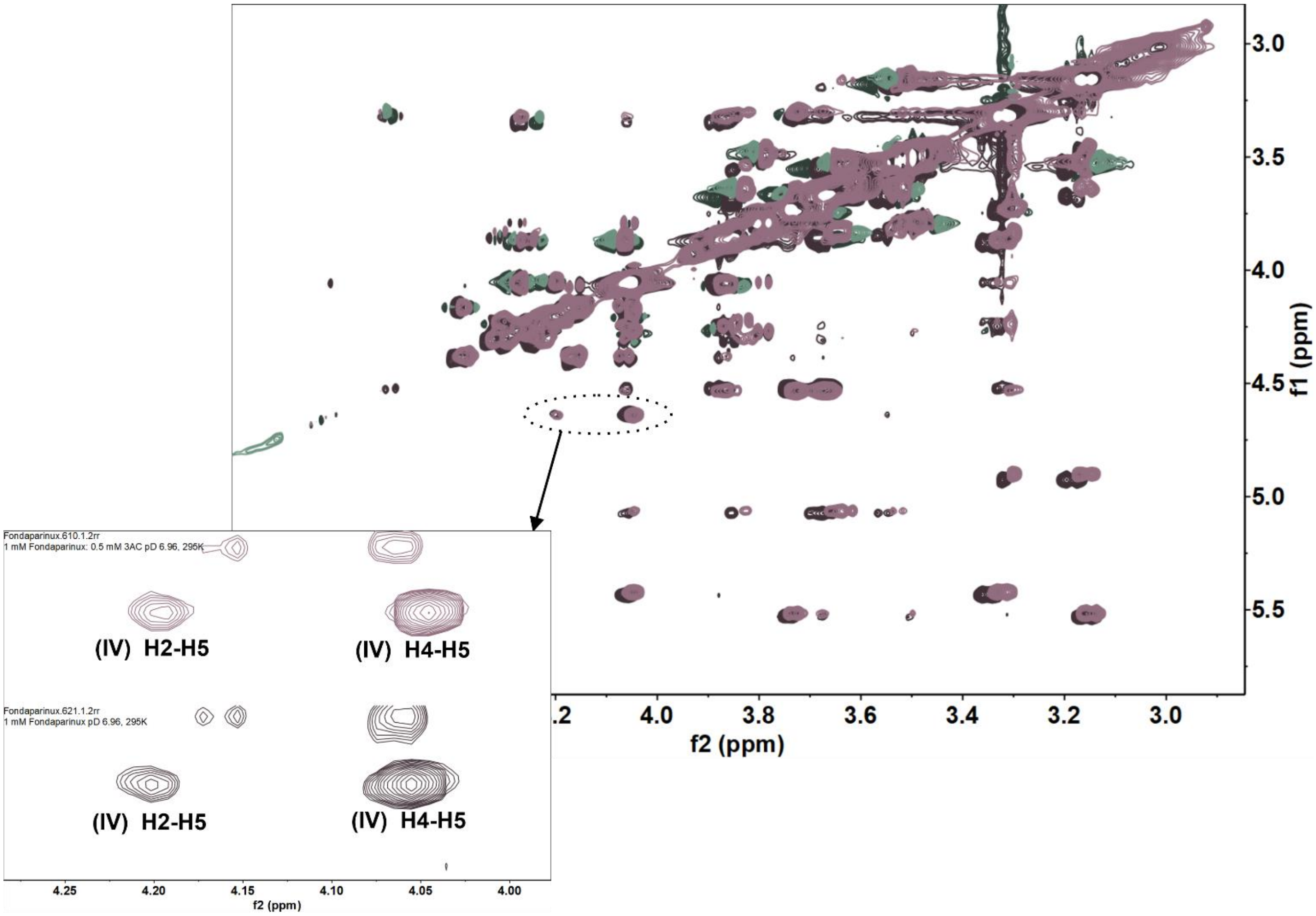

Figure S10. Overlap of the ^{1}H-^{1}H 2D NOESY spectra of 1 mM fond (dark) and 1 mM Fond: 0.5 mM 3A (light) (D_2O, 5 mM Tris-d11 buffer with 50 mM NaCl at pD 6.96, 295K). Bottom inset: 2D NOE correlations between H2-H5 and H4-H5 protons of the IdoA(2S). The analysis of experimental intra-residual NOE cross-peaks of IdoA(2S) protons provides information on the conformational change of Fond IdoA(2S) residue (see main text).

Guerrini, M.; Guglieri, S.; Beccati, D.; Torri, G.; Viskov, C.; Mourier, P. Conformational Transitions Induced in Heparin Octasaccharides by Binding with Antithrombin III. *Biochem J* **2006**, *399* (2), 191–198. https://doi.org/10.1042/BJ20060656.

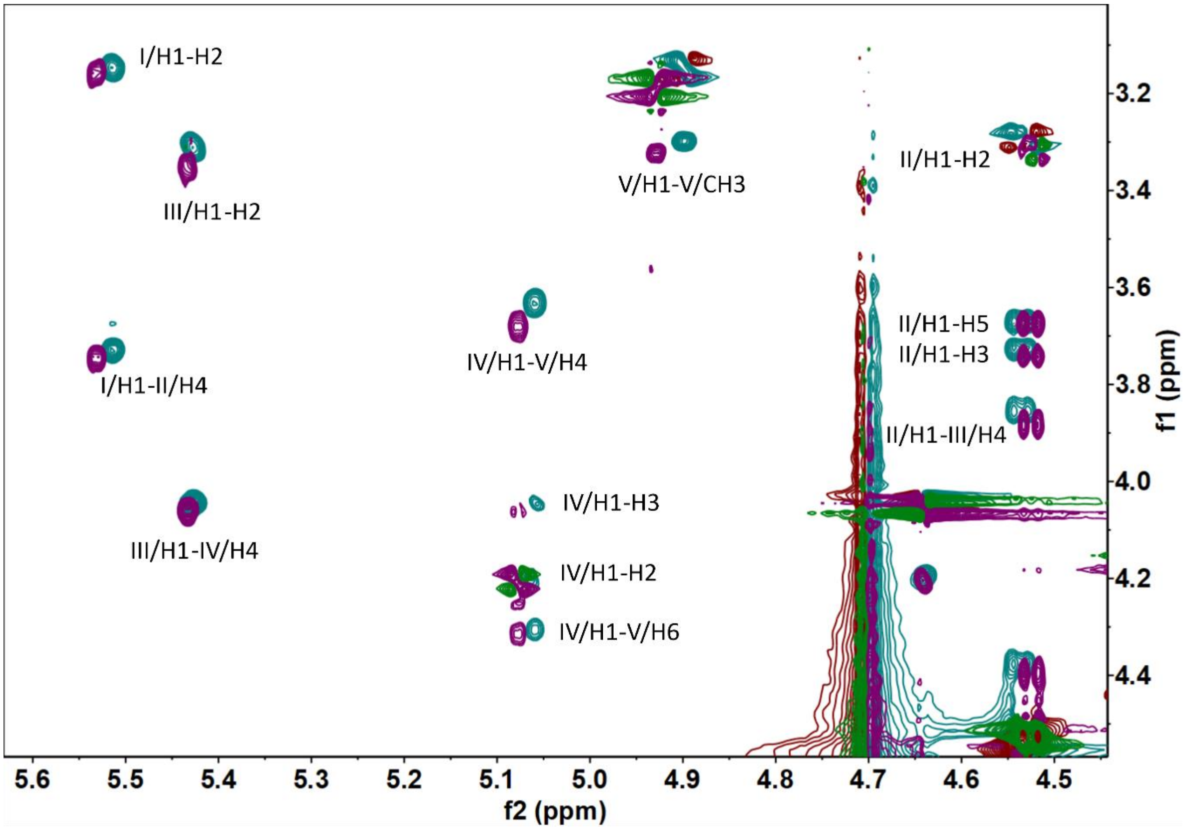

Figure S11. Anomeric region of the ^{1}H-^{1}H 2D ROESY Fond:3AC 1.0:1.5 mM (turquoise) and Fond 1.0 mM (purple) (D_2O, 5 mM Tris-d11 buffer with 50 mM NaCl at pD 7.05, 295K).

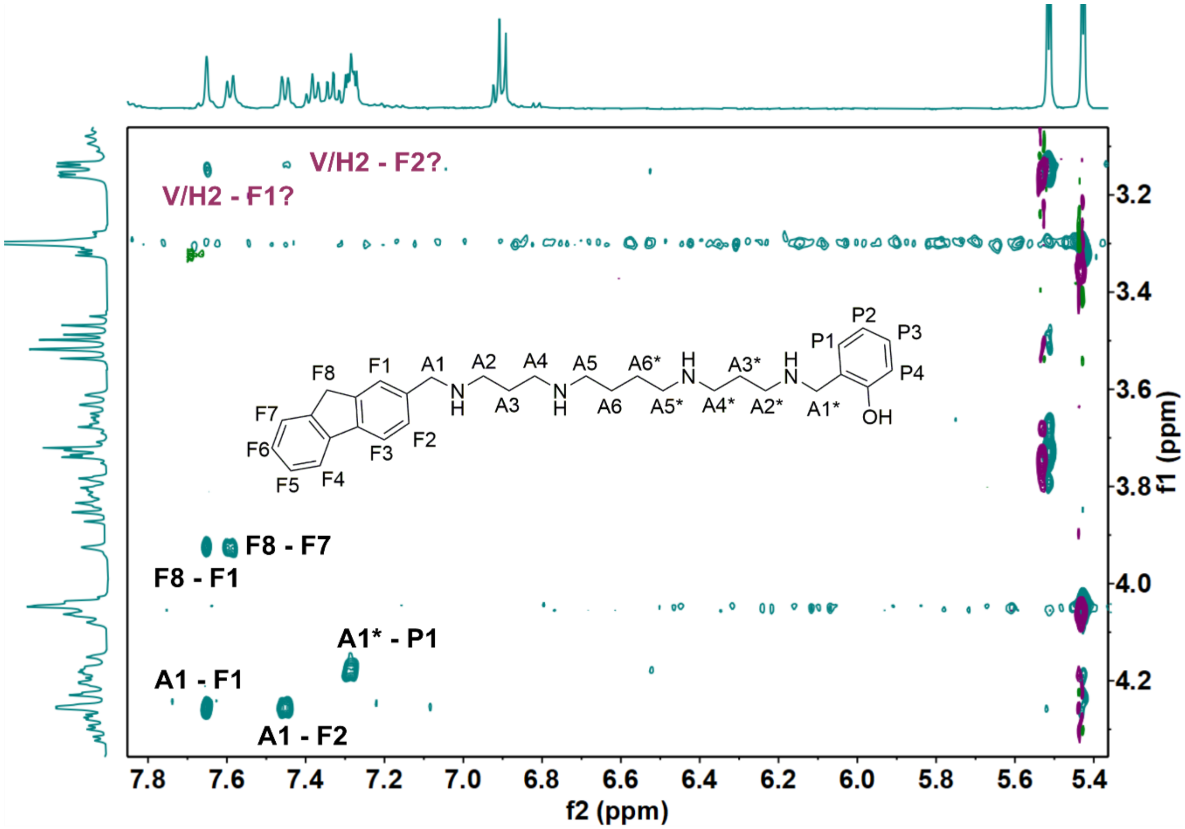

Figure S12. Aromatic region of the ^1H-^1H 2D ROESY fond:3AC 1.0:1.5 mM (turquoise) and fond 1.0 mM (purple) (D_2O, 5 mM Tris-d11 buffer with 50 mM NaCl at pD 7.05, 295K). The resonances labeled in black are 3AC intramolecular NOEs. The resonances labeled in purple are ambiguous fond – 3AC intermolecular ROEs.

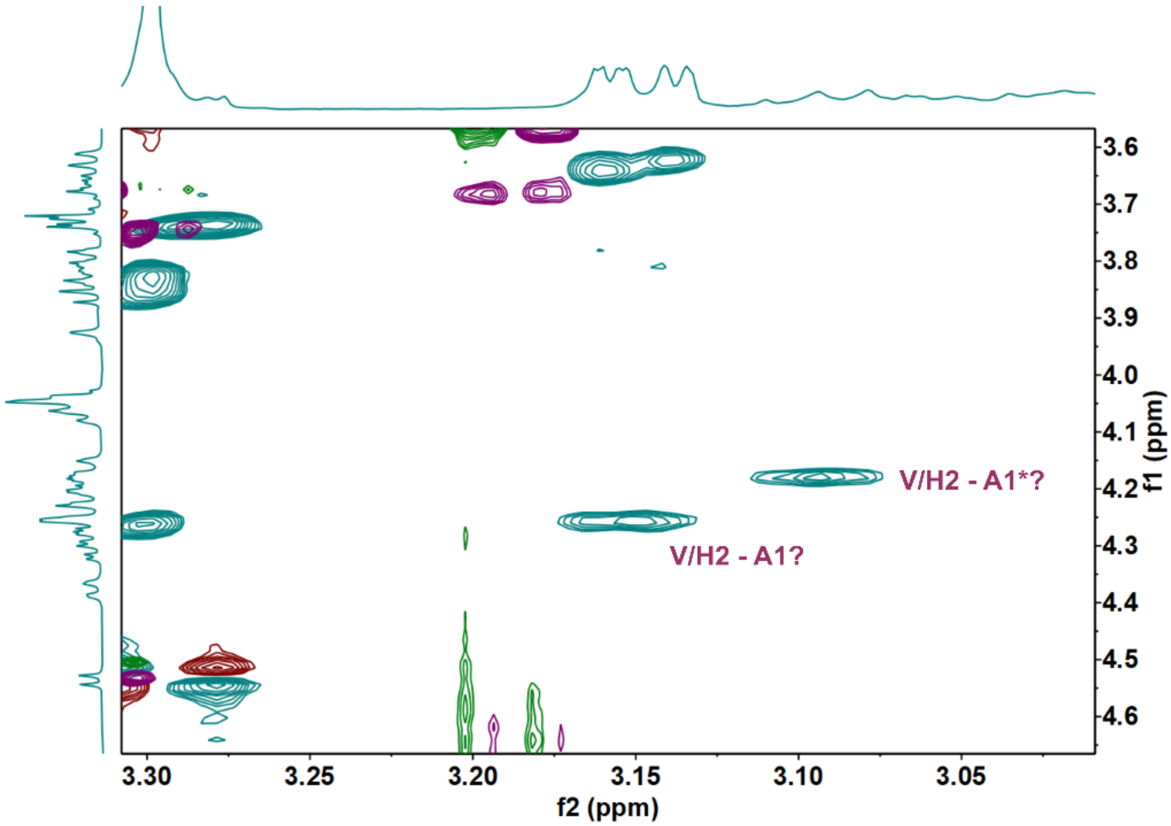

Figure S13. Aliphatic region of the ^1H-^1H 2D ROESY fond:3AC 1.0:1.5 mM (turquoise) and fond 1.0 mM (purple) (D_2O, 5 mM Tris-d11 buffer with 50 mM NaCl at pD 7.05, 295K). The resonances labeled in purple are ambiguous (due to overlapping resonances) fond – 3AC intermolecular ROEs.

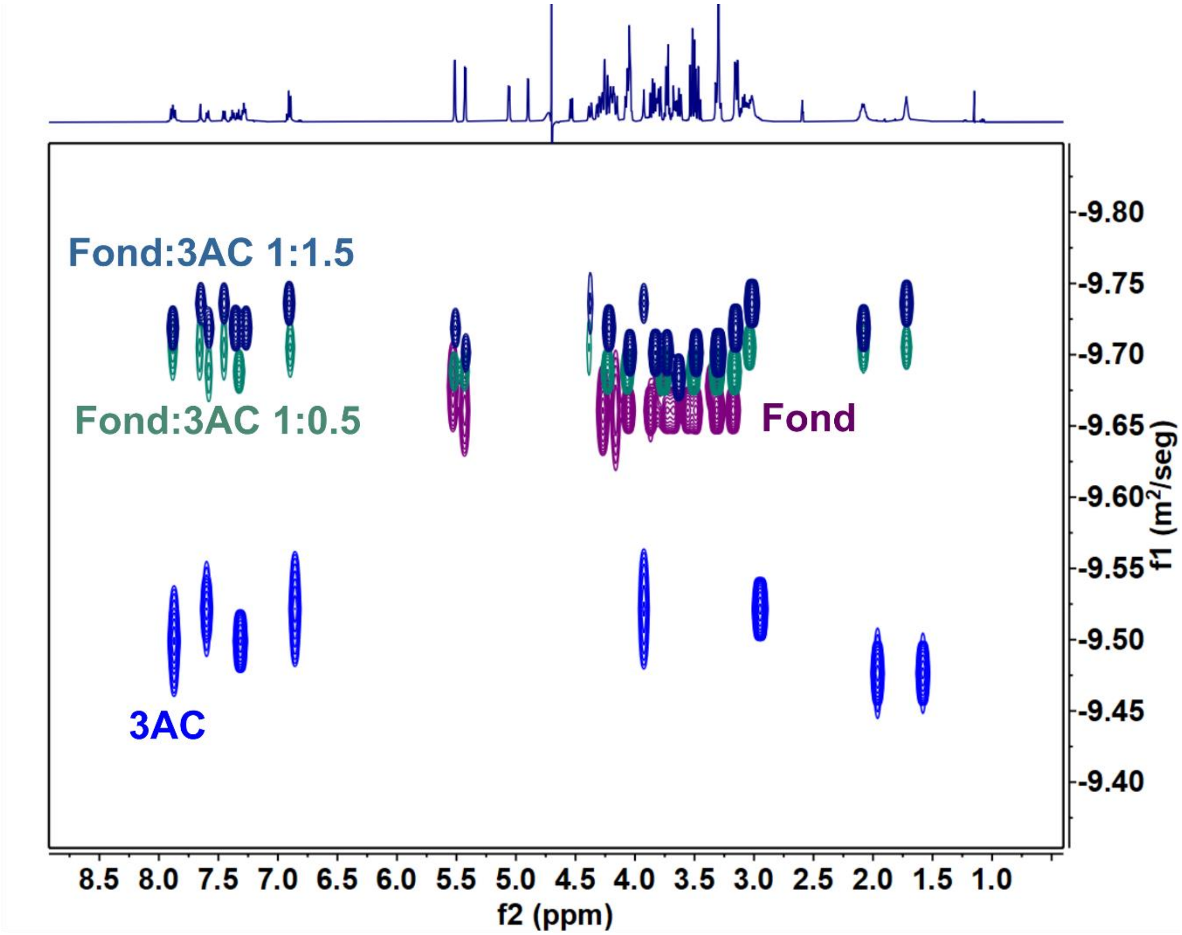

Figure S14. 2D DOSY NMR spectra of 3AC 0.5 mM (light blue), fond 1.0 mM (purple), fond:3AC 1.0:0.5 mM (turquoise) and 1.0:1.5 mM (dark blue).

Table S1. Diffusion coefficients for the indicated regions calculated by fitting 2D DOSY NMR data with the Bruker software Dynamics Center. 2D DOSY NMR experiments were acquired at pD 7.05 and 295K in D_2O. Standard deviation for all the fittings are 10^{-13}-10^{-14} m^2/s, much smaller than the observed differences.

Sample	MW	Region (ppm)	Fondaparinux D ($\times 10^{-10}$ m^2/s)	Region (ppm)	3AC D ($\times 10^{-10}$ m^2/s)
Fondaparinux 1 mM	1728.08	5.51-5.44	2.06		
		3.34-3.22	2.07		
3AC 0.5 mM	486.34			7.92-7.70	3.03
				3.11-2.66	2.92
3AC:Fondaparinux 0.5:1.0 mM		5.51-5.44	1.96	7.97-7.71	1.90
		3.34-3.19	1.99	6.92-6.79	1.92
3AC:Fondaparinux 1.5:1.0 mM		5.49-5.43	1.87		
		3.52-3.45	1.90	6.94-6.80	1.79

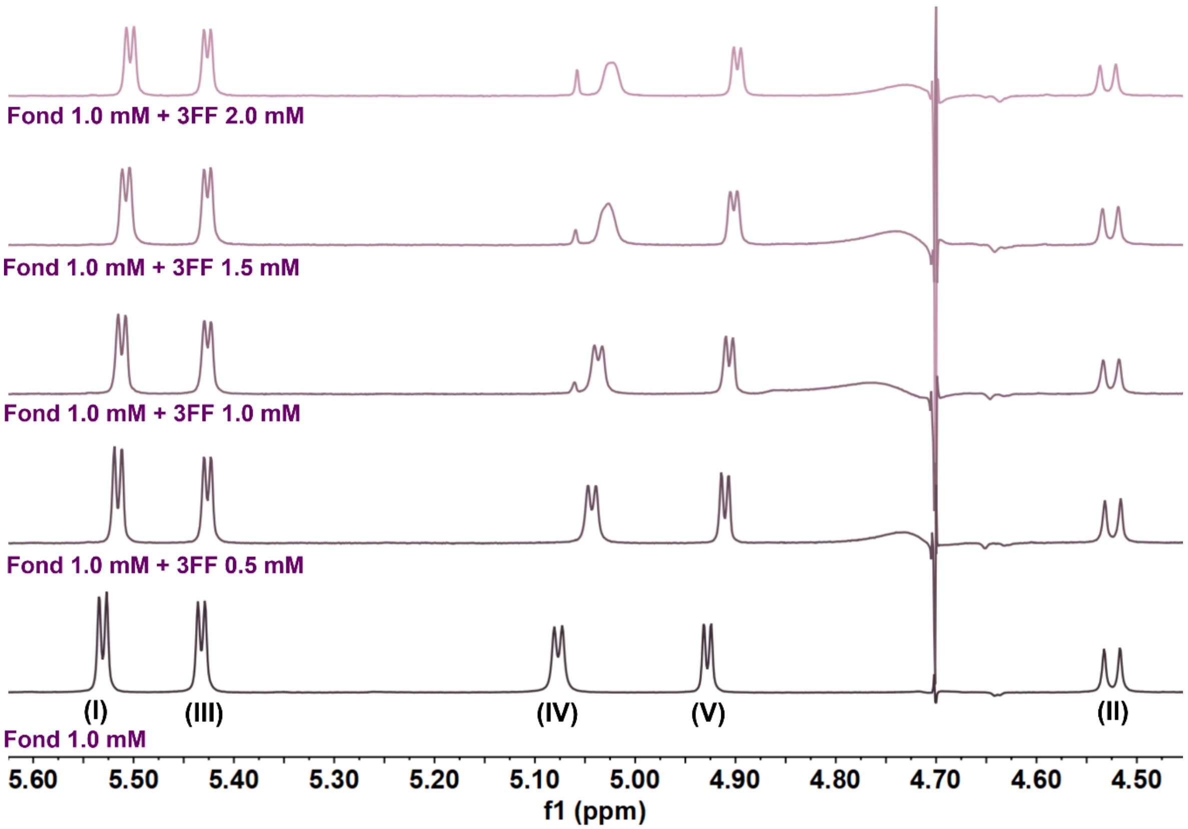

Figure S15. Anomeric region of the ^{1}H 1D NMR spectra of 1 mM fond (in 100% D_2O, 5 mM Tris-d11 buffer with 50 mM NaCl at pD 7.0, 295K) after addition of increasing amounts of 3FF. From bottom to top, 0 to 2.5 mM 3FF.

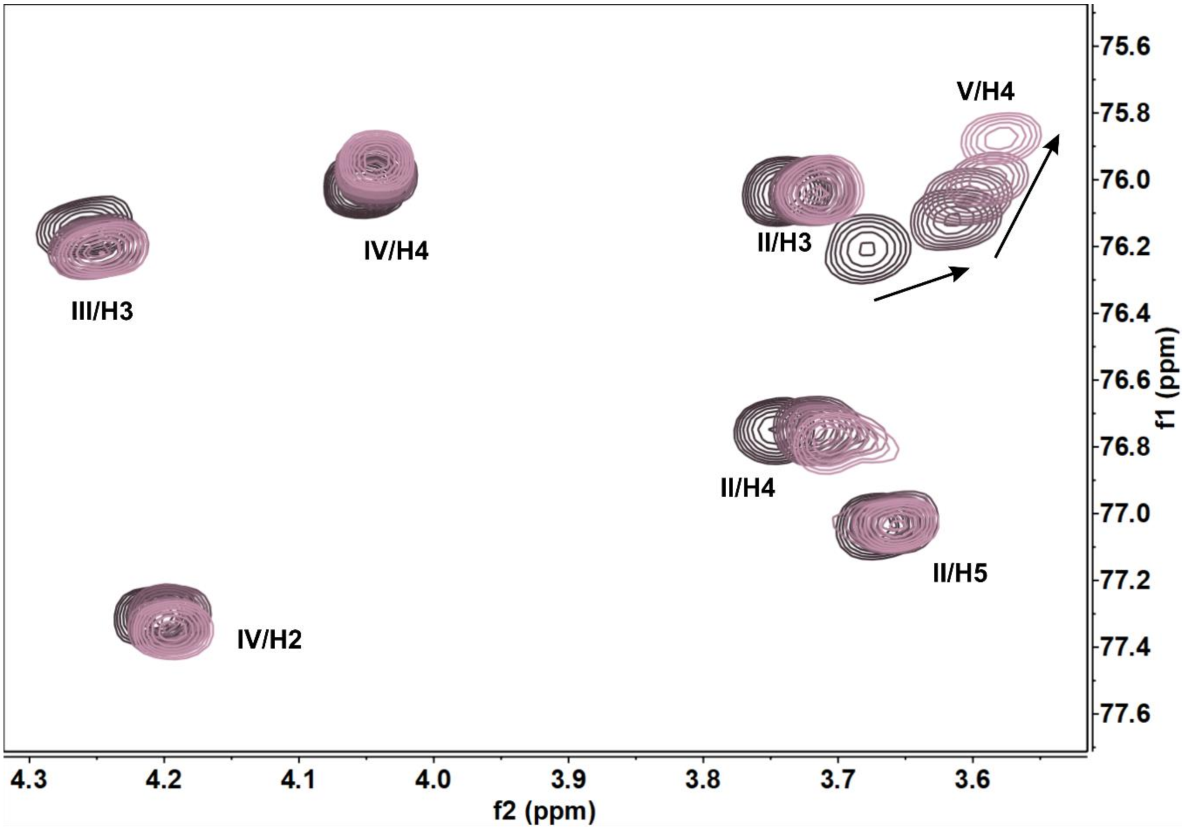

Figure S16. 2D ^{1}H-^{13}C HSQC NMR spectra of 1 mM fond (in 100% D_2O, 5 mM Tris-d11 buffer with 50 mM NaCl at pD 7.0, 295K) after addition of increasing amounts of 3FF (from 0 to 2.5 mM 3FF, direction indicated with the arrows). Unlike 3AC, we di not observe a move of V/H4 resonance in a straight line (indicating binding at a single site), but linear up to 0.5 equivalents of 3FF (first point of the titration). After this point, a change of direction is observed. Usually, this behavior is observed due to a complex equilibrium binding mode.

Williamson, M. P. Using Chemical Shift Perturbation to Characterise Ligand Binding. *Prog Nucl Magn Reson Spectrosc* **2013**, *73*, 1–16. https://doi.org/10.1016/j.pnmrs.2013.02.001.

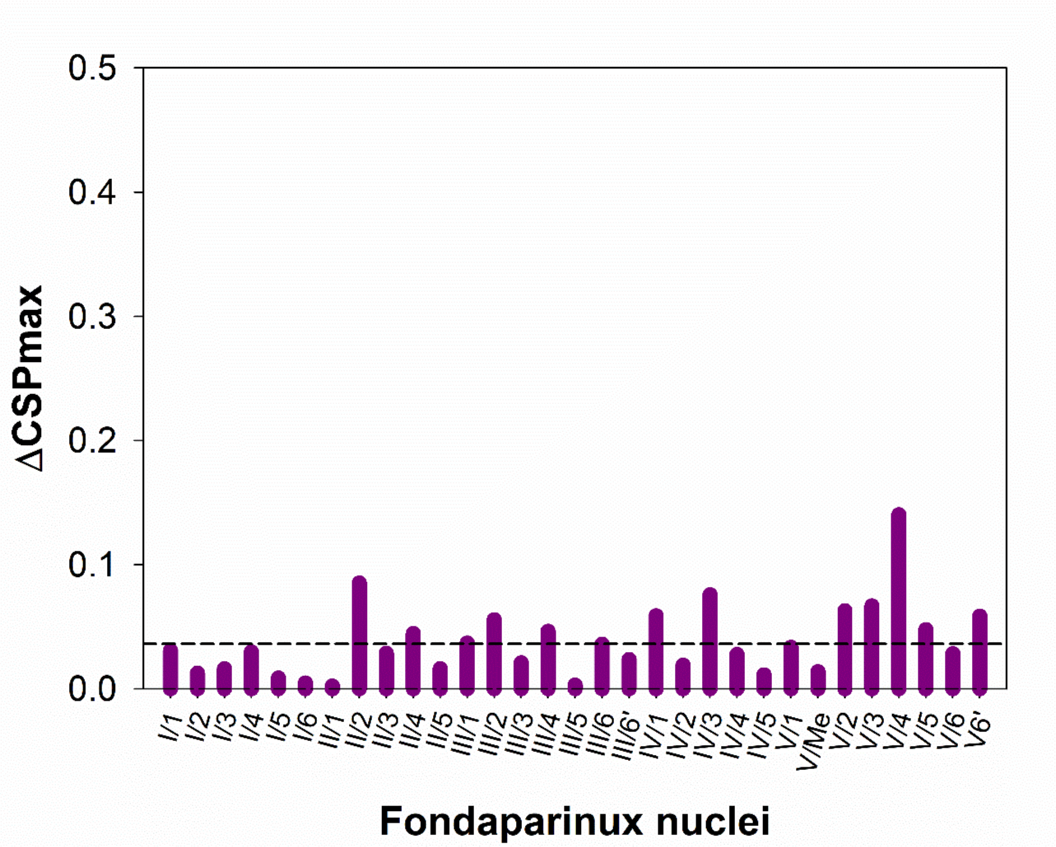

Figure S17. Chemical shift perturbation (CSP) of 1 mM fond in the ^{1}H and ^{13}C dimensions of HSQC spectra after addition of 2.5 mM 3FF. The CSPs were calculated using the module Binding of MNova (MestreLab).

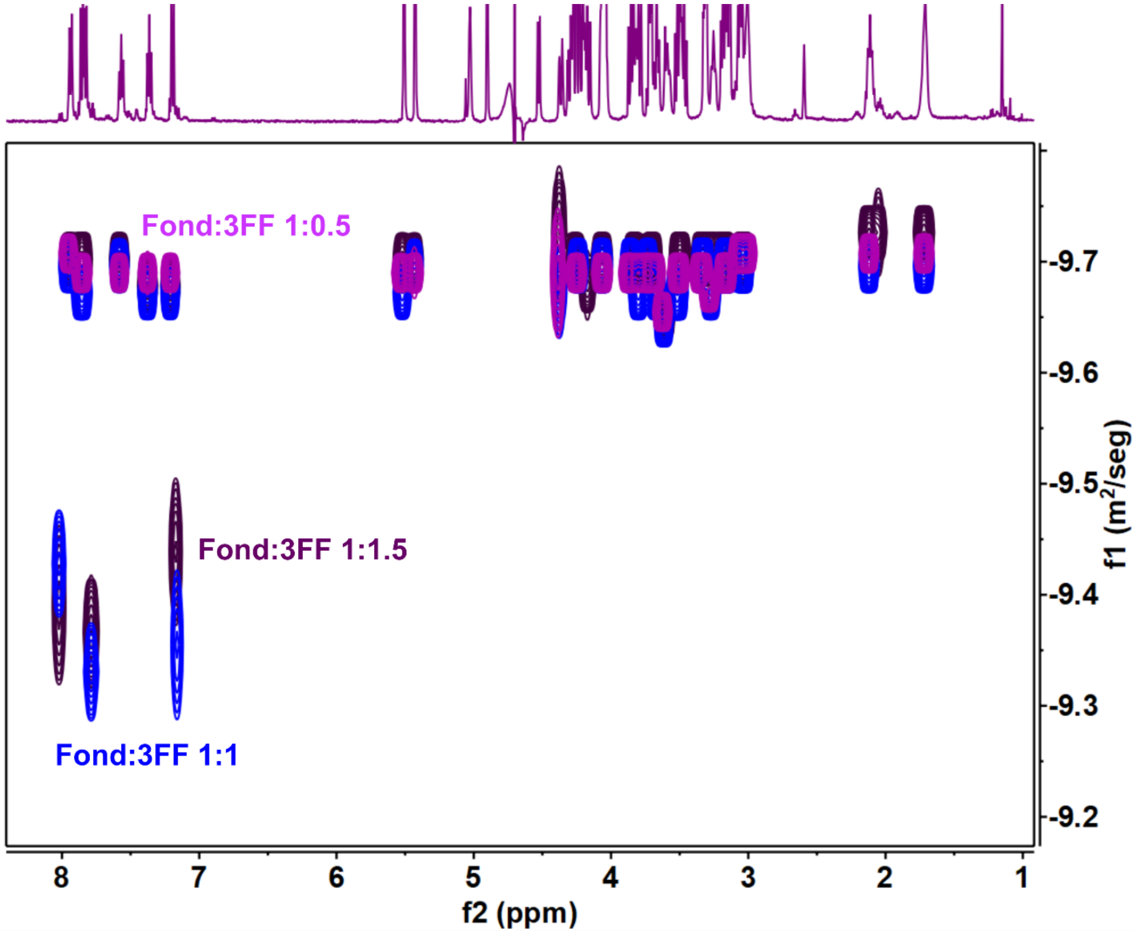

Figure S18. 2D DOSY NMR spectra of fond:3FF 1:1 mM (light blue), fond :3FF 1.0:0.5 mM (dark pink), and 1.0:1.5 mM (purple).

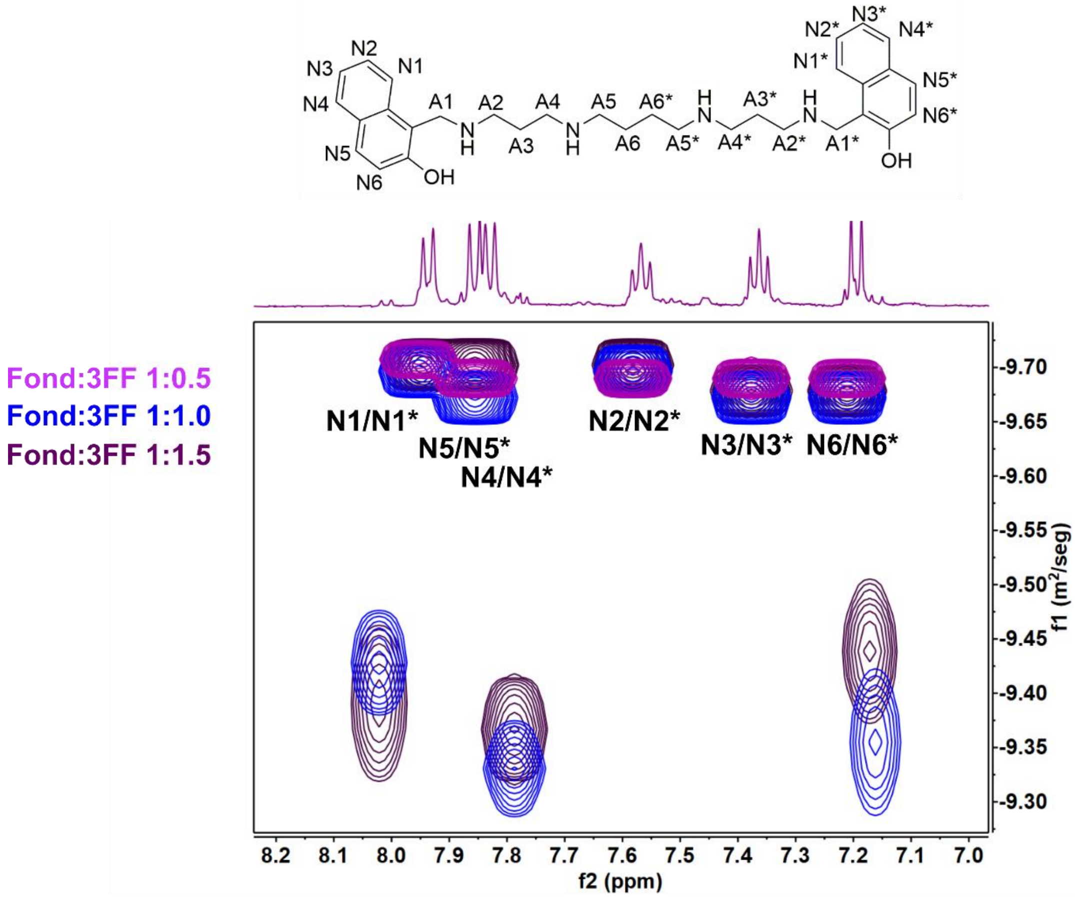

Figure S19. Enlargement of the aromatic region of the 2D DOSY NMR spectra of fond:3FF 1:1 mM (light blue), fond :3FF 1.0:0.5 mM (dark pink), and 1.0:1.5 mM (purple) with 3FF resonances labeled. We observed that at high 3FF concentrations (equimolar or in excess with respect to fond), new resonances appeared at lower diffusion coefficients compatible with 3FF monomer in solution.

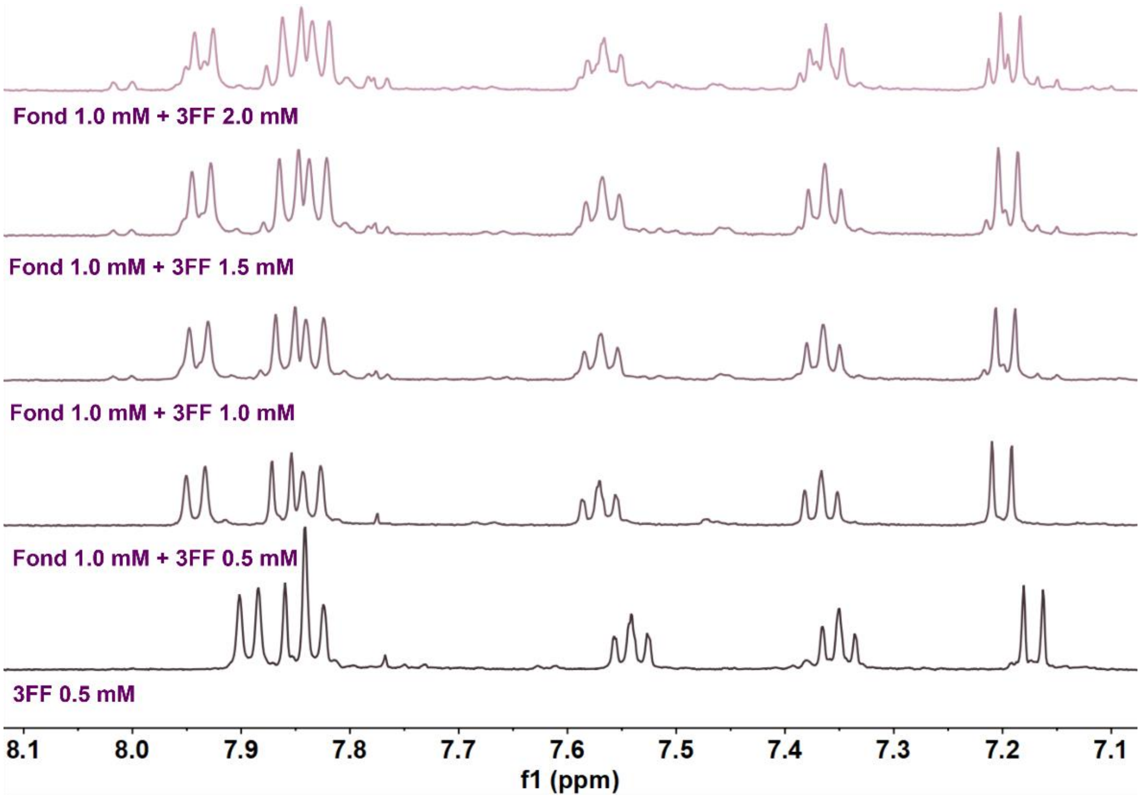

Figure S20. Aromatic region of the ^{1}H 1D NMR spectra of increasing amounts of 3FF in presence of 1 mM fond (in 100% D_2O, 5 mM Tris-d11 buffer with 50 mM NaCl at pD 7.0, 295K). As we have commented in the caption of Figure S19, we observed the appearance of additional resonances at increasing 3FF concentrations.

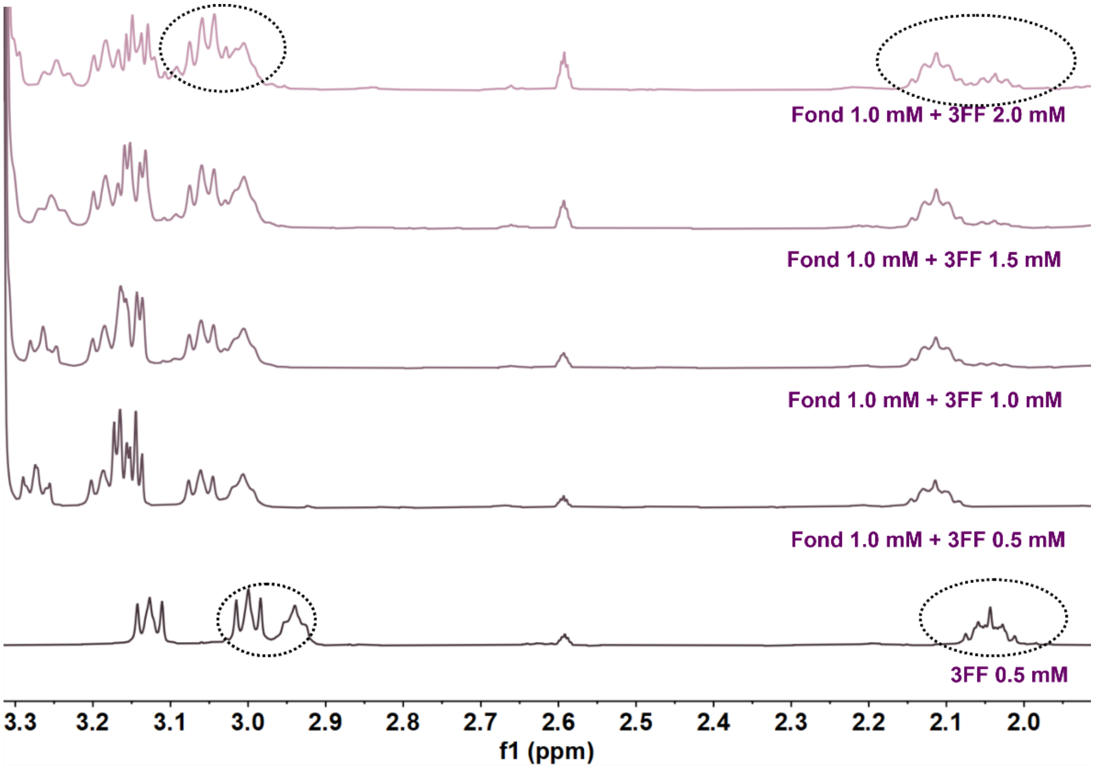

Figure S21. Aliphatic region of the ^{1}H 1D NMR spectra of increasing amounts of 3FF in presence of 1 mM fond (in 100% D_2O, 5 mM Tris-d11 buffer with 50 mM NaCl at pD 7.0, 295K). As we have commented in the caption of Figure S19, we observed the appearance of additional resonances at increasing 3FF concentrations.

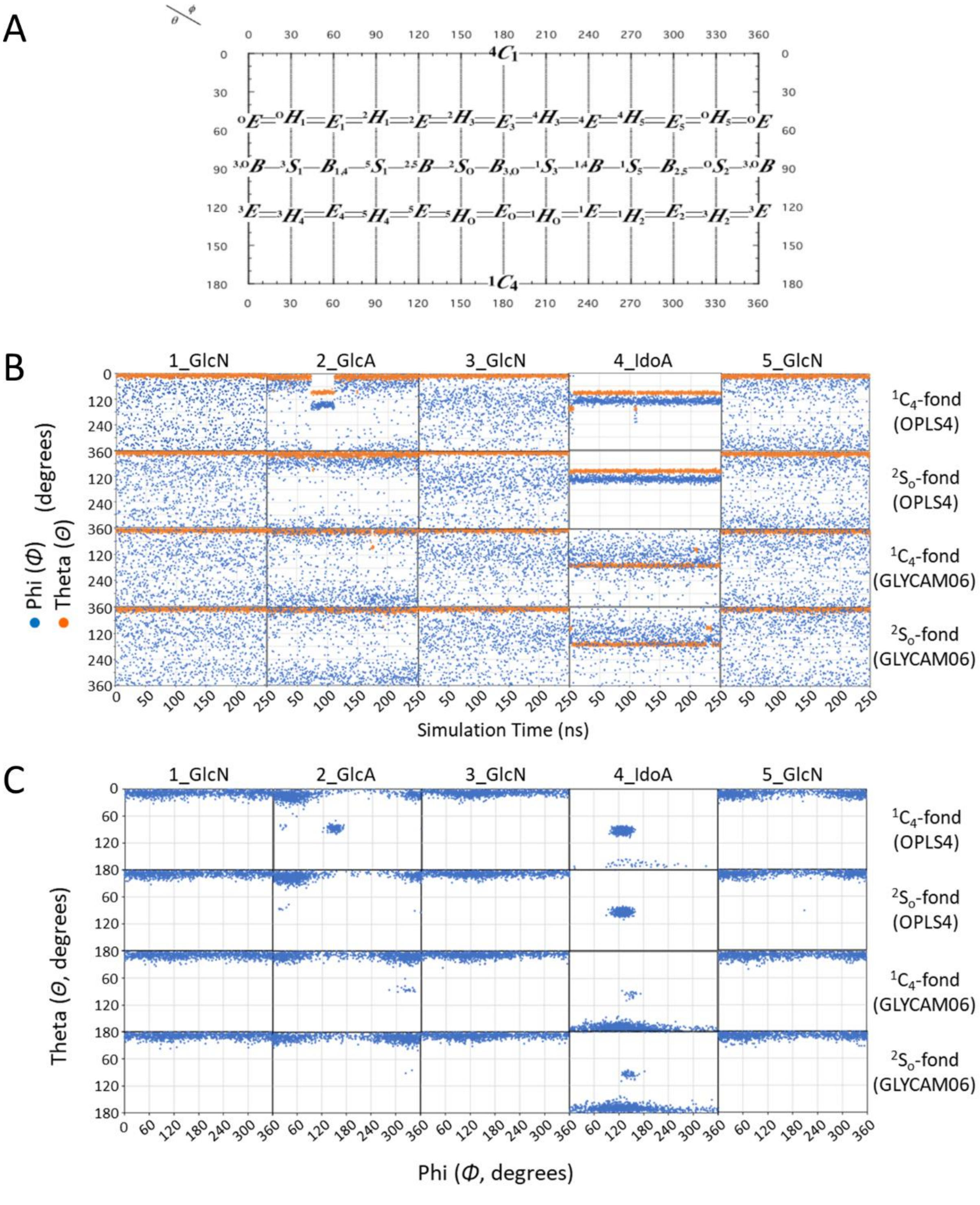

Figure S22. (A) General Cremer-Pople diagram for hexopyranoses showing the 38 canonical states of the ring (reproduced from https://enzyme13.bt.a.u-tokyo.ac.jp/CP web app developed by Prof. Shinya Fushinobu). (B) Phi and theta puckering parameters determined for the five rings of fondaparinux during the simulations of $^1C_4$-fond and ^2S_O-fond with the OPLS4 and GLYCAM06 force fields. (C) Theta vs phi puckering parameters representation for the same simulations, to be compared with the general diagram shown in (A).

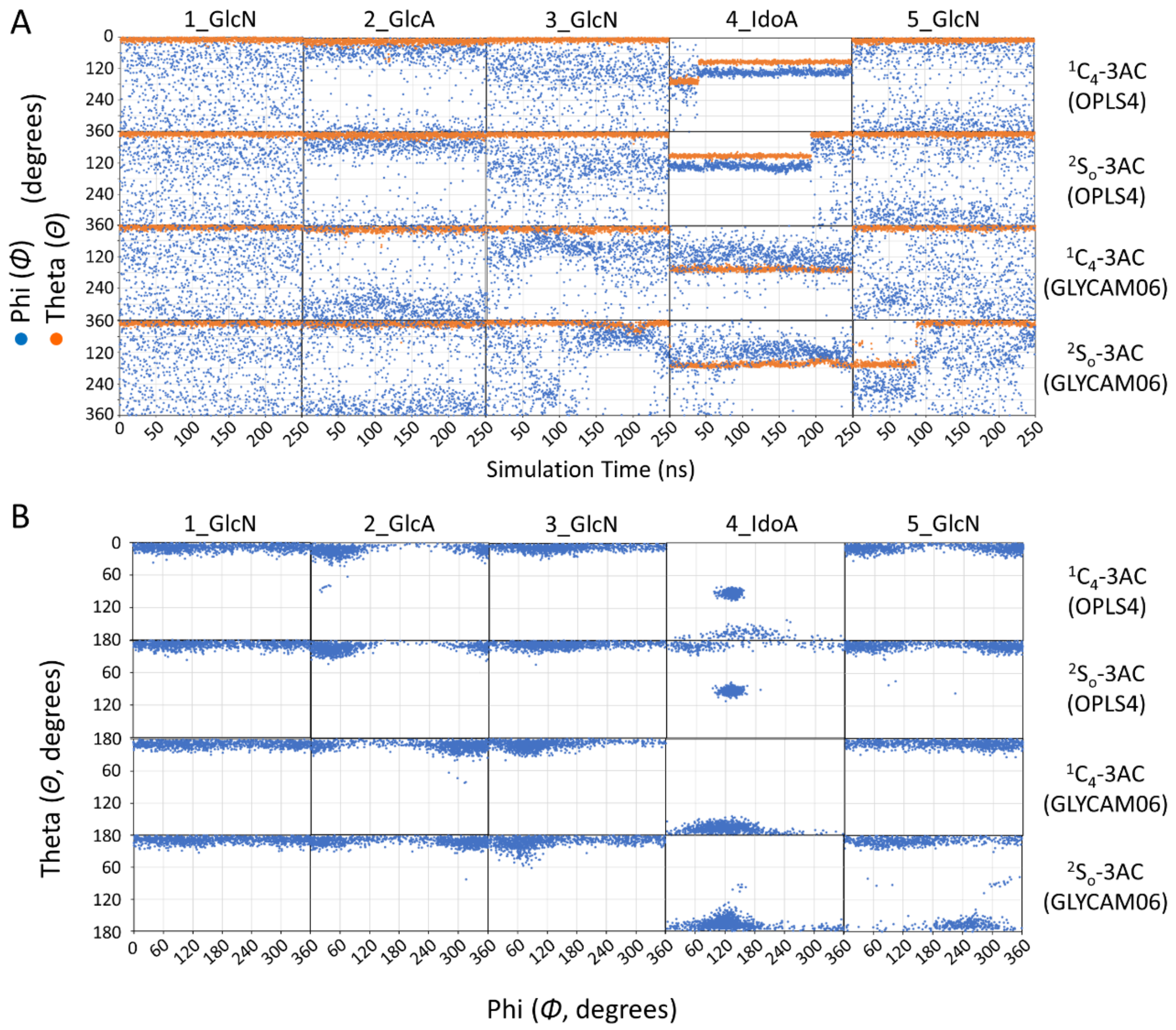

Figure S23. (A) Phi and theta puckering parameters determined for the five rings of fondaparinux during the simulations of $^{1}C_{4}$-3AC and $^{2}S_{O}$-3AC with the OPLS4 and GLYCAM06 force fields. (B) Theta vs phi puckering parameters representation for the same simulations.

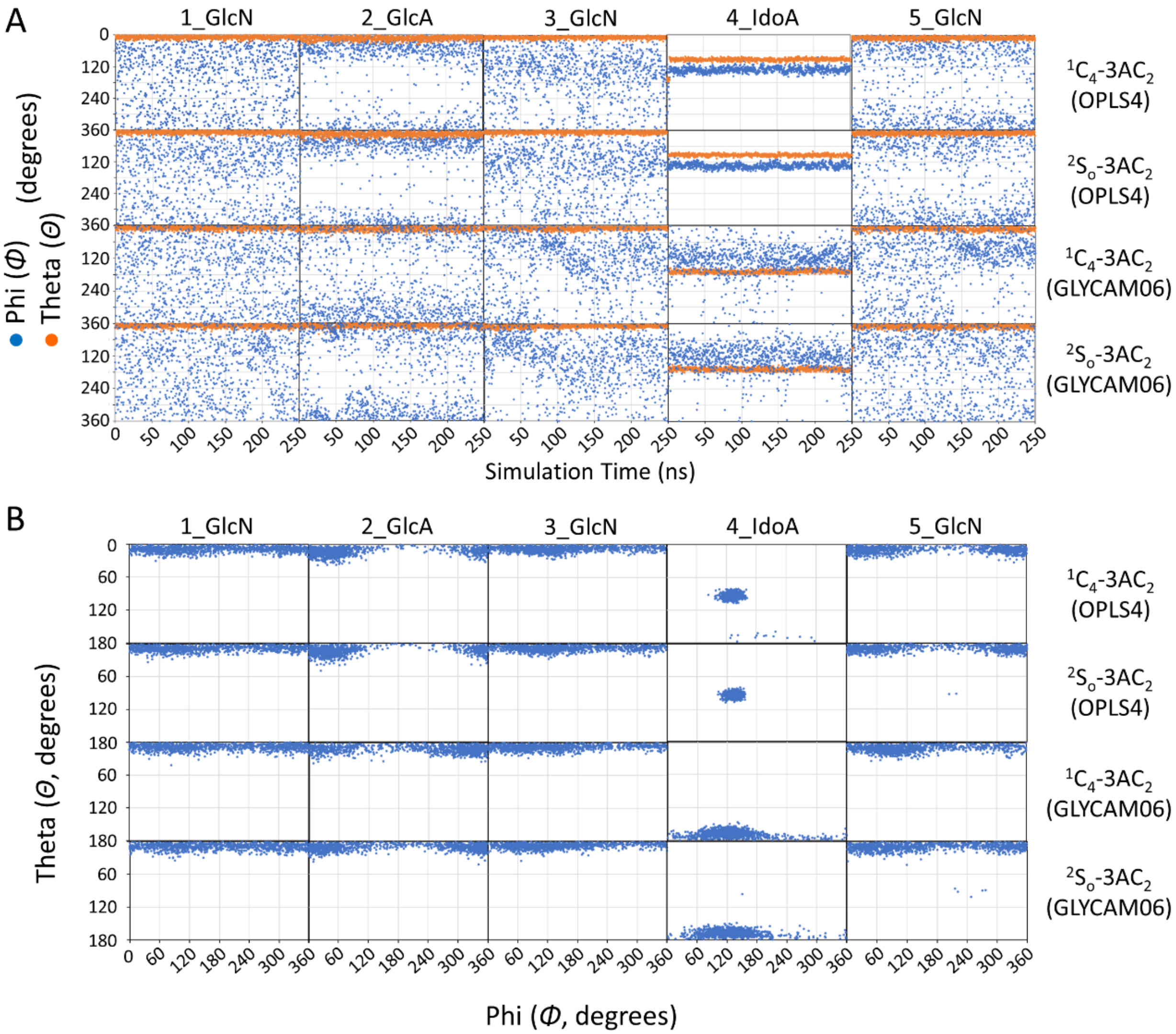

Figure S24. (A) Phi and theta puckering parameters determined for the five rings of fondaparinux during the simulations of $^{1}C_{4}$-3AC$_2$ and $^{2}S_{O}$-3AC$_2$ with the OPLS4 and GLYCAM06 force fields. (B) Theta vs phi puckering parameters representation for the same simulations.

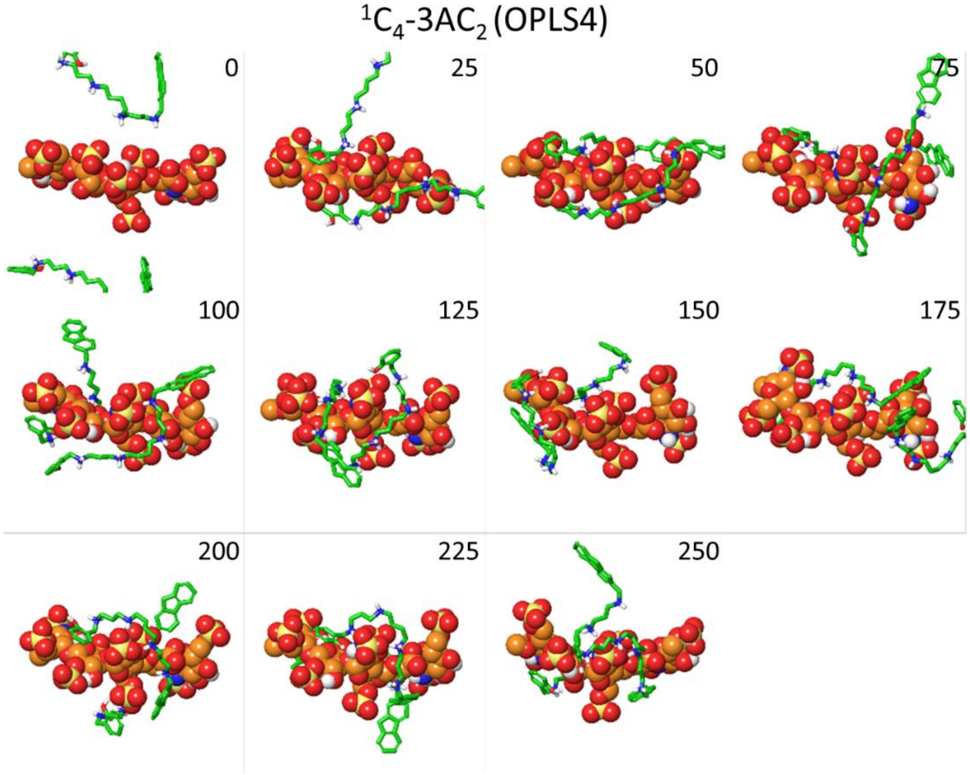

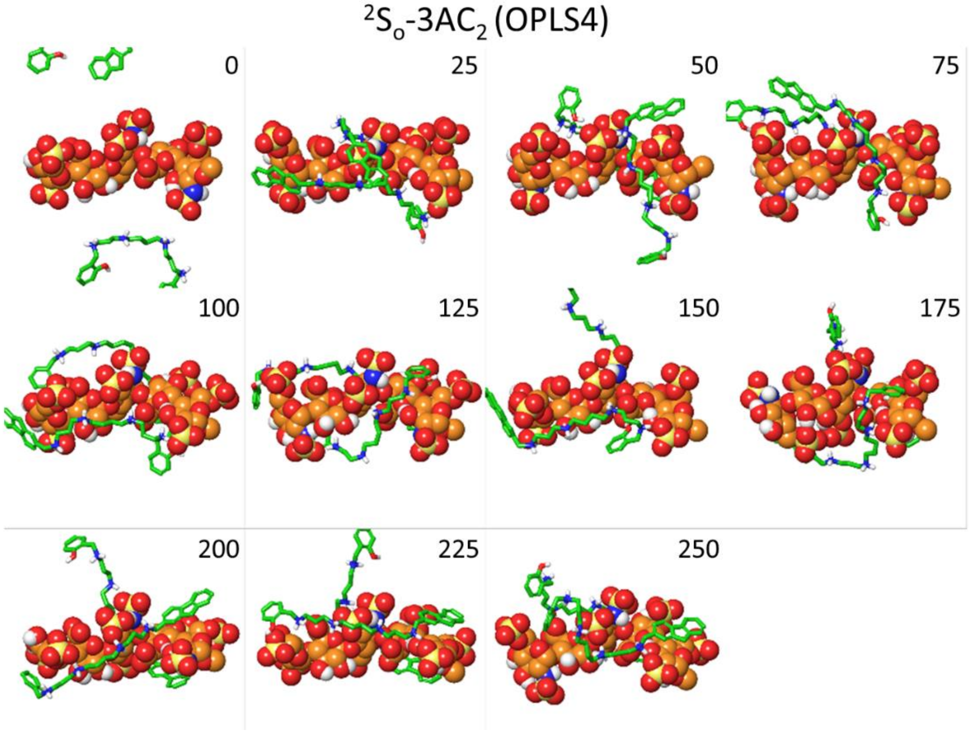

Figure S25. Snapshots of the $^{1}C_{4}$-3AC$_{2}$ and $^{2}S_{O}$-3AC$_{2}$ simulations using the OPLS4 force field. The simulation time (ns) is displayed for each snapshot. Fond is shown as spheres (orange C-atoms) and 3AC as sticks (green C-atoms). Water molecules, ions and 3AC atoms that are too far from fond are omitted for clarity.

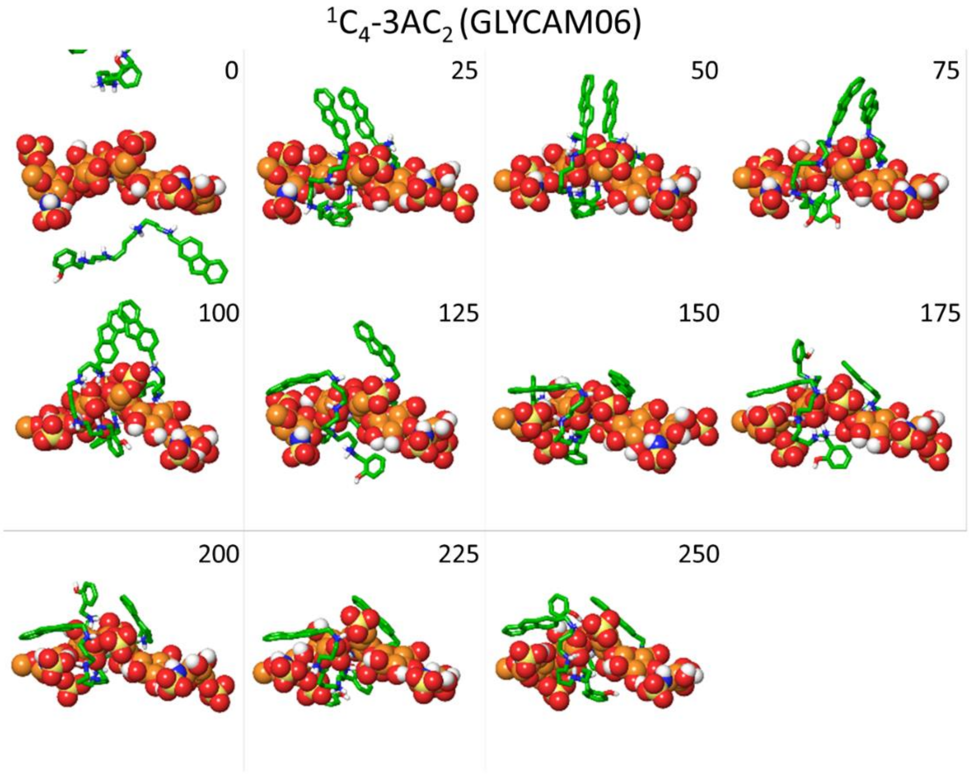

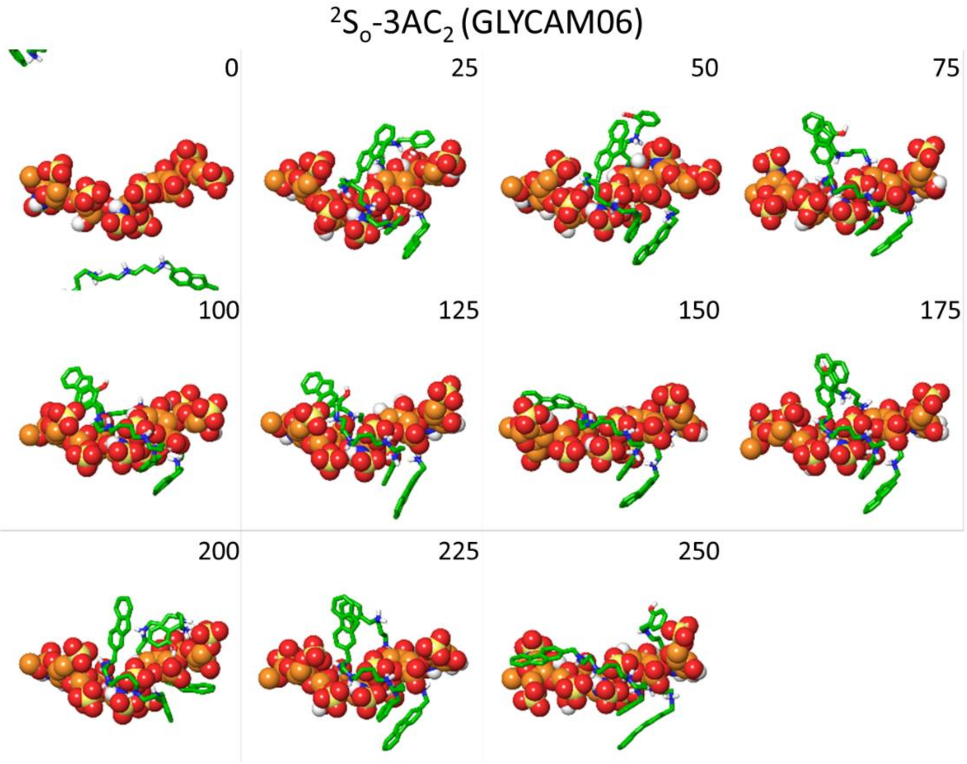

Figure S26. Snapshots of the $^1C_4$-3AC$_2$ and ^2S_O-3AC$_2$ simulations using the GLYCAM06 force field. The simulation time (ns) is displayed for each snapshot. Fond is shown as spheres (orange C-atoms) and 3AC as sticks (green C-atoms). Water molecules, ions and 3AC atoms that are too far from fond are omitted for clarity.

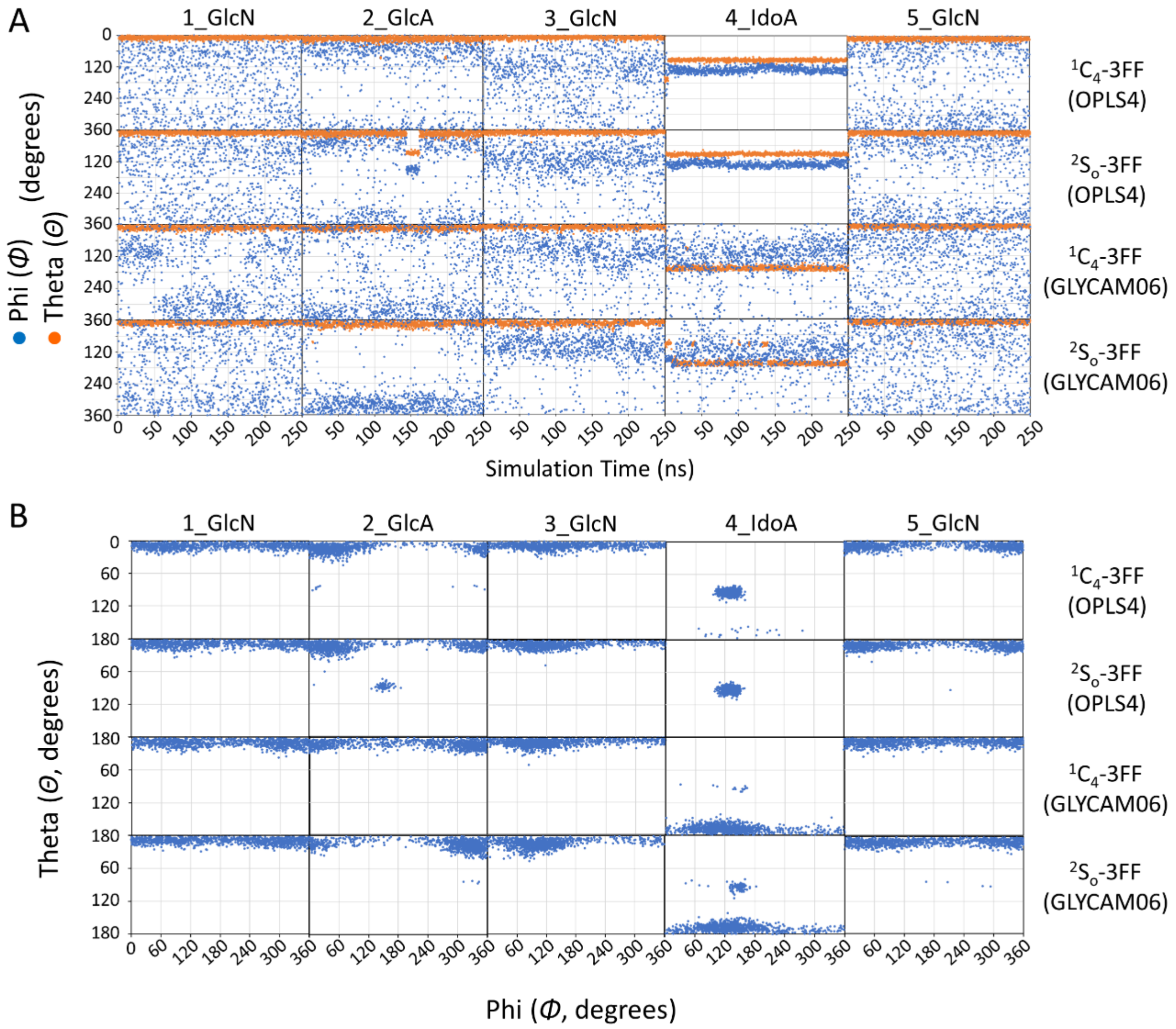

Figure S27. (A) Phi and theta puckering parameters determined for the five rings of fondaparinux during the simulations of $^1C_4$-3FF and ^2S_O-3FF with the OPLS4 and GLYCAM06 force fields. (B) Theta vs phi puckering parameters representation for the same simulations.

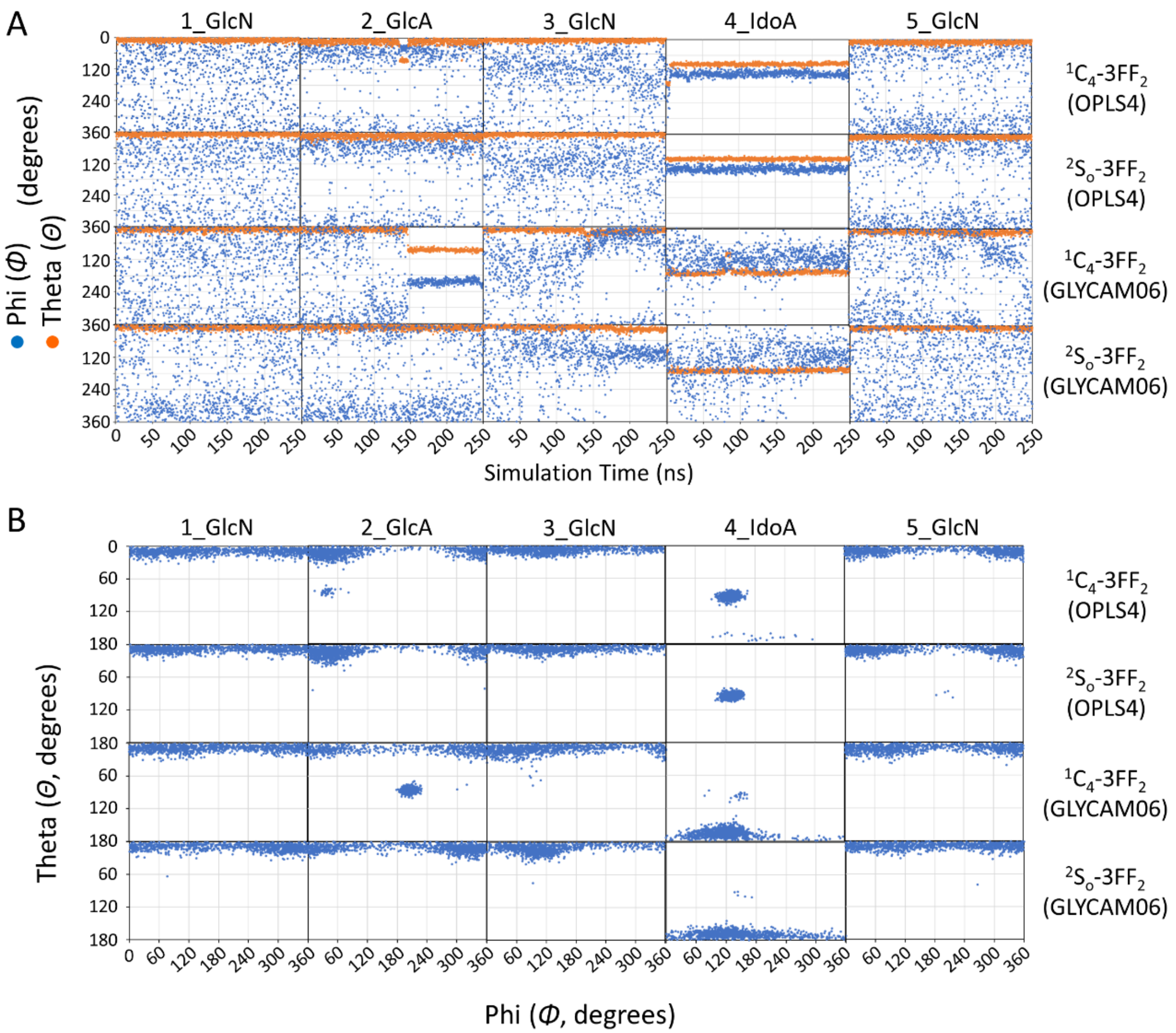

Figure S28. (A) Phi and theta puckering parameters determined for the five rings of fondaparinux during the simulations of $^{1}C_{4}$-3FF$_{2}$ and $^{2}S_{O}$-3FF$_{2}$ with the OPLS4 and GLYCAM06 force fields. (B) Theta vs phi puckering parameters representation for the same simulations.

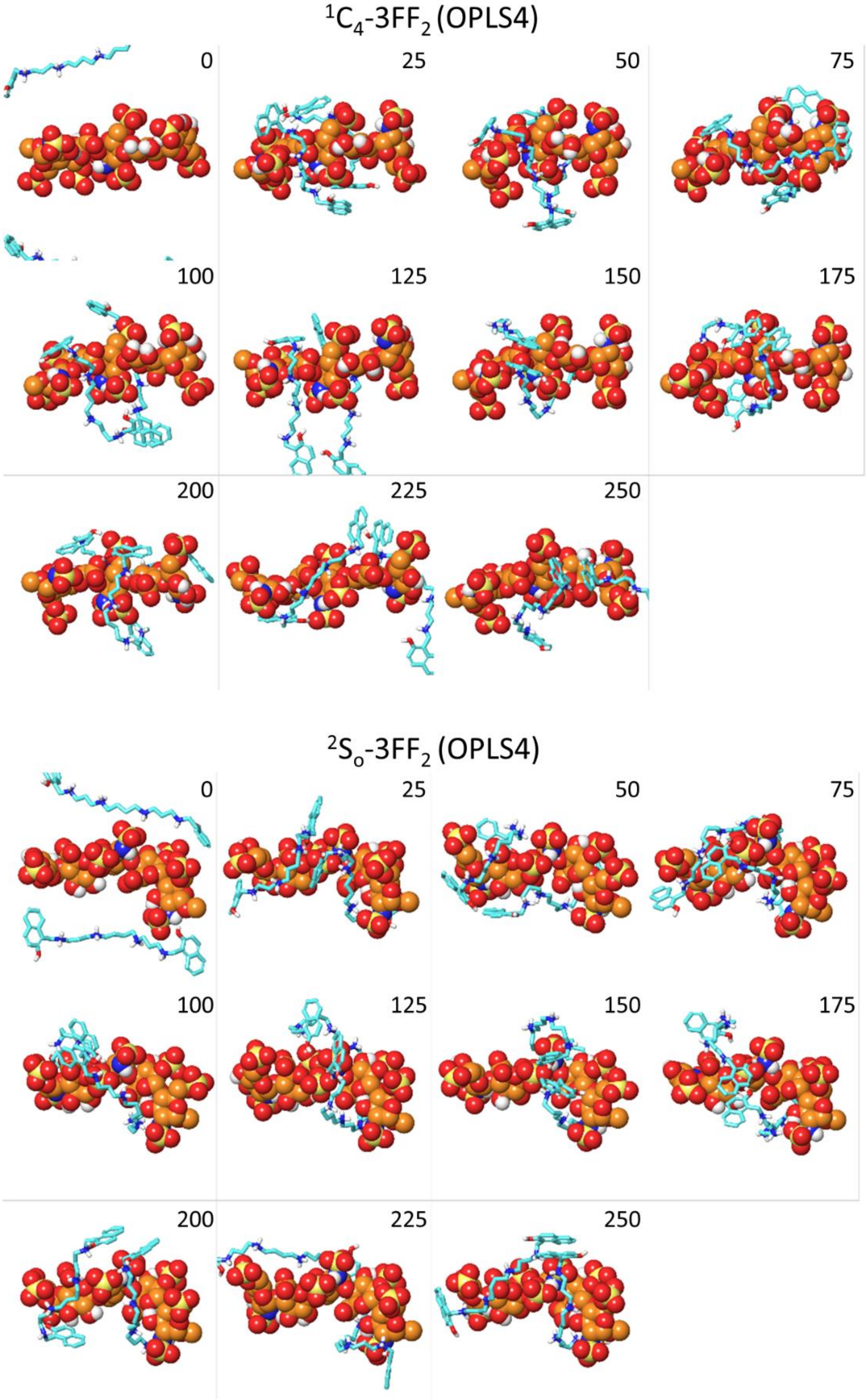

Figure S29. Snapshots of the $^1C_4$-3FF$_2$ and ^2S_O-3FF$_2$ simulations using the OPLS4 force field. The simulation time (ns) is displayed for each snapshot. Fond is shown as spheres (orange C-atoms) and 3FF as sticks (cyan C-atoms). Water molecules, ions and 3FF atoms that are too far from fond are omitted for clarity.

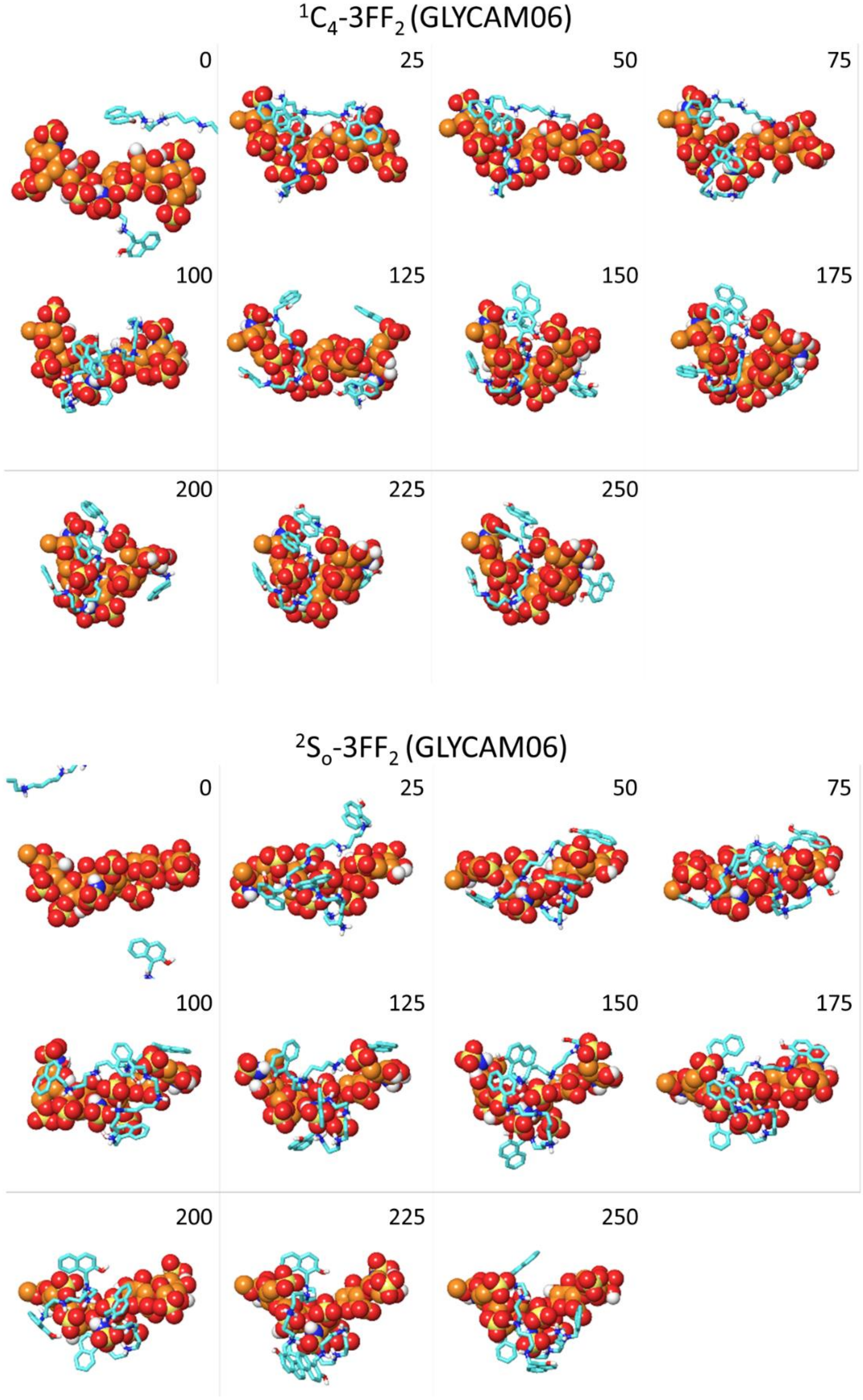

Figure S30. Snapshots of the $^{1}C_4$-3FF$_2$ and $^{2}S_O$-3FF$_2$ simulations using the GLYCAM06 force field. The simulation time (ns) is displayed for each snapshot. Fond is shown as spheres (orange C-atoms) and 3FF as sticks (cyan C-atoms). Water molecules, ions and 3FF atoms that are too far from fond are omitted for clarity.

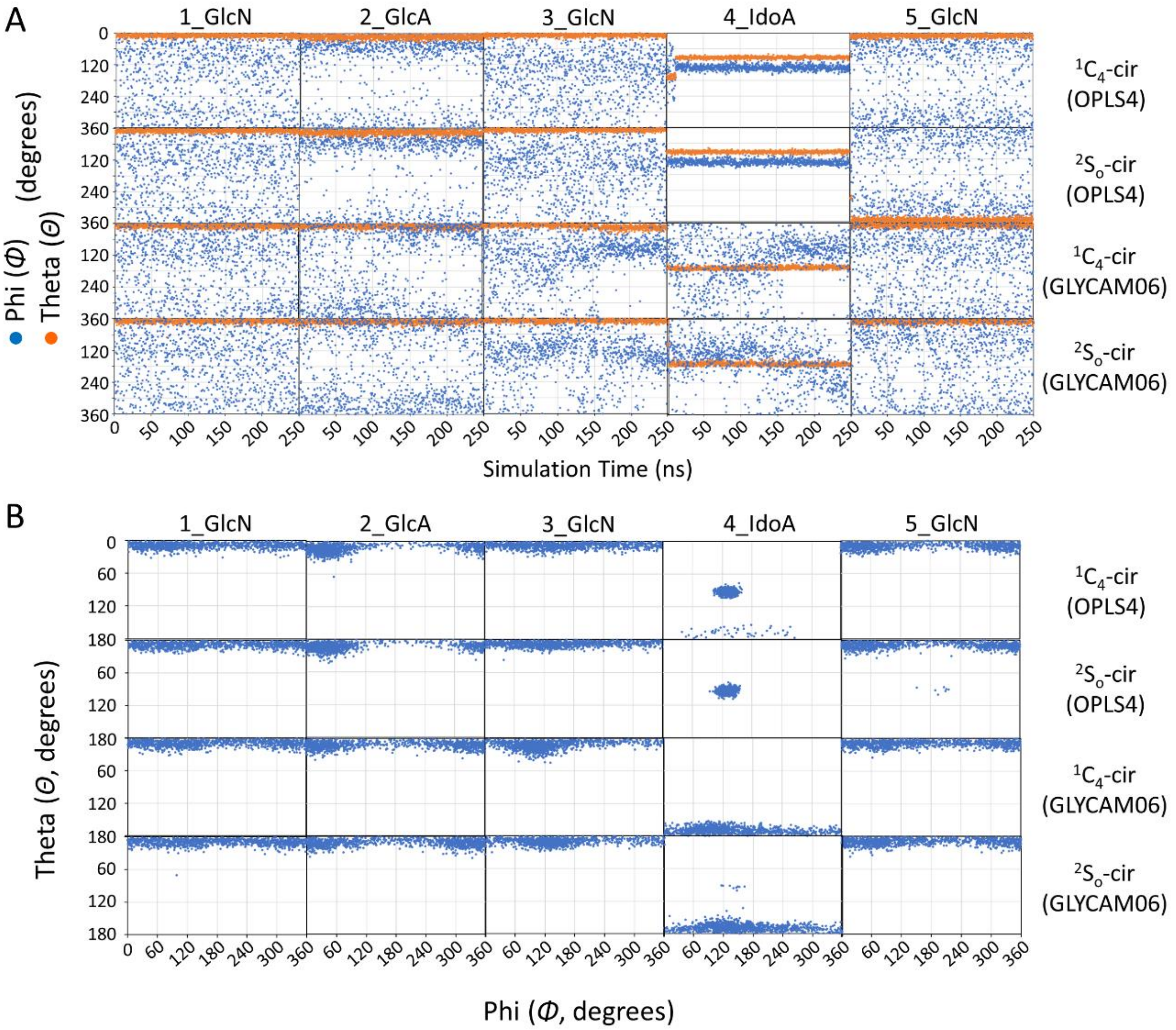

Figure S31. (A) Phi and theta puckering parameters determined for the five rings of fondaparinux during the simulations of $^1C_4$-cir and ^2S_O-cir with the OPLS4 and GLYCAM06 force fields. (B) Theta vs phi puckering parameters representation for the same simulations.

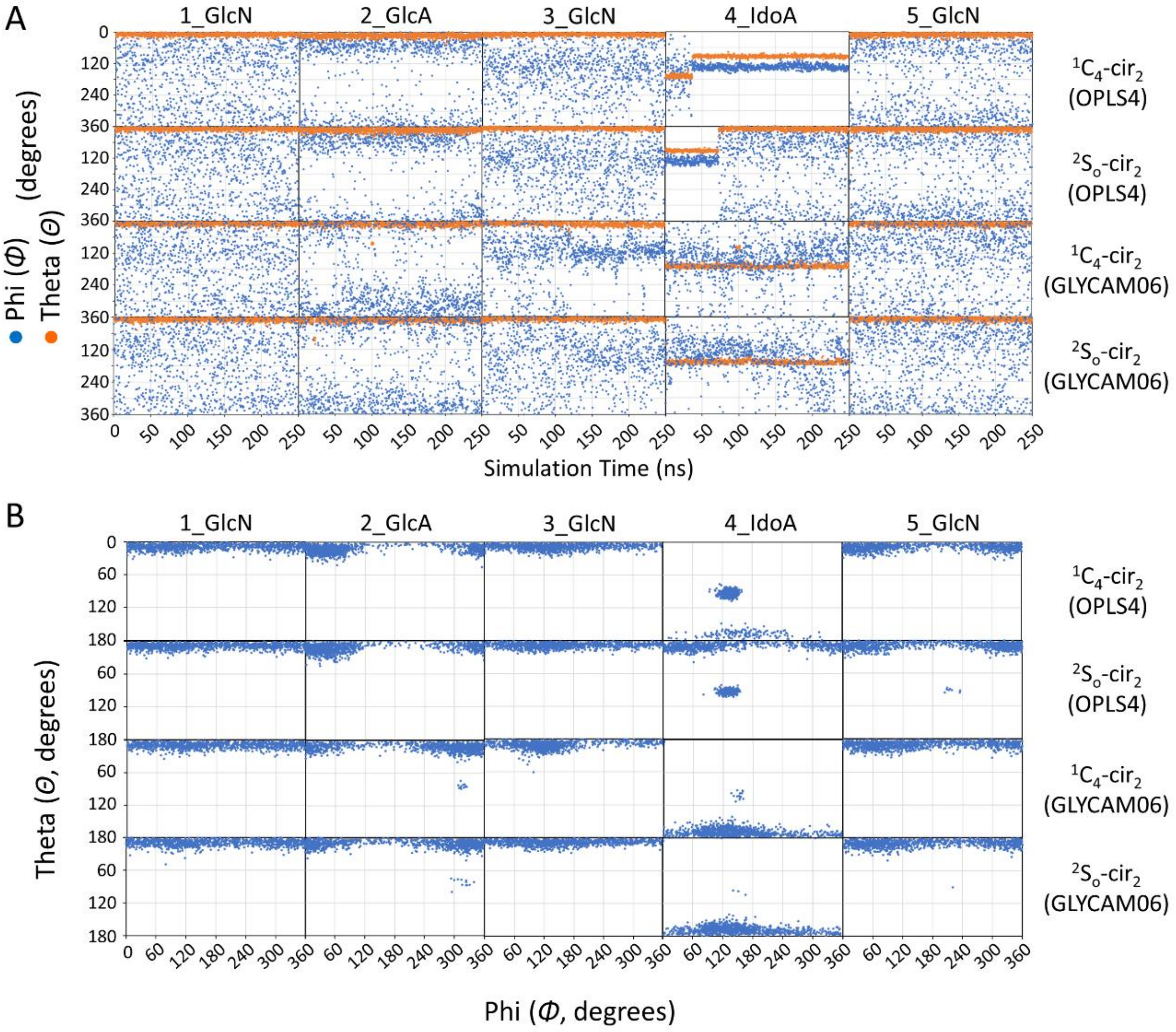

Figure S32. (A) Phi and theta puckering parameters determined for the five rings of fondaparinux during the simulations of $^1C_4$-cir$_2$ and ^2S_O-3cir$_2$ with the OPLS4 and GLYCAM06 force fields. (B) Theta vs phi puckering parameters representation for the same simulations.

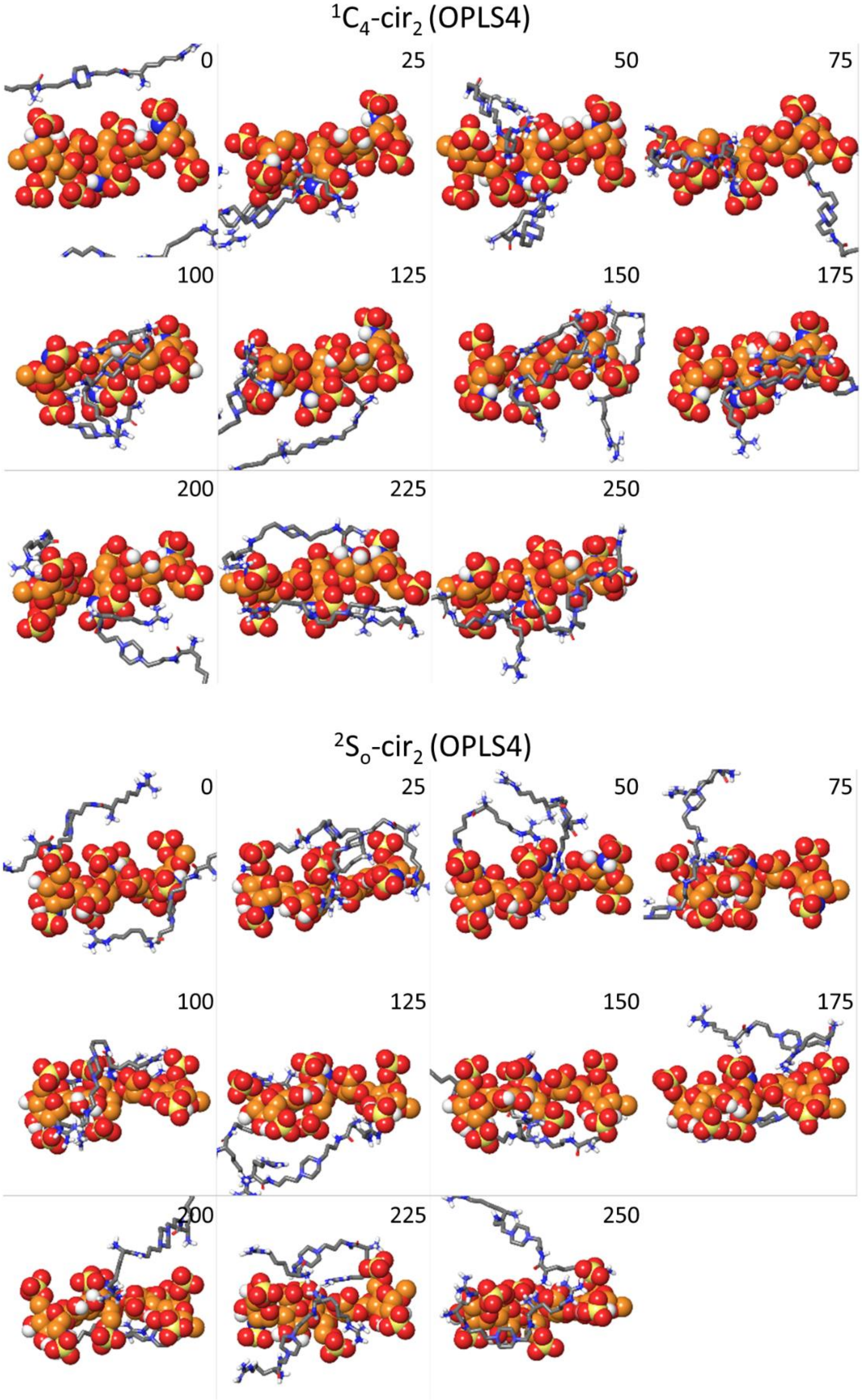

Figure S33. Snapshots of the $^1C_4$-cir$_2$ and ^2S_O-cir$_2$ simulations using the OPLS4 force field. The simulation time (ns) is displayed for each snapshot. Fond is shown as spheres (orange C-atoms) and cir as sticks (gray C-atoms). Water molecules, ions and cir atoms that are too far from fond are omitted for clarity.

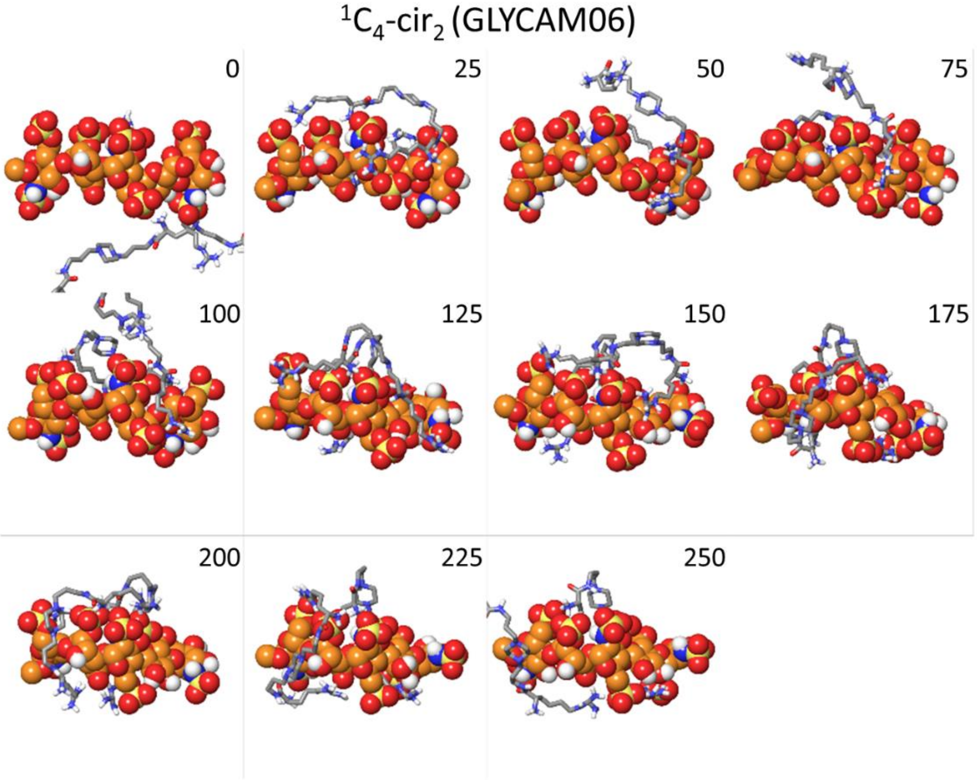

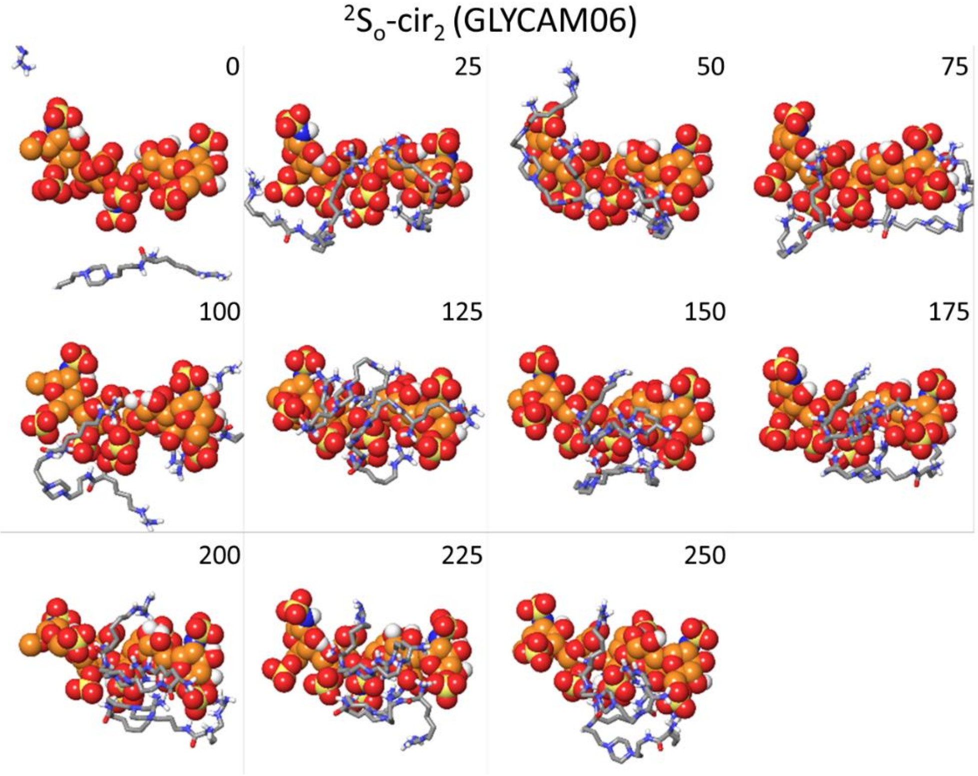

Figure S34. Snapshots of the $^1C_4$-cir$_2$ and ^2S_O-cir$_2$ simulations using the GLYCAM06 force field. The simulation time (ns) is displayed for each snapshot. Fond is shown as spheres (orange C-atoms) and cir as sticks (gray C-atoms). Water molecules, ions and cir atoms that are too far from fond are omitted for clarity.

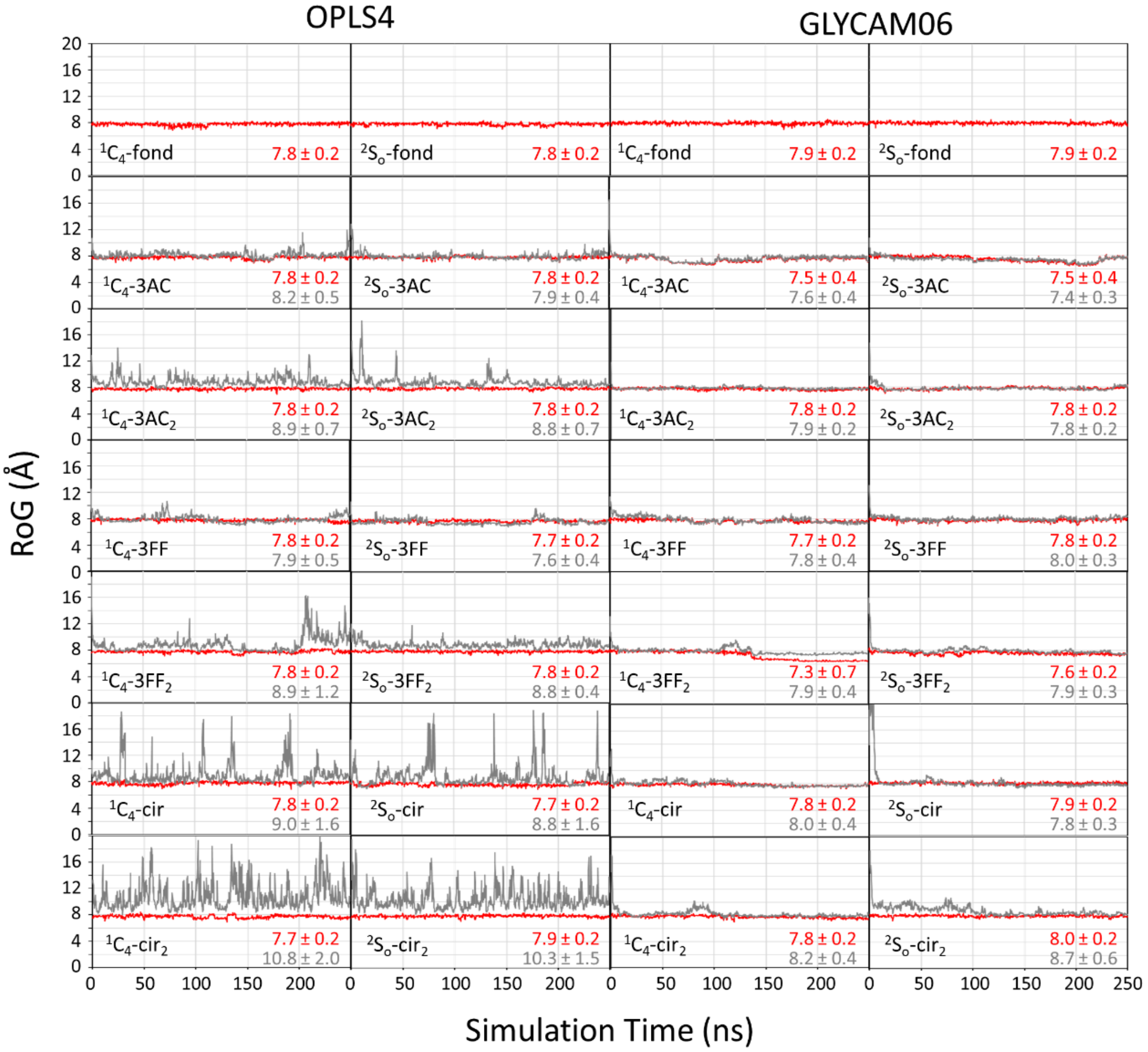

Figure S35. Radius of Gyration (RoG) of fondaparinux (red line) and of its complex with one or two molecules of each ligand (gray line) for the simulations performed. The average RoG values of each trace are shown with the respective color for each graph.

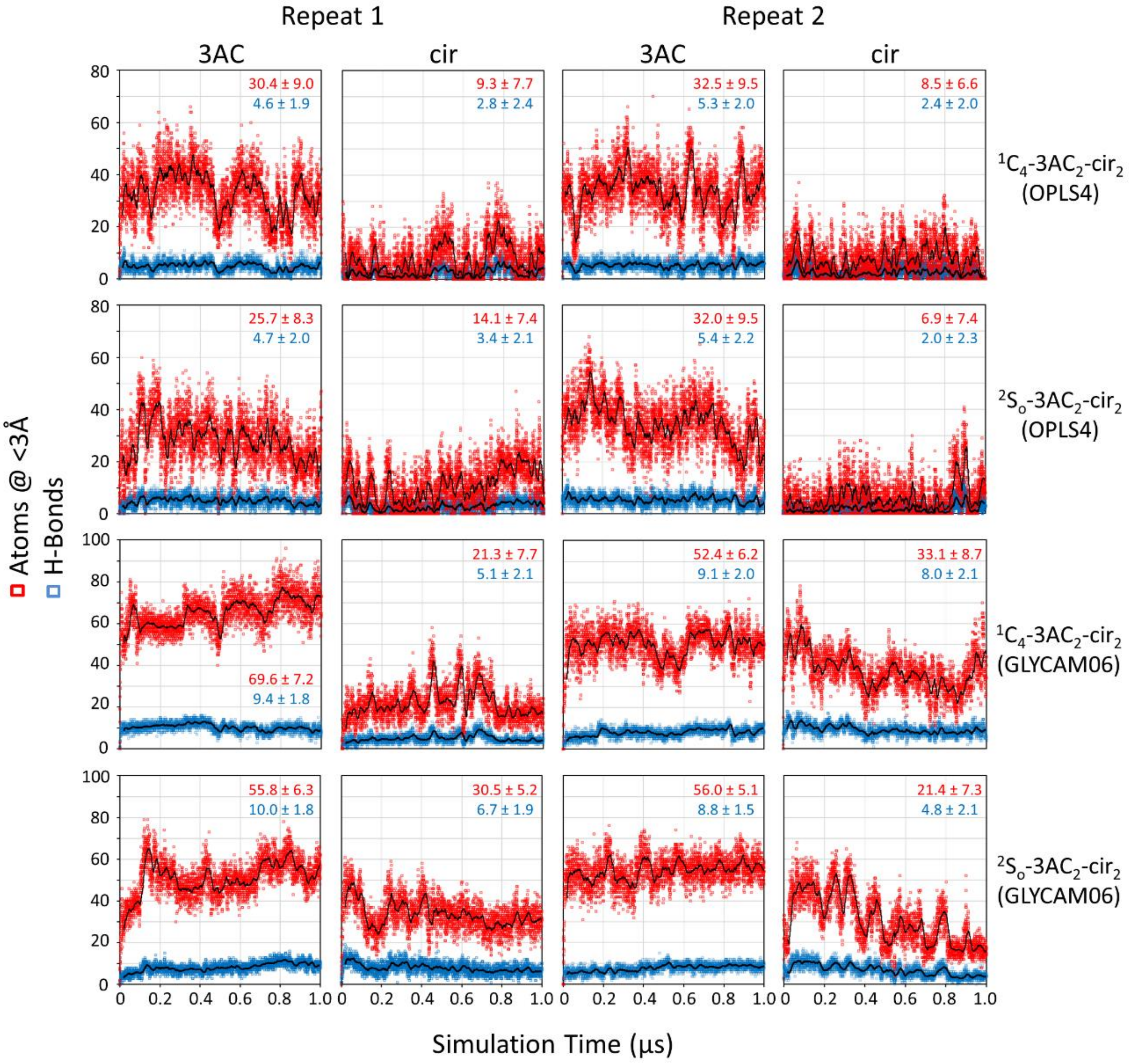

Figure S36. Results of the competition simulations (1 μs at 300 K) performed with one molecule of fond and two molecules of 3AC and cir. Each simulation was performed twice starting from different initial locations for each ligand. Graphs represent the total number of hydrogen bonds between ligands and fond (blue squares), and the total number of ligand atoms within a distance of 3 Å from fond (red squares), vs simulation time. The black lines represent smoothed trend lines. Average values for the last 500 ns are shown with the respective color for each graph.

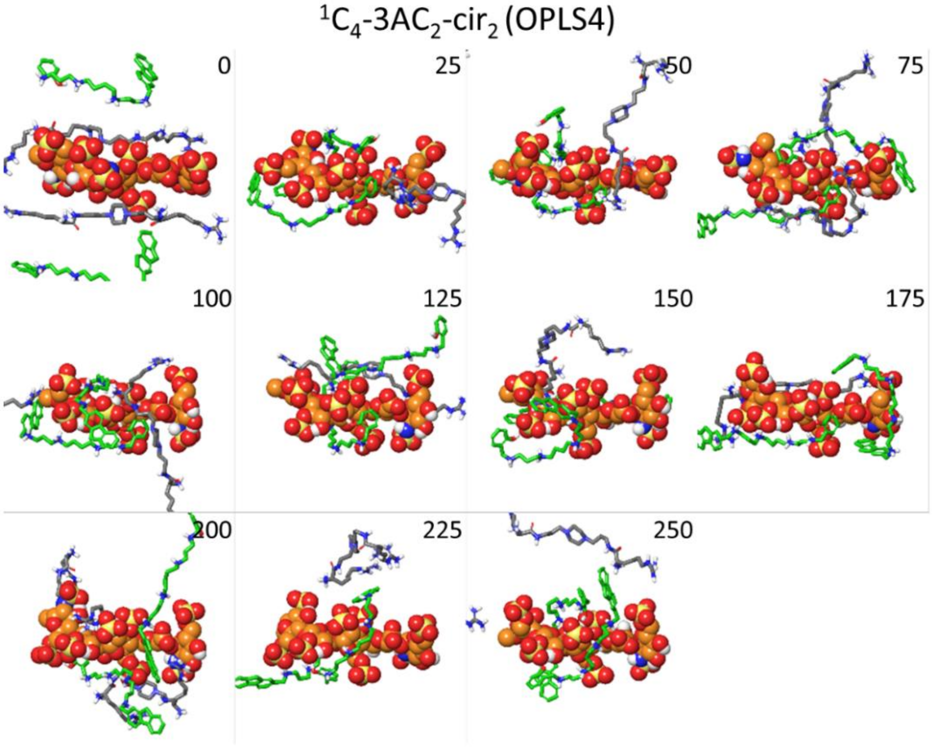

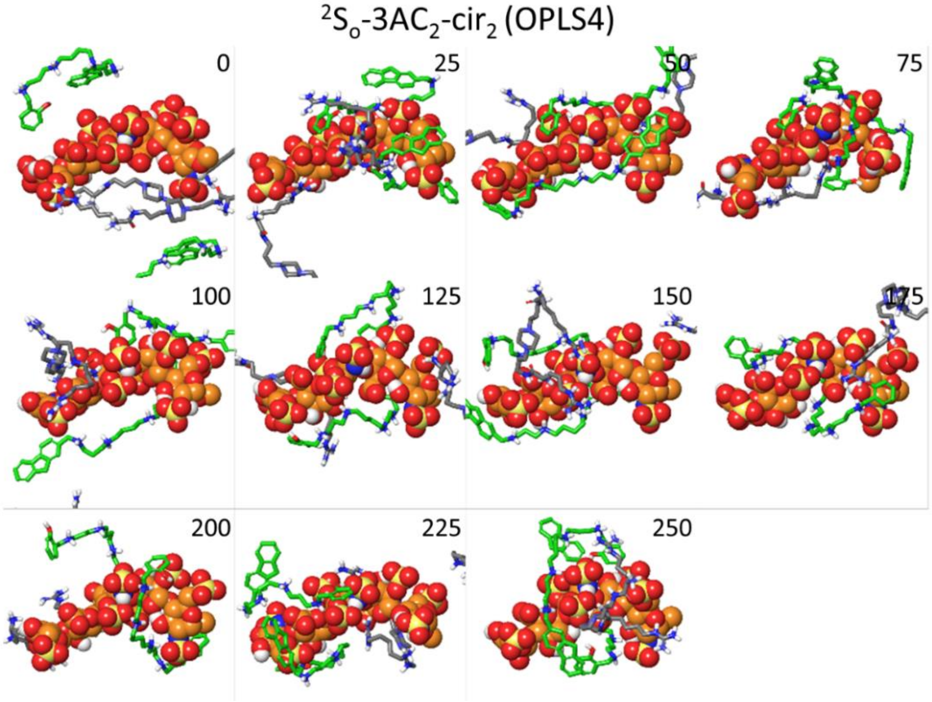

Figure S37. Snapshots of the $^1C_4$-3AC$_2$-cir$_2$ and ^2S_O-3AC$_2$-cir$_2$ simulations using the OPLS4 force field. The simulation time (ns) is displayed for each snapshot. Fond is shown as spheres (orange C-atoms), and 3AC (green C-atoms) and cir (gray C-atoms) as sticks. Water molecules, ions and ligand atoms that are too far from fond are omitted for clarity.

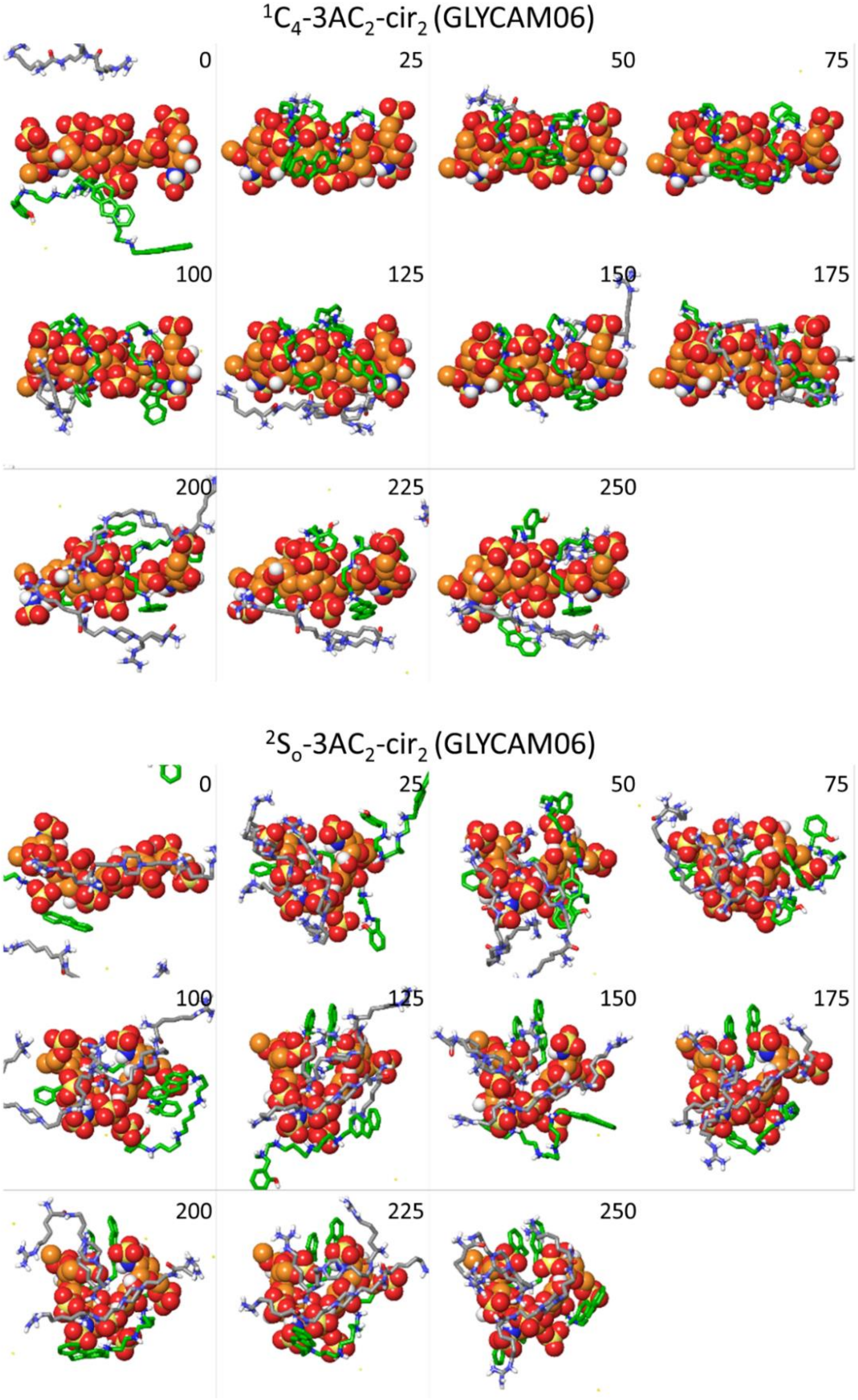

Figure S38. Snapshots of the $^{1}C_4$-3AC_2-cir_2 and $^{2}S_O$-3AC_2-cir_2 simulations using the GLYCAM06 force field. The simulation time (ns) is displayed for each snapshot. Fond is shown as spheres (orange C-atoms), and 3AC (green C-atoms) and cir (gray C-atoms) as sticks. Water molecules, ions and ligand atoms that are too far from fond are omitted for clarity.

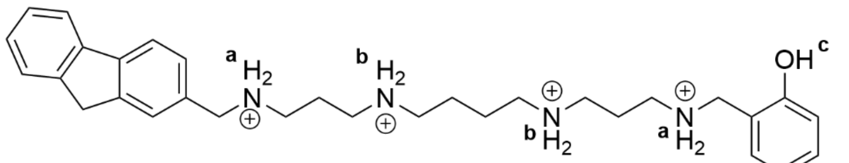

3AC

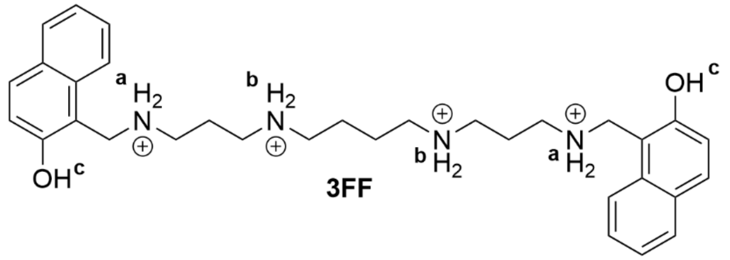

3FF

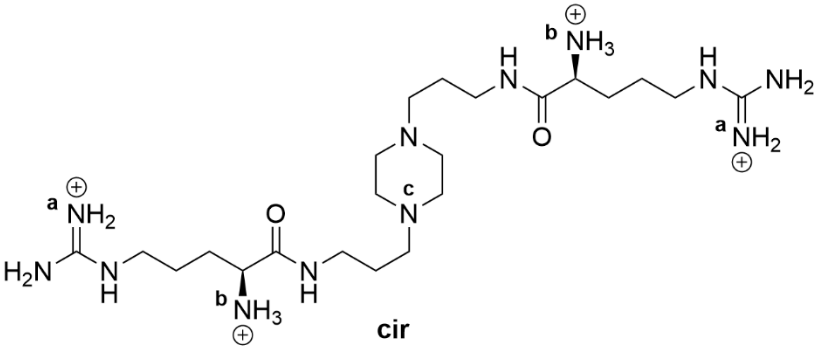

cir

pK_a	a	b	c
**3AC**	8.54±0.27	9.71±0.33	8.64±0.34
**3FF**	7.44±0.73	9.71±0.33	7.60±0.57
**cir**	12.98±0.63	7.82±0.16	7.85±0.21

Figure S39. EPIK pK_a prediction for the protonable groups of 3AC, 3FF and cir, with the assumed charged states used for the molecular dynamics simulations.
